# Widespread alternative exon usage in clinically distinct subtypes of Invasive Ductal Carcinoma

**DOI:** 10.1038/s41598-017-05537-0

**Published:** 2017-07-17

**Authors:** Sunniva Stordal Bjørklund, Anshuman Panda, Surendra Kumar, Michael Seiler, Doug Robinson, Jinesh Gheeya, Ming Yao, Grethe I. Grenaker Alnæs, Deborah Toppmeyer, Margit Riis, Bjørn Naume, Anne-Lise Børresen-Dale, Vessela N. Kristensen, Shridar Ganesan, Gyan Bhanot

**Affiliations:** 1 Rutgers Cancer Institute of New Jersey, 195 Little Albany Street, New Brunswick, NJ 08903 USA; 2 Department of Cancer Genetics, Institute for Cancer Research, OUS Radiumhospitalet, Oslo, 0310 Norway; 3 The K.G. Jebsen Center for Breast Cancer Research, Institute for Clinical Medicine, Faculty of Medicine, University of Oslo, P.O box 1171, Blindern, 0318 Oslo Norway; 4 Department of Clinical Molecular Biology and Laboratory Science (EpiGen), Akershus University hospital, Division of Medicine, 1476 Lørenskog, Norway; 5 Department of Physics, Rutgers University, Piscataway, NJ 08854 USA; 6 Department of Molecular Biology & Biochemistry, Rutgers University, Piscataway, NJ 08854 USA; 7 BioMaPS Institute, Rutgers University, Piscataway, NJ 08854 USA; 8 Department of Surgery, Akershus University Hospital, 1478 Lørenskog, Norway; 9 Department of Breast and Endocrine Surgery, Oslo University Hospital, Ullevål, 0450 Oslo Norway; 10 Department of Oncology, Oslo University Hospital, Radiumhospitalet, Oslo Norway

## Abstract

Cancer cells can have different patterns of exon usage of individual genes when compared to normal tissue, suggesting that alternative splicing may play a role in shaping the tumor phenotype. The discovery and identification of gene variants has increased dramatically with the introduction of RNA-sequencing technology, which enables whole transcriptome analysis of known, as well as novel isoforms. Here we report alternative splicing and transcriptional events among subtypes of invasive ductal carcinoma in The Cancer Genome Atlas (TCGA) Breast Invasive Carcinoma (BRCA) cohort. Alternative exon usage was widespread, and although common events were shared among three subtypes, ER+ HER2−, ER− HER2−, and HER2+, many events on the exon level were subtype specific. Additional RNA-seq analysis was carried out in an independent cohort of 43 ER+ HER2− and ER− HER2− primary breast tumors, confirming many of the exon events identified in the TCGA cohort. Alternative splicing and transcriptional events detected in five genes, *MYO6, EPB41L1, TPD52, IQCG*, and *ACOX2* were validated by qRT-PCR in a third cohort of 40 ER+ HER2− and ER− HER2− patients, showing that these events were truly subtype specific.

## Introduction

Breast cancer is a heterogeneous disease with varying prognosis and response to treatment. Gene expression profiling has confirmed that breast cancer is not one disease, but instead, consists of multiple clinically distinct subtypes^1–5^. Based on the expression levels of the two most important receptors, HER2 and Estrogen Receptor (ER), these subtypes can be summarized as HER2+ cancers, characterized by amplification of *HER2*/*neu* on both ER+ and ER- background, ER+ HER2− cancers, characterized by the expression of ER at no HER2 background, and ER− HER2− breast cancers, when none of these receptors are expressed. Since these receptors are important targets of therapy, patients with ER− HER2− tumors (which also in most cases lack expression of a third progesterone receptor and are called triple-negative breast cancer (TNBC)) have poor treatment options and often poor prognosis. These tumors are mostly of the “basal-like” subtype as classified per whole genome gene expression. The ER+ HER2− cancers, which at large correspond to the “Luminal” breast cancers as identified by clustering of whole genome mRNA expression, can be further subdivided into so-called Luminal A, which are mostly low grade tumors with good prognosis, and Luminal B which are ER+ tumors of mostly high grade, with high expression of proliferative genes and poorer prognosis^1, 2, 4^. These breast cancer subclasses have different natural histories, different responses to treatment and may originate from different types of precursor cells.

Although gene expression profiling has greatly improved our understanding of the clinical diversity of breast cancer, finding novel therapeutic modalities remains a challenge. The first successful strategy was developed to combat ER+ tumors by either blocking the estrogen receptor (tamoxifen) or eliminating its ligand (aromatase inhibitors). Successful development of several treatment approaches that target *HER2*/*neu* has greatly improved outcome for another group of breast cancers^6^. However, although several promising pathways have been identified as being critical, there is no clinically validated target for ER− HER2− breast cancers. Also within the ER+ HER2− group, the most favorable and frequent type of breast cancer, the response to treatment is highly variable^7–11^. Thus there is a pressing need to understand more about the basic biology of these tumors.

Transcriptome sequencing allows researchers to interrogate features of the genome not readily assayed by either array-based gene expression profiling or genomic sequencing^12, 13^. These include the differential expression of splice variants encoded by individual genomic loci. Differential exon usage can lead to different functional gene products arising from a single genomic locus, which adds greatly to the diversity of gene products encoded by the genome. Several studies have found that cancers can have different patterns of exon usage of individual genes when compared to normal tissue, suggesting that alternative splicing may play a role in the tumor phenotype^14, 15^. A recent report analyzing RNA sequencing data from 17 breast cancer specimens showed significant diversity of splicing events in TNBC, non-TNBC and HER2+ breast cancers, with both exon skipping events and alternative promoter usage identified^16^. These data show that alternative transcript use may play a role in breast cancer biology. Since this was a study limited to a small sample size, and given the large inter tumor heterogeneity, it might reveal only splice variants and transcripts specific to few patients.

To characterize alternative splicing and transcriptional events in breast cancer subtypes on a more global scale, we used RNA-sequencing data from The Cancer Genome Atlas (TCGA) Breast Invasive Carcinoma (BRCA) cohort (1097 tumor samples of which 112 had available normal sample counterpart), as well as RNA-seq from an independent cohort of 43 breast cancer patients. Differential exon usage was studied to reveal alternative splicing and transcription events present in the three distinct clinical subtypes of breast cancer: ER+ HER2−, ER− HER2−, and HER2+. We found widespread alternative exon usage when comparing the three subtypes to normal breast samples and distinct differences between the subclasses. The specific exons that undergo differential splicing or transcription in the clinical subgroups were identified, showing that many genes deregulated in cancer undergo subtype specific transcriptional and/or post-transcriptional events. Alternative transcriptional and splicing events in five genes, *IQCG, EPB41L1, TPD52, MYO6*, and *ACOX2*, were validated by Taqman qRT-PCR in an independent patient cohort consisting of ER+ HER2− and ER− HER2− patient samples. This study identifies alternative transcription events among clinically distinct breast cancer subgroups in a large patient cohort.

## Results

### Identification of alternative exon usage

Our goal was to identify differentially spliced genes and alternative transcription events in an unprecedented large set of human breast carcinomas and observe the differences between clinical subtypes. To that end RNA-sequencing data from the TCGA BRCA cohort was obtained and the analysis was focused on invasive ductal carcinomas with reliable data (493 ER+/HER2−, 157 ER− HER2−, 97 HER2+), as well as 112 normal breast samples (NBS) as described in materials and methods.

**Figure 1:**
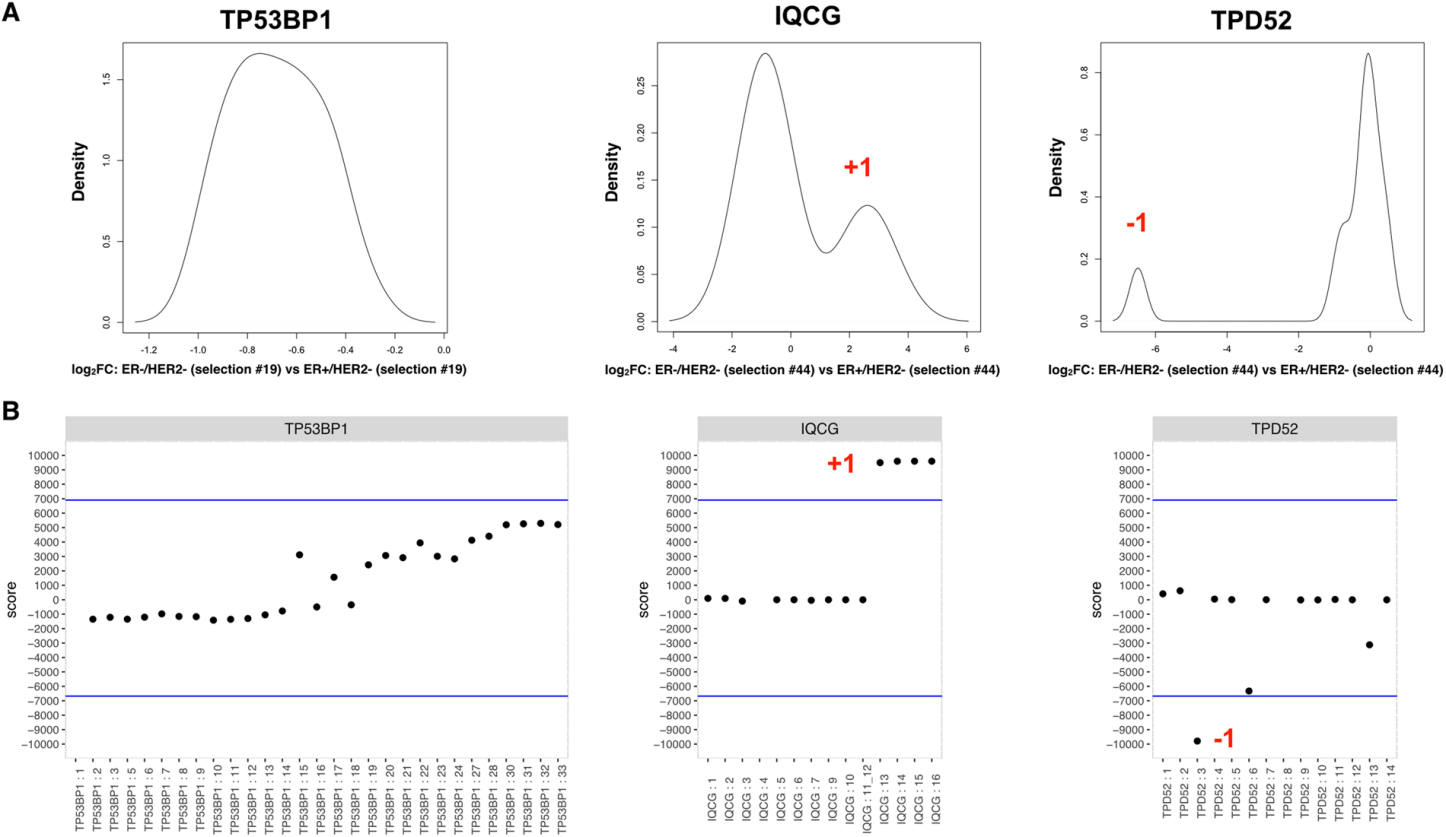
Method of analysis. (**A**) The difference in mode expression between exons in two biological groups (ER+ HER2−, ER− HER2−, HER2+, and NBS) was computed as the log_2_ FC for each exon of a gene and the distribution (density plot) of log_2_ FC for all exons in each gene was plotted. The density plots shown for *TP53BP1, TPD52*, and *IQCG* compares one selection of 60% of ER+ HER2− samples to one 60% selection of ER− HER2− samples, and was repeated 100 times for each pairwise comparison. The highest peak represents an overall scaling effect, affecting the majority of exons. Genes not affected by differential splicing will have only one peak, as shown for *TP53BP1*. The remaining exons, i.e. those in peaks with smaller amplitude, were identified as exons that are differentially spliced or transcribed. Peaks to the right (*IQCG*) indicate exons with greater log_2_ FC compared to the exons in the central peak. Peaks to the left of the central peak (*TPD52*) are exons with smaller log_2_ FC than the exons in the central peak. Log_2_ FC was calculated for random selections of 60% of all samples within a biological class 100 times. For each test an exon was called either +1, −1, or 0, so that each exon was tested 10.000 times, and the total score for each exon was determined. The total scores for each exon in the three genes, *TP53BP1, IQCG*, and *TPD52* when comparing ER− Her2− and ER+ HER2− samples are shown in (**B**). Exons with scores exceeding 3 standard deviations (blue line) were called in the final analysis.

There are a number of published methods on using RNA-sequencing to infer alternative splicing and alternate transcript usage^17–19^. Exon expression data was calculated by BEDtools for all samples in the cohort, as per hg19, and was used to identify differentially spliced genes and alternate transcription events directly, without the assembly of fragments into specific transcripts. An outline of the method is presented in Supplementary Fig. S1. In brief, samples were classified into clinical subtypes based on focal copy number of ERBB2, and expression of ESR1 (Supplementary Fig. S2 and Supplementary Table S3). The coordinates of the exons were mapped to exons of known genes and lincRNAs in hg19. After applying a minimum expression threshold to eliminate exons with very low expression levels, the expression of each exon was normalized by the median exon expression for each sample, to account for possible systematic errors (Supplementary Fig. S4 and Methods). After this, all exons were compared pairwise between each two studied clinical subtype classes. To reduce the number of false positives due to high intra class variability, the analysis was repeated 100 times for each exon, resulting in 10,000 pairwise comparisons per exon for each pair of classes. Plotting the expression of all exons each time using the “density” function in R, the mode expression of each exon in a random selection of 60% of the samples within each class was determined (i.e the most likely expression value(s)). Exons with no clear mode (see Supplementary Fig. S5) were discarded. A valid mode expression of at least 5 exons per gene in one class was required for final analysis, leaving a total of 175,025 exons in 13,050 genes. Exons with valid mode of expression were used for pairwise comparisons between two clinical classes, where modes of expression in each class were subtracted from each other to obtain the difference in expression (roughly equivalent to log_2_ fold change (log_2_ FC)) (Supplementary Fig. S6). The distribution of log_2_ FC for each exon was further plotted for each gene. A few selected genes, *TP53BP1, IQCG* and *TPD52* are used for illustration of the method in Fig. 1. In the absence of differential splicing the expression difference should be approximately the same for all exons, and so the distribution of expression difference will be unimodal, where down-regulated genes will have a unimodal negative log_2_ FC (Fig. 1A, *TP53BP1*), up-regulated genes unimodal positive log_2_ FC, and genes with similar expression in both classes will have log_2_ FC close to 0 for all exons. In the presence of differential splicing however, a few exons will be either relatively up-regulated, or relatively down-regulated compared to the fold change for the rest of the exons of that gene. In both cases, the distribution of the expression difference will be multimodal (bimodal in simplest cases). For up-regulated exons, a minority of exons will form one or more smaller peaks to the right of the global maximum (Fig. 1A, *IQCG*, and Supplementary Fig. S7), and for down-regulated exons, a minority of exons will form one or more smaller peaks to the left of the global maximum of the distribution (Fig. 1A, *TPD52*). Most exons will belong to the peak containing the global maximum, and we labeled these exons as 0, and exons belonging to smaller peaks to the right/left of the global maximum were called +1/−1 respectively. The distribution of the sum of these 10,000 numbers obtained by pairwise comparison using 60% of samples in each class 100 times for each exon are shown in Supplementary Fig. S8. Based on these distributions, only exons with scores exceeding a conservative threshold of 3 standard deviations were called as differentially spliced and the genes harboring the exons that met this cutoff were considered differentially spliced or differentially transcribed. The scores for all exons in *TP53BP1, IQCG*, and *TPD52* when comparing ER− HER2− to ER+ HER2− tumors, including the final called exons are shown in Fig. 1B. An illustration of different splicing and transcriptional events called +1/−1 is included as Supplementary Figure S9.

### Alternative exon usage among clinical groups in the TCGA BRCA cohort compared to NBS

To identify alternative exon usage in clinical subtypes of invasive ductal carcinoma, the tumor samples were stratified into ER+ HER2− (n = 493), ER− HER2− (n = 157), and HER2+ (n = 97) tumors based on copy number of ERBB2, and mRNA expression of ESR1 (see Supplementary Fig. S2 and Methods for details). Each clinical group was then compared pairwise to the NBS, identifying genes subjected to alternative splicing and transcription. Exons with a final call of +1 (to the right of the main peak in the density distribution) are exons with higher FC compared to most exons of the gene, when comparing the FC for each exon between tumor and normal samples. These significant exons indicate events such as exon inclusion, expression of alterative first and last exons in the tumor subtypes, as well as exon skipping events in the normal samples. Exons called as −1 are those with lower FC compared to the rest of the exons in the gene and final scores below 3 standard deviations in the distribution between tumor and normal samples (see above and Supplementary Fig. S8). These are exon skipping events in the tumor subtypes, and exon inclusion events in the normal samples. The number of exons and genes in each tumor subtype that differed from the NBS are presented in Table 1, and all called events are available in Supplementary Table S10.

**Table 1:** Number of exons and genes found to have differential exon usage among the three clinical subtypes compared to NBS.

	Higher log_2_ FC (+1) tumor vs NBS		Lower log_2_ FC (−1) tumor vs NBS	
	Exon	Gene	Exon	Gene
All tumors	916	680	1,212	950
ER+ HER2−	3,094	1,940	3,122	2,116
ER− HER2−	3,005	1,827	2,814	1,848
HER2+	2,732	1,826	3,253	2,300

**Figure 2:**
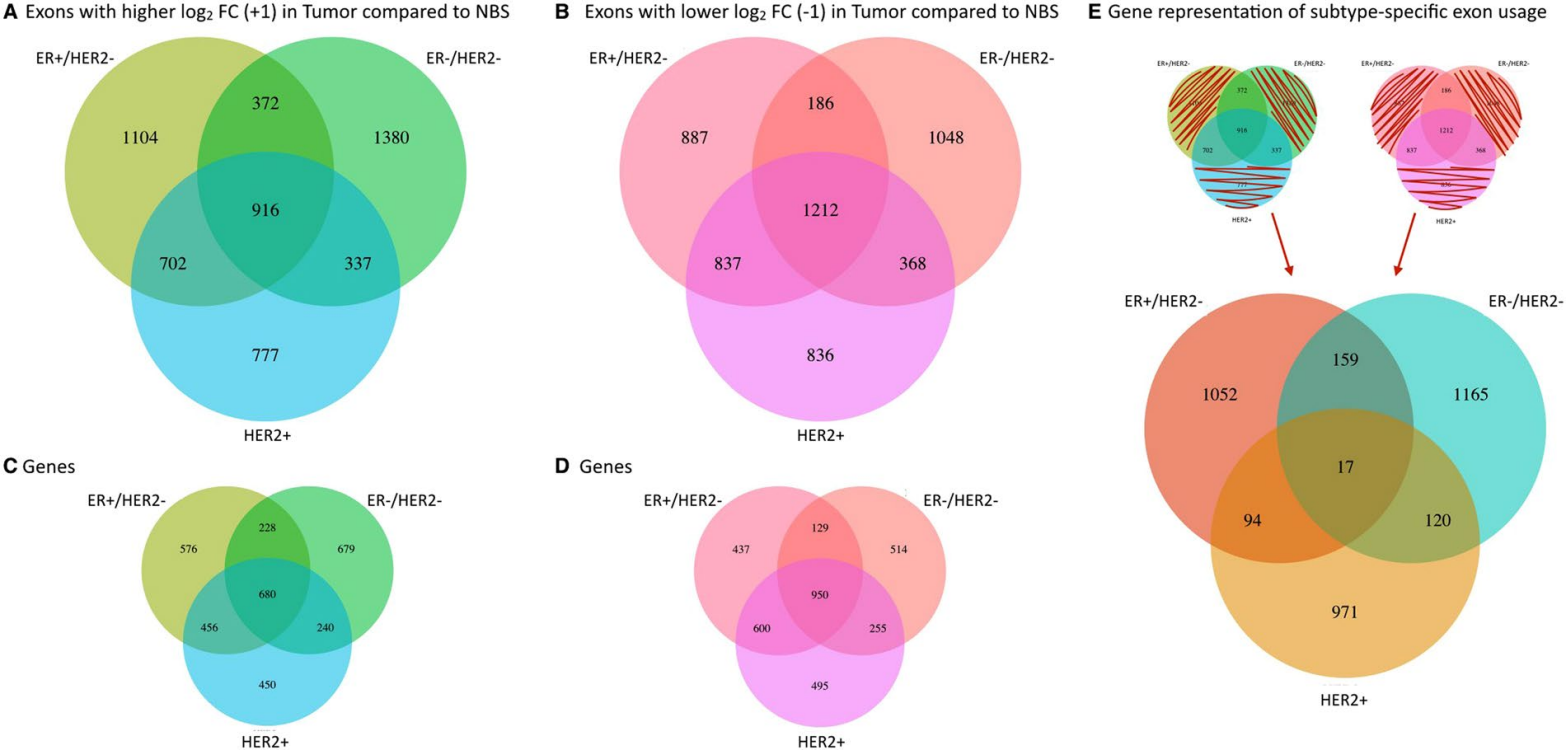
Alternative exon usage in tumor subtypes compared to NBS. The number of exons identified as differentially spliced or transcribed in each tumor class compared to NBS with (**A**) higher FC, and (**B**) lower FC. The number of genes represented by the called exons is shown for each tumor subtype in (**C** and **D**). Many genes include both higher and lower FC exons. The number of genes representing the exons that are unique to subtype include both genes with differential usage of unique exons in one subtype only, as well as exons with different usage in two subtypes (+1 in one subtype, −1 in another) (**E**). The overlapping genes undergo distinct transcriptional and/or post-transcriptional events of specific exons in a subtype specific manner.

*A total of 5588 exons showed higher FC in one or more tumor subtype compared to the NBS* (~3% of all exons analyzed), 916 (16%) of which were common to all tumor subtypes (Fig. 2A). A total of 1104 exons were found with higher FC exclusively in the ER+ HER2− patients, 1380 in the ER− HER2− subtype, and 777 exons in the HER2+ patient group respectively. *Similarly*, *a total of 5374* (~*3*% *of all exons analyzed*) *exons showed lower FC in one or more tumor subtype compared to the NBS*, and 1212 (23%) of these exons showed alternative splicing and/or transcription in all three tumor classes (Fig. 2B).

*The overlap among tumor subtypes increased when alternative exon usage was investigated on the gene level* to 21% for up-regulated exons and 28% for down-regulated exons (Fig. 2C and D). The 916 exons with higher FC in all three tumor subtypes belonged to 680 genes, whereas 1212 exons in 950 genes had lower FC in all tumor classes compared to the normal samples (Table 1) making these changes overall breast cancer specific. The number of genes showing alternative exon usage in breast cancer subtypes was lower than the number of exons identified as differentially spliced, indicating that many genes are subjected to differential usage of more than one exon.

*Subtype specific exon usage was evident* as many exons showed differential usage in only one subtype (1631 (HER2+), 1991 (ER+ HER2−), and 2428 (ER− HER2−), +1/−1 exons combined, Fig. 2A and B). The number of genes representing the exons that are unique to subtype are shown in Fig. 2E. The overlapping genes (390 in two or more subtypes) undergo distinct transcriptional and/or post-transcriptional events of specific exons in a subtype specific manner.

### Alternative exon usage in differentially expressed genes in the TCGA BRCA cohort compared to NBS

Gene expression patterns differ substantially from NBS, as well as between tumor subtypes^2^. We further analyzed whether the genes with differential exon usage observed between tumor classes and NBS were among the differentially expressed genes, and whether the proportion of alternatively spliced/transcribed genes was different in the three tumor subtypes when compared to normal samples. A gene was considered with overall higher/lower gene expression in the tumors when the absolute maximum in the density plot of log_2_ FC of exons in this gene was >1, or <−1, respectively, in tumor subtypes compared to NBS (Supplementary Table S10).

**Figure 3:**
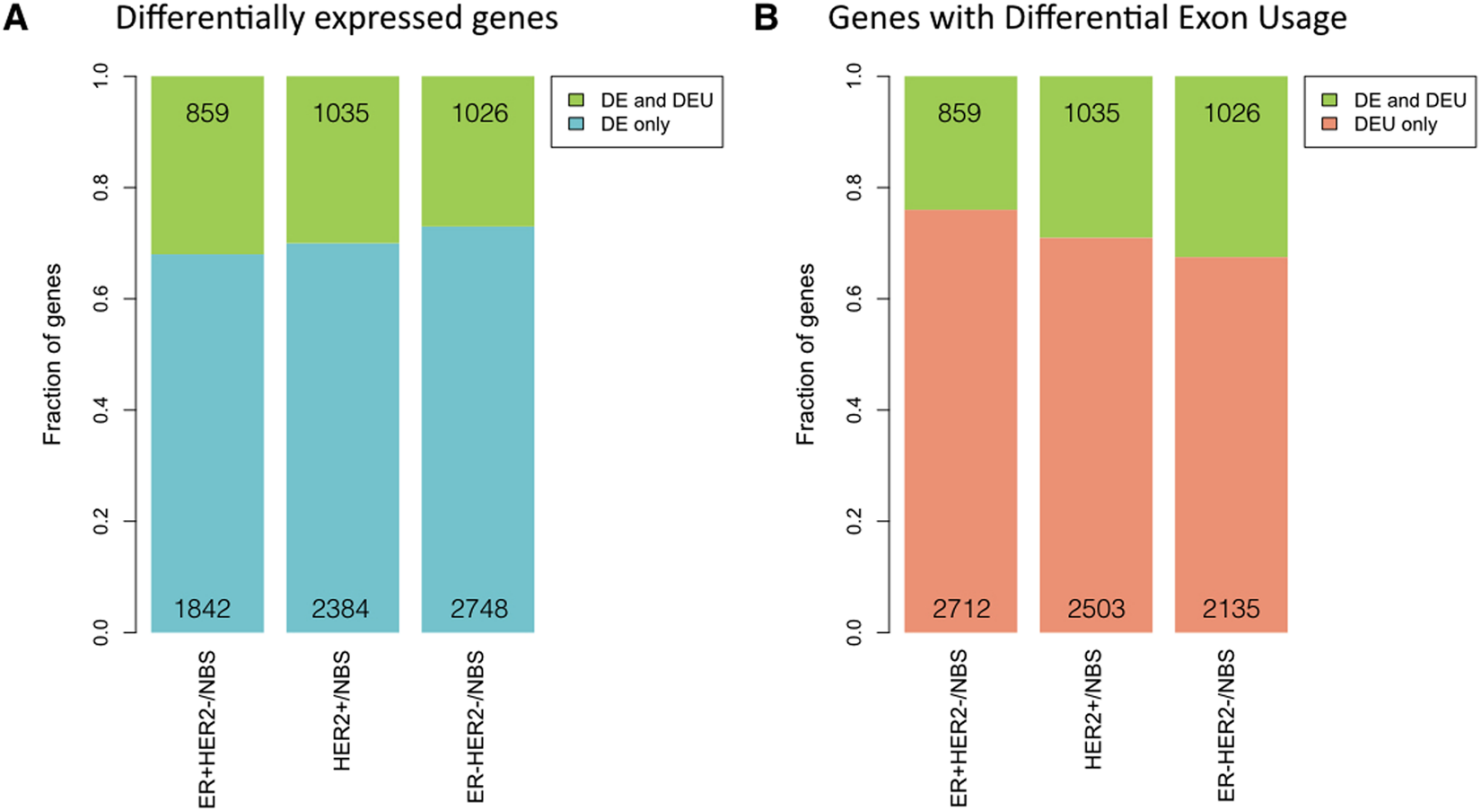
Alternative exon usage in differentially expressed genes in tumor subtypes compared to NBS. The fractions of overall differentially expressed genes with differential exon usage (DEU), or differential gene expression (DE) only are shown in (**A**). The numbers of genes in each category are included in the bar graph. The fraction and numbers of genes with DEU that; either show DEU only, or are also part of overall DE genes are shown in (**B**).

*In genes with overall differential expression in the tumor subtypes compared to NBS*, the highest frequency of genes subjected to differential exon usage was observed in the ER+ HER2− subtype. Although ER− HER2− tumors had the highest number of genes that were overall differentially expressed, they had lowest frequency of genes with alternative splicing/transcriptional events when compared to NBS (Fig. 3A and Table 2, p = 1.52E-04, Chi-square test).

**Table 2:** Number of genes with differential exon usage in genes with higher, lower, and similar expression in the three clinical subtypes compared to NBS.

	Genes with +1 Exons	Genes with −1 Exons	Total genes with DEU	Unspliced genes	Tot genes
**log**_**2**_ **FC >1 tumor vs NBS**					
ER+ HER2−	239	331	526	1,025	1,551
ER− HER2−	254	444	664	1,744	2,408
HER2+	254	427	624	1,374	1,998
**log**_**2**_ **FC < −1 tumor vs NBS**					
ER+ HER2−	146	217	333	818	1,151
ER− HER2−	199	192	362	1,004	1,366
HER2+	215	233	411	1,012	1,423
**1 > log**_**2**_ **FC > −1 tumor vs NBS**					
ER+ HER2−	1,443	1,515	2712	6,289	9,001
ER− HER2−	1,231	1,074	2135	5,511	7,646
HER2+	1,232	1,504	2503	5,684	8,187

*When analyzing all the genes subjected to alternative splicing in the three subtypes* the ER+ HER2− subtype had again the highest fraction of genes with differential exon usage, despite the highest number of genes, with little or no overall differential gene expression (76% of the genes) (Fig. 3B, p = 9.46E-14, Chi-square test). The ER− HER2− tumors showed the highest fraction of differential exon usage in differentially expressed genes. Overall the three subtypes showed different patterns of gene regulation; in the ER+ HER2− subtype, a smaller fraction of the genes were deregulated on the overall, but differential exon usage contributed to a large fraction (66%) of all gene regulation, while the ER− HER2− subtype had a higher fraction of differential exon usage in differentially expressed genes. For all three tumor subtypes the majority of differentially used exons belonged to genes with low or no overall differential gene expression, which confirms that splicing and alternative transcription is an additional level of transcriptional regulation in tumorigenesis.

### Identification of alternative exon usage among clinical subgroups of invasive ductal carcinoma

**Figure 4:**
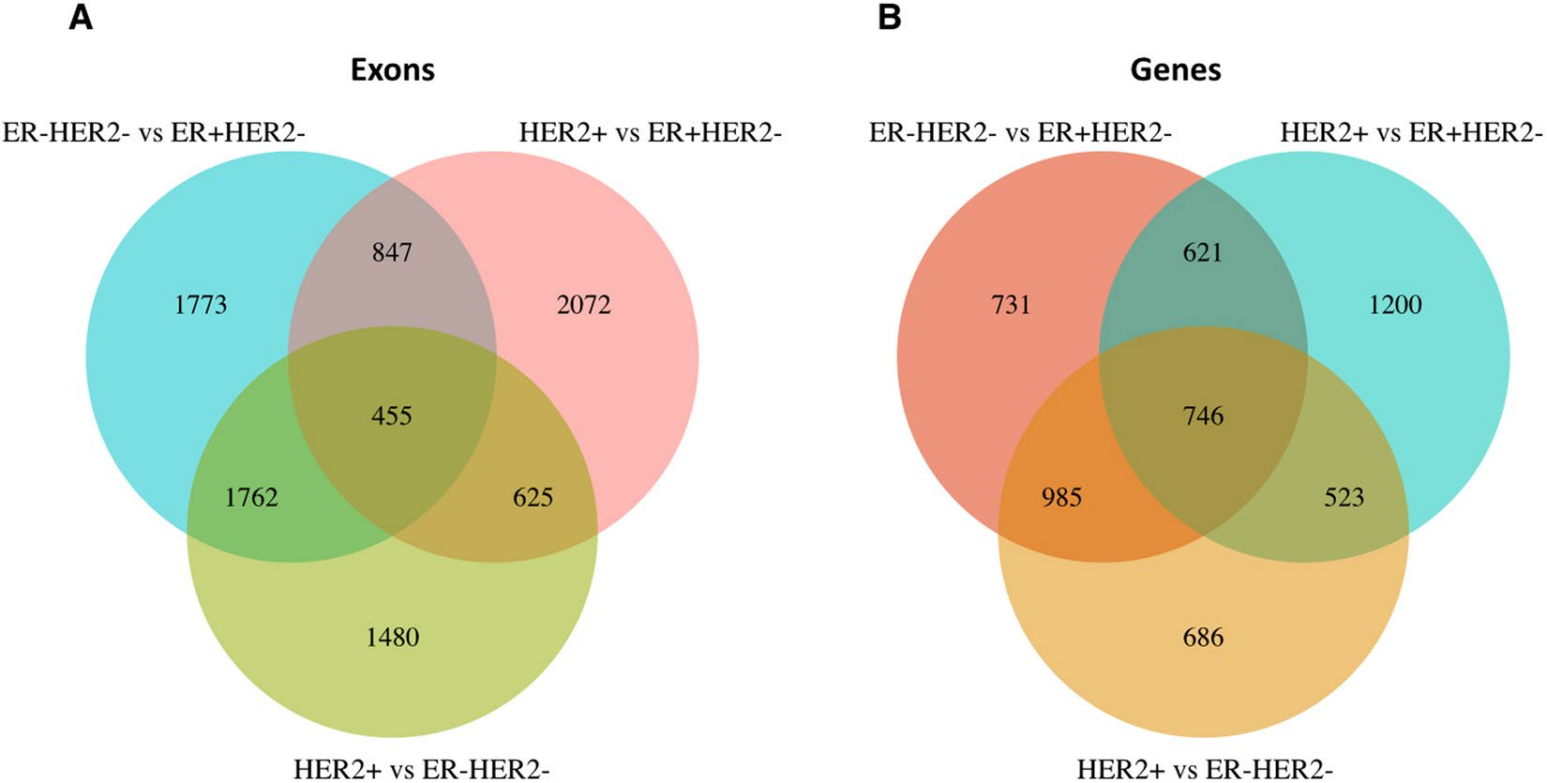
Alternative exon usage among subtypes. The number of differentially spliced or transcribed exons (**A**) and genes (**B**) when all three tumor subtypes were compared.

We further analyzed the patterns of alternative exon usage among the three breast cancer subtypes by the same method as above, and identified 1,773 exons with differential use exclusively when comparing the ER− HER2− to ER+ HER2− subtypes (Fig. 4A). A summary of all exons identified as differential among subtypes is shown in Table 3. Most of the 3,083 genes with alternative exon usage when comparing the ER− HER2− and ER+ HER2− subtypes (except 731 genes) were also found to have an alternative exon usage in the other subtype groups (Fig. 4B), but in many of the same genes different exons were subjected to subtype specific transcriptional and splicing events. 13.5% of the genes identified showed alternative exon usage among all the three subtypes.

**Table 3:** Exons and corresponding gene number with alternative usage among subtypes.

	Exons	Genes
ER− HER2− vs ER+ HER2−	4,837	3,083
HER2+ vs ER+ HER2−	3,999	3,090
HER2+ vs ER− HER2−	4,322	2,940

### Additional analysis of alternative exon usage among ER+ HER2− and ER− HER2− patients in an independent patient cohort

We performed RNA-sequencing of 43 breast tumor samples collected from the Rutgers Cancer Institute of New Jersey, and Oslo University Hospital^20^ (Radium/Rutgers) to further investigate alternative exon usage in the ER+ HER2− and ER− HER2− subgroups. Expression values for all exons were generated using DEXseq^18^, and differential splicing and transcription analysis was performed as described above, with a few modifications due to sample size (see Methods for details), for the genes identified as differentially spliced or transcribed between ER+ HER2− and ER− HER2− in the much larger TCGA cohort. Of the 2598 exons identified with higher FC between the two subtypes 366 exact exons were also identified as +1 in the Radium/Rutgers cohort, (expected by chance = 183, p < 6.296e-39, hyper geometric test). 249 (expected by chance = 138, p < 5.081e-20, hyper geometric test) of the 2239 exons with lower relative FC showed the same expression pattern in the independent data set. All exons identified in both datasets are included in Supplementary Table S11.

Five genes with no previously described splice variants or isoforms in breast cancer, were chosen for further characterization of the alternative events observed. The five genes chosen represent different ranges of confidence scores, from genes with very high score (*MYO6*), moderately high scores (*TPD52, EPB41L1*, and *IQCG*), to scores close to the 3 standard deviation cutoff (*ACOX2*).

Erythrocyte membrane protein band 4.1 (*EPB41L1*) is known to mediate interactions between the cytoskeleton and the plasma membrane in erythrocytes^21^. The chr20:34797410-34797820 exon of *EPB41L1* showed lower log_2_ FC (−1) in the ER+ HER2− group when compared to NBS, and higher (+1, final score 9778) in ER− HER2− compared to ER+ HER2−, although the FC was only 2 fold in the TCGA cohort. This exon skipping event was also detected in the independent dataset (Supplementary Fig. S12). Myosin 6 is an actin-based molecular motor protein with functions in endocytosis and intracellular transport^22^. The exon in position chr6:76608090-76608128 was identified as lower (−1) in ER− HER2− when compared to NBS, and lower (−1, final score −9990) in the ER− HER2− than the ER+ HER2− subtype in the TCGA data set. Reads spanning the exon in location chr6:76608090-76608128 of *MYO6* was observed in the primary data of the validation cohort (Supplementary Fig. S12), suggesting that this is an exon skipping/inclusion event.

Tumor protein D52 (*TPD52*) is a putative oncogene located on 8q21 and involved in vesicular transport^23, 24^. Exon chr8:80992550-80993010 of TPD52 showed lower log_2_ FC in the ER− HER2− subtype when compared to NBS, and lower when the ER− HER2− and ER+ HER2− subtypes were compared (final score 9789). Manual inspection of this region indicated that this is an alternative first exon, and reads originating in this exon were observed in the independent data set (Supplementary Fig. S12). IQ motif containing G (*IQCG*) is a calmodulin interacting protein involved in cellular Ca++ signaling^25^. Four exons in *IQCG* were called due to higher log_2_ FC in the ER− HER2− patient group compared to NBS, and as higher (+1, scores between 9499 and 9599) when the ER− HER2− and ER+ HER2− subtypes were compared in the TCGA cohort. Inspection of the region preceding the chr3:197639546-197640155 exon revealed reads in the intronic region in the independent data set (Supplementary Fig. S12), indicating that this is a transcript with an intronic start site.

Intronic reads were also detected in the region preceding the chr3:58512193-58512383 exon of *ACOX2* in the Radium/Rutgers data set (Supplementary Fig. S12). Although these exons were not identified in the additional data set, the fact that they were identified in the larger cohort (close to the 3 st. dev. cut off scores from 7301–7587), and validated experimentally (see below) shows that marginal events such as this can be identified only when the number of samples is large enough. 6 exons 3′ of the above position were called as higher FC exons in ER+ HER2− as well as the HER2+ subtypes when compared to NBS, as well as lower (−1) in the ER− HER2− compared to ER+ HER2− subtype in the TCGA cohort.

### Validation of five differential transcription events by qRT-PCR in an independent patient cohort

The five genes described above were analyzed by qRT-PCR to validate the differential expression patterns among ER+ HER2− and ER− HER2− patients in an independent set of tumor samples from the well characterized MicMa cohort^20^. The genes represented different alternative transcription and splicing events, and primers and probes were designed to specifically detect the events in question for each gene. All probes and primer sequences are included in Supplementary Table S13, and detailed illustrations of the positioning of probes, and splicing events are included in Supplementary Figure S14.

**Figure 5:**
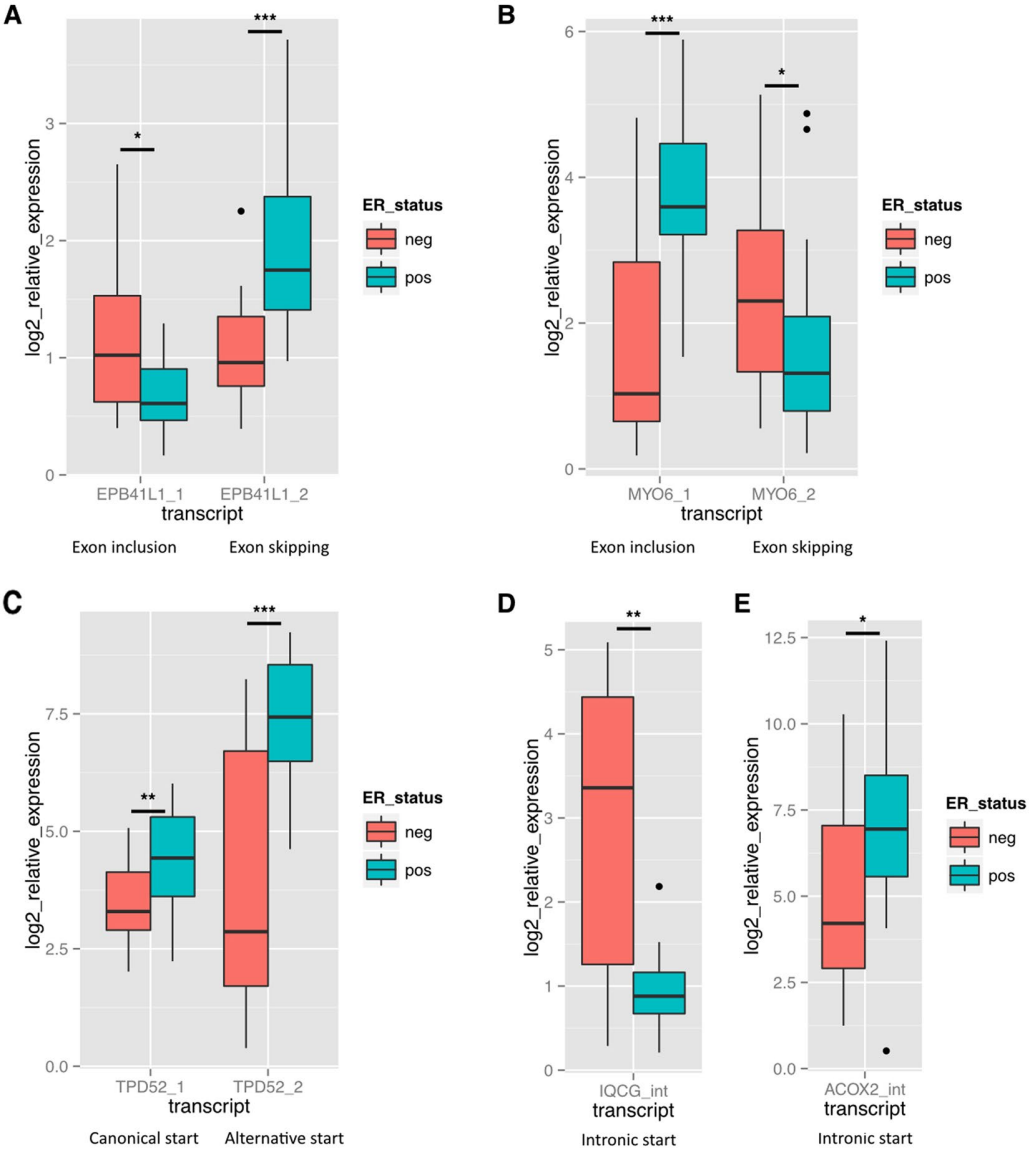
Validation of splicing and transcriptional events by Taqman qRT-PCR in an independent patient cohort. PCR primers and Taqman probes were designed to specifically detect transcriptional events in 5 genes in an independent cohort consisting of 20 ER+ HER2− and 20 ER− HER2− patients from the MicMa cohort. Log2 expression is shown relative to normal breast RNA. PMM1 and RPL32 were used for normalization. The following events were measured: (**A)** The inclusion and skipping of exon chr20:34797410-34797820 in *EPB41L1*. (**B**) The inclusion and skipping of exon chr6:76608090-76608128 in *MYO6*. (**C**) An alternative start exon (chr8:80992550-80993010) and the “canonical” start exon (chr8:81083660-81083836) of *TPD52*. (**D**) The intronic start transcript of *IQCG*. (**E**) The intronic start transcript of *ACOX2*. Association to ER status was assed using the Wilcoxon’s rank-sum test, ***p < 0.0005, **p < 0.005, *p < 0.05.

Exon skipping events were identified in *EPB41L1* as well as in *MYO6*. These events were validated using primers that would detect both the exon inclusion, as well as the exclusion event. The chr20:34797410-34797820 exon of *EPB41L1* showed more exon inclusion in the ER− HER2− than in ER+ HER2− (more exon skipping) group. Both of these events were validated in the independent cohort (Fig. 5A). The exon at location chr6:76608090-76608128 of *MYO6* was expressed higher (more exon inclusion) in the ER+ HER2− and lower (exon skipping) in the ER− HER2− group. This event was also significantly different when analyzed by qPCR (Fig. 5B).

Exon chr8:80992550-80993010 of *TPD52* was expressed higher in the ER+ HER2− patient group. Primers detecting this exon, as well as the first exon of the canonical transcript of *TPD52* were used to validate the event in the independent cohort (Fig. 5C). The differential use of first exon chr8:80992550-80993010 was significant also by qRT-PCR. A significant difference was detected the in expression of the canonical transcript as well, although overall expression of this transcript was lower.

The intronic start variants of both *IQCG* and *ACOX2* were validated using forward primers starting in the intronic sequences. The variant of *IQCG* was higher in the ER− HER2− compared to the ER+ HER2− group, while the *ACOX2* variant was identified as higher in the ER+ HER2− group. Both events were validated by qRT-PCR (Fig. 5D and E respectively). In addition, the intronic sequences observed in the primary RNA-seq data were validated by Sanger sequencing (data not shown).

*We compared the results for the validated genes from our methodology for both datasets to a previously published method* (*DEXseq*)^18^. DEXseq results based on raw p-values showed that the validated exons in *TPD52, MYO6*, and *EPB41L1*, as well as 2 of the 4 exons in *IQCG* were significant in the TCGA cohort (Supplementary Table S15). However only the exons in *TPD52* and *MYO6* were significant in DEXseq after multiple testing correction (FDR < 0.05) as the adjusted p-values were >0.05 when the five genes were analyzed with all genes on chr22. In the smaller Radium/Rutgers dataset the validated exons in *TPD52, MYO6*, and *EPB41L1* were found significant in a whole genome analysis (Supplementary Table S16). The experimentally validated exons in *IQCG* and *ACOX2* were not identified by DEXseq in either dataset. Of the 61 exons that were found to be differentially expressed on Chr22 in the TCGA dataset, only 3 were also identified as significantly differentially expressed in the validation cohort when analysis was carried out for genes on Chr22 only (Supplementary Tables S15 and S16). This comparison suggests that our methods are in some cases consistent with DEXseq and in other cases, identify additional real splice variants that are missed by DEXseq.

### Pathways and molecular functions influenced by genes with alternative exon usage

To evaluate whether the genes affected by the alternative exon usage identified were related to specific biological functions or pathways, we used the ToppGene suite^26^ for gene set enrichment analysis of the candidate genes in the TCGA cohort.

*We analyzed the genes with alternative exon usage common to all the tumor subtypes and different from NBS*, and the top significant GO terms and pathways are presented in Table 4. The top five biological functions included mitotic cell cycle, cytoskeleton organization, and RNA splicing.

**Table 4:** Significantly enriched biological processes and pathways affected by alternative exon usage common to all three tumor subtypes.

Biological Process	Name	p-value (FDR B&H)	Genes with alternative exon usage	Genes in Annotation
GO:0007049	cell cycle	1.03E-11	223	1766
GO:0022402	cell cycle process	6.64E-10	179	1385
GO:0007010	cytoskeleton organization	1.79E-09	155	1164
GO:0000278	mitotic cell cycle	1.79E-09	140	1016
GO:0008380	RNA splicing	1.44E-08	71	402
GO:1903047	mitotic cell cycle process	1.44E-08	128	931
GO:0051726	regulation of cell cycle	2.11E-08	135	1008
GO:0043484	regulation of RNA splicing	4.98E-08	31	109
GO:0006397	mRNA processing	5.16E-08	78	479
GO:0000398	mRNA splicing, via spliceosome	8.38E-08	57	305
**Pathway**	**Name**	**p-value (FDR B&H)**	**Genes with alternative exon usage**	**Genes in Annotation**
198843	mRNA processing	7.94E-05	31	136
125136	Spliceosome	7.94E-05	30	131
105765	Cell Cycle, Mitotic	4.34E-04	62	416
137994	CDC42 signaling events	8.62E-04	19	71
477132	Cell-Cell communication	4.10E-03	26	131

*The genes with exons with higher FC between tumor and normal* showed enrichment for genes involved in mitotic cell cycle (GO:0000278) and cell division (GO:0051301), as well as functions related to cytoskeletal organization (GO:0007010), both actin (GO:0030036) and microtubule cytoskeleton (GO:0015630), and cell junctions (adherens junction GO:0005912, anchoring junction GO:0070161).

*For genes containing exons with lower FC between tumor and normal*, the top 5 enriched biological processes were all related to RNA splicing and mRNA processing (GO:0006397, GO:0000398, GO:0000377, GO:0000375, GO:0008380), including 28 genes related to the spliceosomal complex (GO:0005681). The top significant GO terms identified as affected by alternative exon usage, in total and separated into +1 and −1 exons in all three tumor classes compared to NBS are included in Supplementary Table S17.

*A large number of exons showed alternative usage specifically in one tumor subclass*. These are genes where unique exons were affected in one subtype, as well as genes where the same exons showed different usage in two subtypes (+1 in one subtype, −1 in an other subtype) (see Fig. 2E). Some of the biological processes that were enriched based on these genes with subtype specific exon usage are presented in Table 5, and all results are included in Supplementary Table S17. For instance the genes with alternative exon usage specific to the ER− HER2− tumor class showed significant enrichment in additional exons in genes related to mitotic cell cycle, and in several genes encoding subunits of hemidesmosome complexes. Both the ER− HER2− and ER+ HER2− tumor classes were enriched for genes involved in integrin signaling, but the ER− HER2− tumors showed particular enrichment for genes that code for subunits of the integrin complex specifically, such as *ITGA2*, *ITGA3, ITGA6*, and *ITGB4*. The ER+ HER2− tumor class showed particular enrichment of genes related to regulation of small GTPase mediated signal transduction, more specifically Ras and Rho signaling pathways, and regulation of GTPase activity in general, while the HER2+ tumors had alternative exon usage enriched for genes involved in neurogenesis and chromatin modification.

**Table 5:** Significantly enriched biological processes and pathways affected by subtype specific alternative exon usage.

ER− HER2−			ER+ HER2−			HER2+		
Biological Process	Name	p-value (FDR B&H)	Biological Process	Name	p-value (FDR B&H)	Biological Process	Name	p-value (FDR B&H)
GO:0007049	cell cycle	1.35E-06	GO:0051056	regulation of small GTPase mediated ﻿﻿signal transduction	3.12E-09	GO:0022008	neurogenesis	1.09E-07
GO:0000278	mitotic cell cycle	7.38E-06	GO:0007265	Ras protein signal transduction	2.25E-08	GO:0016568	chromatin modification	1.27E-07
GO:0030056	hemidesmo some	2.32E-05	GO:0043087	regulation of GTPase activity	2.37E-07	GO:0030182	neuron differentiation	1.81E-06
GO:0008305	integrin complex	1.89E-04	GO:0007266	Rho protein signal transduction	1.73E-06	GO:0006325	chromatin organization	7.85E-06

## Discussion

Next generation transcriptome sequencing is a powerful technique to identify, characterize, and measure the relative abundance of exons and different transcripts generated from each gene loci^12, 13, 27^. We applied transcriptome sequencing and analytic methods to identify alternative exon usage among classes of human breast cancers, and comparing each class to expression in normal breast tissue. Our analysis shows that exon usage from a single genomic locus is highly variable, which contributes to gene product diversity in breast cancer. We found a large set of genes with differential exon usage in one specific clinical subgroup, in addition to the many exons found with differential use in all three tumor classes when compared to NBS. Our results suggest that the specific expression of alternative transcripts from the same genes plays a role in the biology of human breast cancer subtypes.

Previous studies of alternative splice variation in breast cancer have either attempted to characterize variation in tumor versus normal^16, 28, 29^, or have focused on the identification of exon-skipping events^30^, or compared cell lines using exon microarrays^31^. Although exon arrays can detect differential expression of single exons, variation in hybridization efficiency between probes makes the identification of splicing events challenging. Eswaran *et al*.^16^ performed transcriptome analysis of 17 breast cancers, and noted significant differences in splicing signatures in TNBC, non-TNBC and HER2+ breast cancers when compared to NBS (non tumorigenic). We have extended this analysis to include 747 samples from the TCGA BRCA cohort, and through identification of differential exon usage observe events in genes identified previously, in addition to identifying a number of new events in these clinical breast cancer subtypes. As far as we know, this is the first comprehensive analysis of splicing and differential transcriptional events in the TCGA BRCA cohort.

Transcript assembly is part of many RNA-seq analysis pipelines, and facilitates the quantification of specific isoforms. The assembly of short reads from RNA-sequencing experiments into full transcripts poses a great challenge, and can be performed either *ab initio* ^32^, or guided by a reference genome (such as Ensembl or RefSeq)^17, 27^. These assemblies will include uncertainties, as many reads will be shared between multiple transcripts^19^. Specific exons can be a part of a single or multiple isoforms expressed from the same gene. By performing differential exon analysis it is possible to detect high variance in expression of single or multiple exons, without knowledge of the exact transcript isoform(s). This method allows for sensitive detection of differential use of exons, identifying events possibly not significant or detectable on a transcript level, and avoiding the uncertainties introduced by transcript assembly. We have developed a conservative method to identify alternative splicing and transcription events when two clinical groups of samples are compared. We show that alternative splicing and transcription are widespread events, affecting a large number of genes in all three breast cancer subtypes. The large number of events detected (in many cases several per gene) indicates that differential exon usage from the same gene locus is highly dynamic. Differential usage of a single exon was the most frequently type of alteration detected in all three tumor classes. These events include exon skipping as well as alternative first and last exons. The exact exonic sequences that show differential usage should be further investigated with regards to protein functional domains, presence of regulatory signals, as well as their association to clinical outcome. This will help identify the splicing and transcription events important in driving tumorigenesis, as well as tumor heterogeneity, knowing that transcription of a single exon can have dramatic functional effects^33, 34^.

Close to 20% (19, 4%) of all the exons with differential usage when each subtype was compared to NBS were common to all three classes. These alternate transcription events are not related to subtype, rather, some of these events could contribute to cancer phenotypes shared by all subtypes. This was evident in the enrichment analysis where top enriched biological functions of genes with differential exon usage shared among all tumor classes versus NBS were mitotic cell cycle (GO:0000278) and cell division (GO:0051301). Proliferation of fully differentiated cells is considered an important driver of carcinogenesis^35^, and includes deregulation of cell cycle control. Among the genes with differential use of exons were Cyclin D3 (*CCND3*), Cell Division Cycle 25B (*CDC25B*), *CDC25C, CDC42*, and Cyclin Dependent Kinase-1 (*CDK1*), *CDK3*, and *CDK6*. *CDK1* is a Ser/Thr protein kinase, which is essential for G1/S and G2/M phase transitions of eukaryotic cell cycle. High *CDK1* activity has been associated to poor prognosis in breast cancer patients^36^. *CCND3* also induces progression through the G1 phase of the cell cycle, through regulation of *CDK4* and *6*, and has also been linked to poor prognosis in breast cancer^37^. *CDC25C* is a phosphatase that is involved in the entry into mitosis. Different isoforms of *CDC25C* have been described in breast cancer^38^, and expression of a full-length isoform has been shown to be up-regulated in prostate cancer^39^. Three exons with little or no expression in NBS were called as +1 in all three tumor classes, indicating higher expression of full length *CDC25C* in breast cancer.

Interestingly, mRNA splicing (GO:0000377, GO:0000375, GO:0008380), the spliceosome (GO:0006397), and mRNA processing (GO:0000398) were the top enriched biological processes for genes with exons showing both higher and lower FC between tumor and NBS. The list of genes includes several arginine-serine rich (SR) proteins, a key family of splicing factors, and many members of the small nuclear ribonucleoprotein family, which associate with spliceosomal RNA (*SNRNP70, SNRPB, SNRPD3*). Of the SR proteins affected by alternative exon usage are *SRSF4* and *SRSF5*. These transcripts contain elements that are fully conserved among human, mouse, and rat genomes, so called ultraconserved elements^40^. These exons have been called ‘poison cassette exons’ because they contain early in-frame stop codons, and transcripts expressing these exons are subject to nonsense-mediated decay (NMD)^41^. In all three tumor classes the inclusion of these exons in *SRSF4* and *SRSF5* are lower than in NBS (−1 in Tumors), an indication of stabilization of these SRSF transcripts in the tumors. It has previously been noted that splicing factors are highly regulated by post-transcriptional processing, in certain cases even more so than expression^42^. Stabilization of splicing factors in tumors would indeed be a mechanism for aberrant splicing in tumors.

We also identified a great number of exons that showed differential usage specific to subtype. The ER− HER2− tumors showed differential usage of additional exons in mitotic cell cycle genes, as well as genes encoding several subunits of integrin receptor complexes. Integrin signaling is known to mediate several aspects of tumorigenesis including invasion and metastasis^43–45^. While expression of *INTB4* has been shown associate with triple negative tumors^46^, expression of a splice variant of *ITGA6* has been sown to regulate proliferation in colorectal cancer^47^, both of which show alternative exon usage in the ER− HER2− tumors in our analysis. The ER+ HER2− tumors were highly enriched for splicing or alternative expression of exons in genes involved in small GTPase activity such as the Ras and Rho oncogenic signaling pathways. Several guanine nucleotide exchange factors (GEFs) and GTPase activating proteins (GAPs), which regulate the activity of Rho GTPases, were subject to alternative exon usage in this subtype. Deregulation of activity or expression of GEFs and GAPs has been observed in cancer, including breast cancer^48–50^. Alterations in DNA methylation is an early event in cancer progression; changes include both global hypomethylation and hypermethylation of CpGs in gene promoters^51, 52^. Genes functioning in chromatin modification were overrepresented in the HER2+ subtype including two DNA methyltransferase genes, *DNMT3A* and *DNMT3B*, with known implications in cancer^53, 54^.

Among other splicing events identified are previously reported events in genes such as *CD44*, *INSR*, and Tenacin C (*TNC*). *CD44* consists of 2 constitutive regions divided by a highly variable region encoded by 10 exons, and several splice variants have been shown to be involved in tumorigenesis^55, 56^. We see higher FC in 5 and 4 of the variable exons in the ER+ HER2− and HER2+ tumor classes respectively. An isoform of the *INSR gene*, lacking the short exon in position chr19:7150508-7150543, has been previously implicated in breast cancer^57^. The exon defining this isoform was identified with lower relative FC in all three tumor classes compared to normal breast tissue in the TCGA dataset. This particular isoform has been shown to be involved in cell migration, and protection from apoptosis^58^. We also observe previously reported isoforms and events in Tenacin C^59^.

Some of the transcript variants described in this study may have oncogenic potential. For example, the *IQCG* transcript identified in ER- breast cancer contains exons 8–12 of this gene. This exact region of *IQCG* is translocated and fused to the N-terminal of *NUP98* in an acute T-lymphoid/myeloid leukemia^60^, suggesting that deregulated expression of this region may be oncogenic. Tumor Protein D52 (*TPD52*) is a putative oncogene located on 8q21 and involved in vesicular transport^24^. An alternative transcript of the *TPD52* locus called PrLZ (Prostate-leucine zipper) is found in prostate cancer^61^ and is similar to the one we have identified in breast cancer samples. Increased expression and copy number of *TPD52* has been shown, specifically in luminal B cancers^62, 63^. Interestingly, only the variant expressing exon (chr8:81083660-81083836) showed correlation to copy number when the expression of both *TPD52* variants was analyzed by qRT-PCR in the independent patient cohort (data not shown). We have also shown that the intronic variant of *ACOX2* identified in this study associates with better outcome in ER+ patients^64^. Further characterization of the functional role of genes with differential exon usage will be needed to determine the biological role of specific transcript variants.

By analyzing two different datasets, TCGA BRCA and an independent set of 43 samples, differing in both sequencing lengths and depths we were able to identify many exons overlapping in both datasets. Four of the genes with differential exon usage among ER+ HER2− and ER− HER2− patients were validated by qRT-PCR in an independent patient cohort. In addition we validated the differential expression of an intronic start variant of the *ACOX2* gene. Although these exons were not called in the independent dataset, this transcript was identified in the Radium/Rutgers cohort by other methods, including Cuffdiff^64^. There are several validated tools for analysis of alternative splicing, differential isoform, and differential exon usage. While DEXseq is a validated and valuable method for differential exon analysis, it is very consuming (both time and computationally) when analyzing large datasets. In addition, DEXseq is highly sensitive to within-group variance, resulting in few calls for differential exon usage when the groups consist of samples in the 100 range (TCGA), and many more in the smaller dataset. Only three exons on Chr22 were identified as differentially expressed in both the TCGA and Radium/Rutgers cohort. This shows that different analysis methods can have diverging strengths, and complimentary methods such as the one presented here, can be valuable when performing analysis on large datasets. The alternative transcription and splicing events identified in this study add new insight into the biology of breast cancer subclasses.

The analysis presented in this paper was performed few years ago on the small Radium/Rutgers set, then re-analysed on the TCGA dataset, where it became evident that to analyse that large dataset we need a new method. While our paper has been in submission/review and while working on proving the validity of our method, recently another paper was published which performed an analysis similar to our design, but with main focus on basal-like breast tumors^65^. The authors identified ~4500 genes with splicing imbalances between basal-like breast cancer (ER- HER2-) and normal breast samples using exon-arrays. 1082 genes were identified in both our analysis of alternative exon usage between the ER-HER2- and NBS groups and the Gracio *et al*. analysis. This is a significant overlap (p = 3.870623e-10, hypergeometric test), giving further validation to the presented method.

In summary, we have developed a conservative method to analyze differential exon usage in a large dataset of breast cancers, which showed that a great number and variety of splicing and alternative transcription events can be seen in breast cancer, both common to all breast tumors, and events specific to each of three clinical subtypes. The rich and complex biology of this transcriptional and post-transcriptional diversity is currently unknown.

## Methods

### Classification Of TCGA Samples

Focal copy number alteration data (“focal_data_by_genes.txt”) of ERBB2, obtained from Broad GDAC (http://firebrowse.org/?cohort=BRCA), had a bimodal distribution across primary tumors (1097 samples), as shown in Supplementary Figure S2. Tumors in high/low mode of ERBB2 focal copy number data were considered HER2+ and HER2− respectively. RSEM scaled estimates (RNAseqV2) of all genes were also obtained from Broad GDAC, and median of all genes were set to 1 in each tumor to eliminate systematic error. Expression of each gene was calculated as log_2_(1 + 1023 · median adjusted scaled estimate). Expression of ESR1 had a bimodal distribution across tumors, as shown in Supplementary Figure S2. Tumors in high/low mode of ESR1 expression were considered ER+ and ER− respectively. The tumors for which either ESR1 expression or ERBB2 focal copy number data was unavailable were excluded, and the rest of the tumors were classified into 3 groups: ER+ HER2−, ER− HER2−, and HER2+. Only primary tumors of the histology “infiltrating ductal carcinoma” (766 samples), and normal samples (112) from breast cancer patients were considered beyond this point.

### Further preparation of the TCGA Dataset

RNAseqV2 expression data (RPKM of exons) were also obtained from Broad GDAC. The exons were mapped to exons of known genes and lincRNAs using TxDb.Hsapiens.UCSC.hg19.knownGene, and TxDb.Hsapiens.UCSC.hg19.lincRNAsTranscripts packages in R, and the unmapped exons were discarded. Exons with no expression in any sample (0 RPKM in all samples) were filtered. Tumors with unusually low median of all exons were excluded as well, leaving 747 ductal tumor samples in the final analysis (Supplementary Table S3). Systematic error was then eliminated in the same way as above, and expression level was calculated as log_2_(1 + 1023 · median adjusted RPKM) (Supplementary Fig. S4).

60% of the ER+ HER2− samples were randomly selected to create a ER+ HER2− sub-dataset, and the process was repeated 100 times to create 100 ER+ HER2− sub-datasets. 100 ER− HER2− sub-datasets, 100 HER2+ sub-datasets, and 100 normal sub-datasets were created in the same way. In each sub-dataset, we calculated the mode expression of each exon, the value(s) of x where f′(x) = 0 and f″(x) < 0 in the density plots of the expression levels (i.e the most probable value(s) of x). If the distribution was unimodal, or had a single dominant mode (height of the tallest peak/height of the second tallest peak ≥2), then there was no ambiguity; but if the distribution had multiple modes of comparable frequency then the exon was excluded from that particular sub-dataset as invalid (Supplementary Fig. S5). Then, in each trial, we compared one of the 100 ER− HER2− sub-datasets against one of the 100 ER+ HER2− sub-datasets to calculate the log_2_ FC between the 2 classes, and thus there were 10,000 trials of ER− HER2− vs ER+ HER2− comparison. In the same way, we did 10,000 trials for comparison of other pairs of classes. As illustrated in Supplementary Figure S6, in each sub-dataset, a subset of exons had mode expression close to 0. In a trial, if an exon had mode expression close to 0 in both sub-datasets, or invalid mode expression in one or both sub-dataset, then the exon was excluded from that particular trial. In all other cases, log_2_ FC of an exon was calculated as difference in mode expression of that exon in the two sub-datasets. If in a trial a gene had less than 5 exons with valid log_2_ FC, that gene was excluded from that particular trial.

### Analysis of the TCGA Dataset

Consider the log_2_ FC of every exon of a given gene between 2 classes. In the absence of differential splicing, there are three possibilities: (a) log_2_ FC is approximately zero for all exons suggesting the gene is similarly expressed, (b) log_2_ FC is positive but approximately same for all exons suggesting the gene is up-regulated, (c) log_2_ FC is negative but approximately same for all exons suggesting the gene is down-regulated. Under all 3 circumstances, log_2_ FC is approximately same for all exons, so the distribution of log_2_ FC will be unimodal (eg. *TP53BP1* in ER− HER2− vs ER+ HER2−, Fig. 1A). In the presence of differential splicing, while most of the exons will be simply scaled-up or scaled-down or untouched as before, a few exons will receive special treatment, i.e. will have either unusually high or unusually low log_2_ FC compared to rest of the exons of that gene. In both cases, the distribution of log_2_ FC will be multimodal (bimodal in simplest cases): in the former situation a minority of exons will form one or more smaller peaks to the right of the tallest peak, and in the later situation a minority of exons will form one or more smaller peaks to the left of the tallest peak. Most exons will belong to the tallest peak, and we label these exons as 0 to indicate that they did not receive any special treatment. Exons that belong to smaller peaks (if any) to the right/left of the tallest peak are called ±1 respectively, to indicate that they had unusually high or unusually low log_2_ FC, as illustrated in Supplementary Figure S7.

Hence in each trial, we identified the exons with unusually high (called +1) or unusually low (called −1) log_2_ FC, and for each comparison (eg. ER− HER2− vs ER+ HER2−) we scored each exon as (the number of trials in which it got called +1) − (the number of trials in which it got called −1). Finally, in each comparison, we shortlisted the exons with score beyond 3 standard deviation from the mean score of all exons, as shown in Supplementary Figure S8. In each trial, we also recorded which genes went up/down in one class compared to the other; and thus were able to determine which genes went up/down in one class compared to the other, consistently in most of the trials.

### Note

Density plots were made using “bkde” function of R package “KernSmooth” during the classification of TCGA samples, and the preparation of TCGA and validation dataset. However, the base function “density” in R was used for this purpose during the analysis of TCGA and validation dataset.

### Tissue Collection, RNA extraction, and Sequencing of independent dataset

Primary breast tumor biopsies for sample set A and B were collected and sequenced independently. In the case of sample set A, 13 tissue samples from the Cancer Institute of New Jersey (CINJ) in NJ, USA and 16 tissue samples from Radium Hospital in Oslo, Norway^20^ underwent RNA extraction using the Trizol reagent per the manufacturer’s protocol. For sample set B, 24 breast tumors from CINJ underwent RNA extraction, also using the Trizol reagent. Raw sequence data are available from the Sequence Read Archive using accession number SRA057220. We call this the Radium/Rutgers cohort. This dataset also includes an additional 6 “normal breast” tissue samples from mammoplasty procedures collected from Oslo University Hospital. Each sample underwent IHC and FISH assays to determine ER and HER2 status, information provided in Supplementary Table S18.

We followed the standard TruSeq mRNA protocol recommended by Illumina for library generation. Briefly, mRNA was isolation from ~1 ug total RNA by a poly-T bead purification, followed by a shearing step to ~150 bp using Covaris AFA sonication. A complementary DNA (cDNA) library was created for each sample using random hexamer priming. Sequencing adapters were ligated on both ends of the cDNAs. Finally, the cDNA plus adapter library was gel-selected to the appropriate size for sequencing. All samples in set A were sequenced using the Illumina Genome Analyzer IIx at the Mount Sinai School of Medicine (MSSM), while the samples in set B were sequenced at the same location on the Illumina HiSeq 2000.

### Analysis of the Radium/Rutgers dataset

Raw sequencing reads from the independent sample set were aligned to the Human reference genome (Ensemble GRCh 37) using Tophat v1.4.1. The number of mapped reads per sample is included in Supplementary Table S18.

To analyze the data, we mapped the exonic parts, as counted by DEXseq^18^, to exons of known genes and lncRNAs as before, and aggregated the number of reads per million mapped reads for these exonic parts to obtain reads per million values of hg19 exons. Junction reads were counted for each overlapping feature. RPKM values were then calculated for each exon. Expression levels were calculated as log_2_(1 + 127 · median adjusted RPKM), and mode expression of each exon was determined in ER− HER2− and ER+ HER2− group. If the distribution was unimodal, or had a single dominant mode (height of the tallest peak/height of the second tallest peak ≥1.8), then there was no ambiguity; but if the distribution had multiple modes of comparable frequency then the exon was excluded from the analysis. If an exon had mode expression close to 0 in both groups, then that exon was excluded as well. The genes that had less than 5 exons left after these exclusions were excluded as well. As before, for the remaining genes, log_2_ FC of each remaining exon was calculated as difference in mode expression between the two groups, and the exons with unusually high/low log_2_ FC were called ±1 respectively. Expression level was calculated as log_2_(1 + 127 · median adjusted RPKM), mode expression of each exon was determined in ER− HER2− and ER+ HER2− group, and analysis was performed as described above for the TCGA dataset.

### qRT-PCR Validation

40 breast tumor tissue samples from the MicMa cohort^20^ were obtained for Taqman qRT-PCR validation of variant exon expression. Each sample underwent IHC and FISH assays to determine ER and HER2 status; in total 20 ER− HER2− samples and 20 ER+ HER2− samples were used for statistical validation. These samples underwent RNA extraction using the Trizol reagent per the manufacturer’s protocol, followed by gDNA removal using Ambion® DNA-free™ DNase Treatment and Removal Reagents (AM1906, Applied Biosystems). cDNA was synthesized using Life technologies (Applied Biosystems), High Capacity cDNA Reverse transcriptase kit. Taqman gene expression assays, either pre-designed or custom made, were used in qRT-PCR reactions with the TaqMan 2x Expression Master Mix and read on the Applied Biosystems 7900HT Fast Real-Time PCR System with FAM as the reporter dye. Mean Ct values from PMM1 and RPL32 were used as endogenous control. Samples were analyzed in duplicates, and all samples were normalized to FirstChoice® Human Breast Total RNA (Ambion). A no-RT sample was added as a control for genomic DNA contamination as some probes did not span exon-exon boundaries due to length restrictions. Probe efficiency was assayed for all pairs using a standard dilution curve, and relative expression levels were calculated using the method suggested in ref. 66. For log_2_ calculation of relative expression a pseudo-value of 1 was added to all relative expression values, and association to ER status was assed using the Wilcoxon’s rank-sum test. All Taqman probes used are listed in Supplementary Table S13.

### DEXseq

DEXSeq Bioconductor (Release 3.4)^18^ was used for the DEXseq analysis of the ER+ HER2− and ER− HER2− samples from both previously described datasets. dexseq_prepare_annotation.py was used to generate the non-overlapping exon reference file, and dexseq_count.py was used to count reads at each exon using.bam alignment files (Radium/Rutgers dataset only). Due to computation time analysis of the TCGA dataset was limited to the validated genes (*MYO6, EPB41L1, TPD52, IQCG*, and *ACOX2*) and all genes on Chr22. Analysis of genes on Chr22 only was also performed for the Radium/Rutgers dataset for comparison. Default parameters described in the DEXSeq vignettes on Bioconductor were implemented.

### Ethics statement

All of the human samples were used in accordance with approved guidelines from Oslo University Hospital and the Rutgers Cancer Institute of New Jersey. Use of the samples from Oslo University Hospital was approved by the Norwegian Regional Committee (REC) for Medical and Health Research Ethics (REC South East, reference numbers S97103 and 429-04148), all patients were informed and have declared written informed consent that their samples are used for research. Samples from Rutgers Cancer Institute of New Jersey were de-identified patient samples collected under a tissue banking protocol and approved for use in this study by The Rutgers Health Sciences New Brunswick/Piscataway Institutional Review Board, number 0220080121. Individual patient consent for the use of these patient samples was not required.

### Data availability

Raw sequence data are available from the Sequence Read Archive using accession number SRA057220.

## Electronic supplementary material

Supplementary File 1

Dataset S10

Dataset S15

Dataset S16

Dataset S17
