# Supplementary material for: Widespread alternative exon usage in clinically distinct subtypes of Invasive Ductal Carcinoma
Source: Sci Rep. 2017 Jul 17;7:5568. doi: 10.1038/s41598-017-05537-0 (PMC5514065; doi:10.1038/s41598-017-05537-0)
Supplement: Supplementary file 1 [file 41598_2017_5537_MOESM1_ESM.pdf]

Supplementary File 1

Widespread alternative exon usage in Clinically distinct subtypes of Invasive Ductal Carcinoma

Sunniva Stordal Bjørklund[1,2,3,+], Anshuman Panda[1,5,+], Surendra Kumar[2,3,4], Michael Seiler[1,7], Doug Robinson[7], Jinesh Gheeya[1], Yao Ming[1], Grethe I Grenaker Alnæs[2], Deborah Toppmeyer[1], Margit Riis[4,8,9], Bjørn Naume[10], Anne-Lise Børresen-Dale[2,3], Vessela N. Kristensen[2,3,4], Shridar Ganesan[1,*] , Gyan Bhanot [1,5,6,*]

[*] Corresponding authors

Email addresses:

gyanbhanot@gmail.com, ganesash@cinj.rutgers.edu.

Supplementary Figure S1

Method overview

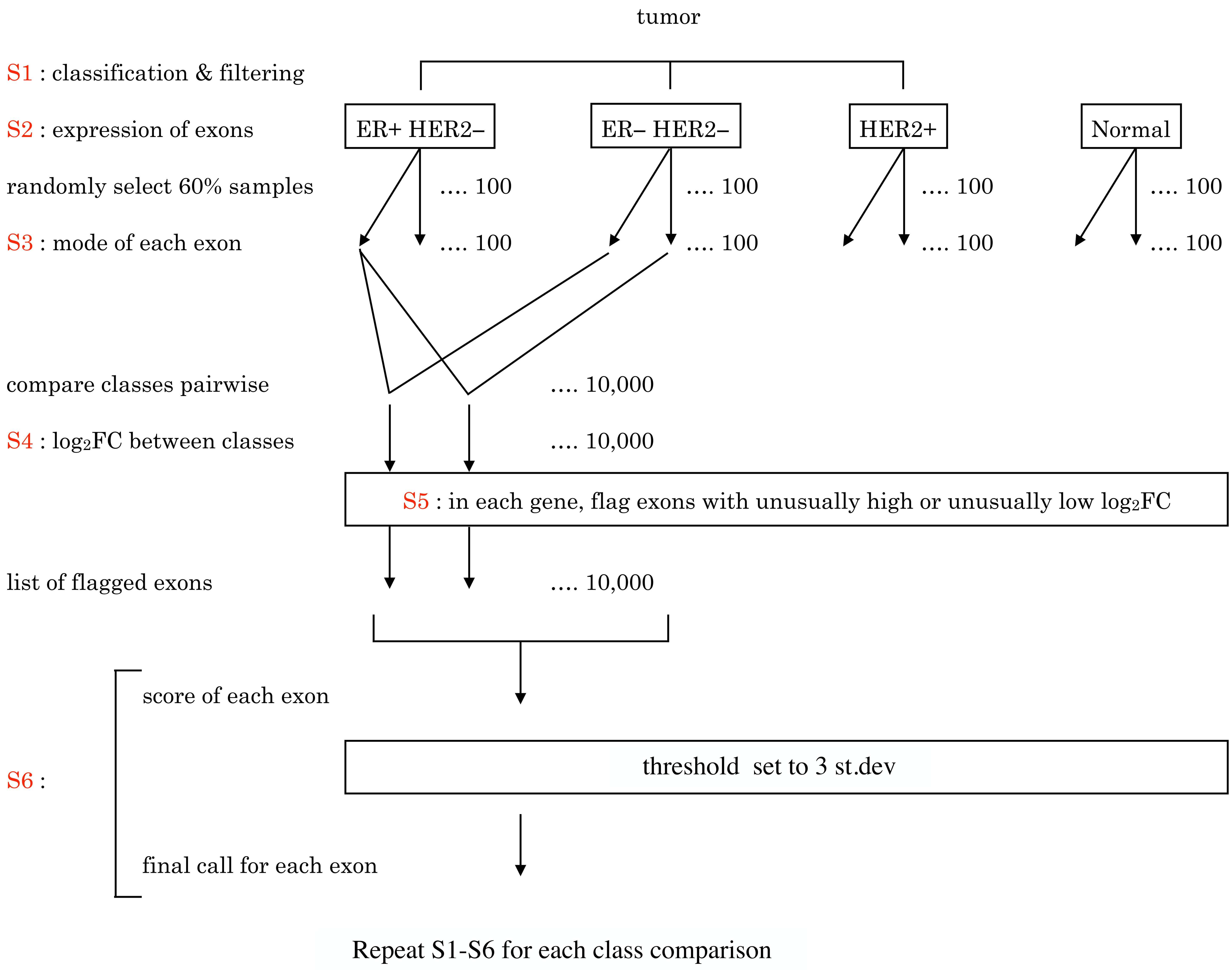

Supplementary Figure S1. *Flowchart of method used to determine differential exon usage between two biological classes.* Details for each step follow in Supplementary Figures S4-S8.

Supplementary Figure S2

Classification and filtering of TCGA breast cancer samples

S1 : Classification of Breast Cancer function : bkde R package : KernSmooth

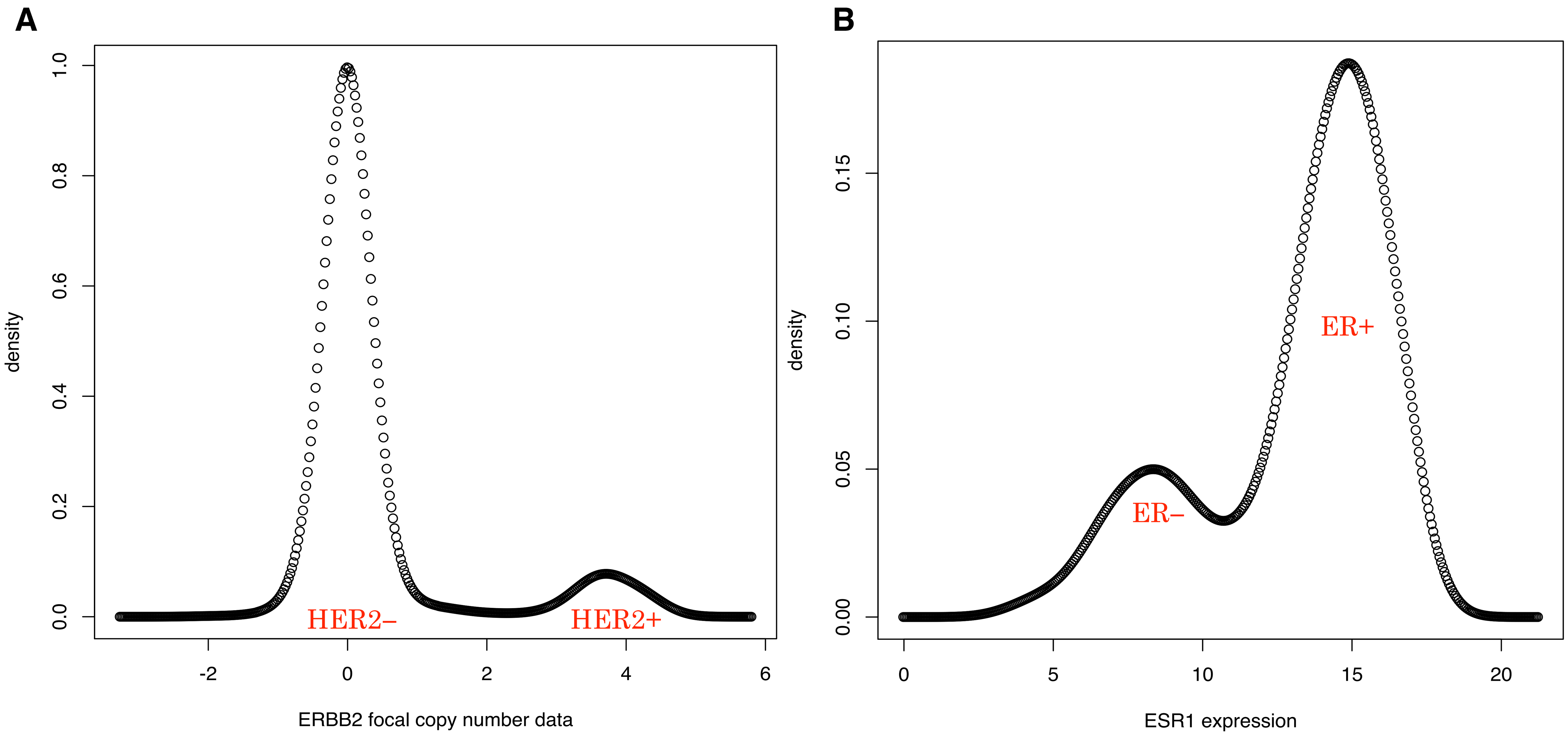

C

ER	HER2	Class	n
+	+	HER2+	54
–	+	HER2+	43
+	–	ER+ HER2–	493
–	–	ER– HER2–	157

Supplementary Figure S2. *Classification and filtering, determination of HER2 and ER status.* Focal copy number data for ERBB2 for all breast cancer tumor samples in the TCGA cohort were plotted and showed a clear bimodal distribution (A). Tumor samples with ERBB2 focal copy number >2 were classified as HER2+. ESR1 expression values (median adjusted and log_2 normalized) were used to determine ER status for all the tumor samples in the cohort (B). Samples to the right of the local minimum were considered ER+, and the tumor samples to the left of the local minimum were considered ER-. Only samples labeled as "Ductal" in the clinical file were considered after this point, leaving n= 97 HER2+ samples, n= 493 ER+ HER2-, and n= 157 ER- HER2- samples for the continuation of the analysis (C).

Supplementary Table S3- TCGA sample classification

Sample	Class
TCGA-3C-AALJ-01	ER+ HER2−
TCGA-A1-A0SD-01	ER+ HER2−
TCGA-A1-A0SF-01	ER+ HER2−
TCGA-A1-A0SH-01	ER+ HER2−
TCGA-A1-A0SI-01	ER+ HER2−
TCGA-A1-A0SJ-01	ER+ HER2−
TCGA-A1-A0SQ-01	ER+ HER2−
TCGA-A2-A04N-01	ER+ HER2−
TCGA-A2-A04P-01	ER+ HER2−
TCGA-A2-A04R-01	ER+ HER2−
TCGA-A2-A04V-01	ER+ HER2−
TCGA-A2-A04Y-01	ER+ HER2−
TCGA-A2-A0CL-01	ER+ HER2−
TCGA-A2-A0CP-01	ER+ HER2−
TCGA-A2-A0CQ-01	ER+ HER2−
TCGA-A2-A0CS-01	ER+ HER2−
TCGA-A2-A0CT-01	ER+ HER2−
TCGA-A2-A0CU-01	ER+ HER2−
TCGA-A2-A0CV-01	ER+ HER2−
TCGA-A2-A0CW-01	ER+ HER2−
TCGA-A2-A0D3-01	ER+ HER2−
TCGA-A2-A0D4-01	ER+ HER2−
TCGA-A2-A0EM-01	ER+ HER2−
TCGA-A2-A0EO-01	ER+ HER2−
TCGA-A2-A0ER-01	ER+ HER2−
TCGA-A2-A0ET-01	ER+ HER2−
TCGA-A2-A0EU-01	ER+ HER2−
TCGA-A2-A0EV-01	ER+ HER2−
TCGA-A2-A0ST-01	ER+ HER2−
TCGA-A2-A0SU-01	ER+ HER2−
TCGA-A2-A0SV-01	ER+ HER2−
TCGA-A2-A0SW-01	ER+ HER2−
TCGA-A2-A0T3-01	ER+ HER2−
TCGA-A2-A0T5-01	ER+ HER2−
TCGA-A2-A0T7-01	ER+ HER2−
TCGA-A2-A0YC-01	ER+ HER2−
TCGA-A2-A0YF-01	ER+ HER2−
TCGA-A2-A0YH-01	ER+ HER2−
TCGA-A2-A0YI-01	ER+ HER2−
TCGA-A2-A0YT-01	ER+ HER2−
TCGA-A2-A1FW-01	ER+ HER2−
TCGA-A2-A1FX-01	ER+ HER2−
TCGA-A2-A1FZ-01	ER+ HER2−

TCGA-A2-A1G4-01	ER+ HER2−
TCGA-A2-A1G6-01	ER+ HER2−
TCGA-A2-A259-01	ER+ HER2−
TCGA-A2-A25B-01	ER+ HER2−
TCGA-A2-A25C-01	ER+ HER2−
TCGA-A2-A25E-01	ER+ HER2−
TCGA-A2-A3XT-01	ER+ HER2−
TCGA-A2-A3XW-01	ER+ HER2−
TCGA-A2-A3Y0-01	ER+ HER2−
TCGA-A2-A4S3-01	ER+ HER2−
TCGA-A7-A0CD-01	ER+ HER2−
TCGA-A7-A0CE-01	ER+ HER2−
TCGA-A7-A0CH-01	ER+ HER2−
TCGA-A7-A0CJ-01	ER+ HER2−
TCGA-A7-A0D9-01	ER+ HER2−
TCGA-A7-A0DB-01	ER+ HER2−
TCGA-A7-A13F-01	ER+ HER2−
TCGA-A7-A13G-01	ER+ HER2−
TCGA-A7-A26H-01	ER+ HER2−
TCGA-A7-A26J-01	ER+ HER2−
TCGA-A7-A5ZW-01	ER+ HER2−
TCGA-A7-A6VX-01	ER+ HER2−
TCGA-A8-A06N-01	ER+ HER2−
TCGA-A8-A06O-01	ER+ HER2−
TCGA-A8-A06P-01	ER+ HER2−
TCGA-A8-A06Q-01	ER+ HER2−
TCGA-A8-A06R-01	ER+ HER2−
TCGA-A8-A06T-01	ER+ HER2−
TCGA-A8-A06U-01	ER+ HER2−
TCGA-A8-A06X-01	ER+ HER2−
TCGA-A8-A06Y-01	ER+ HER2−
TCGA-A8-A06Z-01	ER+ HER2−
TCGA-A8-A075-01	ER+ HER2−
TCGA-A8-A079-01	ER+ HER2−
TCGA-A8-A07E-01	ER+ HER2−
TCGA-A8-A07F-01	ER+ HER2−
TCGA-A8-A07G-01	ER+ HER2−
TCGA-A8-A07J-01	ER+ HER2−
TCGA-A8-A07L-01	ER+ HER2−
TCGA-A8-A07P-01	ER+ HER2−
TCGA-A8-A07W-01	ER+ HER2−
TCGA-A8-A07Z-01	ER+ HER2−
TCGA-A8-A081-01	ER+ HER2−
TCGA-A8-A082-01	ER+ HER2−
TCGA-A8-A083-01	ER+ HER2−
TCGA-A8-A084-01	ER+ HER2−

TCGA-A8-A085-01	ER+ HER2−
TCGA-A8-A086-01	ER+ HER2−
TCGA-A8-A08A-01	ER+ HER2−
TCGA-A8-A08C-01	ER+ HER2−
TCGA-A8-A08F-01	ER+ HER2−
TCGA-A8-A08G-01	ER+ HER2−
TCGA-A8-A08H-01	ER+ HER2−
TCGA-A8-A08I-01	ER+ HER2−
TCGA-A8-A08L-01	ER+ HER2−
TCGA-A8-A08O-01	ER+ HER2−
TCGA-A8-A08P-01	ER+ HER2−
TCGA-A8-A08T-01	ER+ HER2−
TCGA-A8-A08Z-01	ER+ HER2−
TCGA-A8-A090-01	ER+ HER2−
TCGA-A8-A091-01	ER+ HER2−
TCGA-A8-A092-01	ER+ HER2−
TCGA-A8-A093-01	ER+ HER2−
TCGA-A8-A094-01	ER+ HER2−
TCGA-A8-A095-01	ER+ HER2−
TCGA-A8-A096-01	ER+ HER2−
TCGA-A8-A097-01	ER+ HER2−
TCGA-A8-A099-01	ER+ HER2−
TCGA-A8-A09A-01	ER+ HER2−
TCGA-A8-A09B-01	ER+ HER2−
TCGA-A8-A09C-01	ER+ HER2−
TCGA-A8-A09D-01	ER+ HER2−
TCGA-A8-A09E-01	ER+ HER2−
TCGA-A8-A09K-01	ER+ HER2−
TCGA-A8-A09M-01	ER+ HER2−
TCGA-A8-A09N-01	ER+ HER2−
TCGA-A8-A09Q-01	ER+ HER2−
TCGA-A8-A09R-01	ER+ HER2−
TCGA-A8-A0A9-01	ER+ HER2−
TCGA-AC-A23E-01	ER+ HER2−
TCGA-AC-A2BM-01	ER+ HER2−
TCGA-AC-A5XU-01	ER+ HER2−
TCGA-AC-A62V-01	ER+ HER2−
TCGA-AC-A6NO-01	ER+ HER2−
TCGA-AC-A7VB-01	ER+ HER2−
TCGA-AC-A8OP-01	ER+ HER2−
TCGA-AN-A03X-01	ER+ HER2−
TCGA-AN-A03Y-01	ER+ HER2−
TCGA-AN-A041-01	ER+ HER2−
TCGA-AN-A046-01	ER+ HER2−
TCGA-AN-A049-01	ER+ HER2−
TCGA-AN-A04A-01	ER+ HER2−

TCGA-AN-A0AJ-01	ER+ HER2−
TCGA-AN-A0AK-01	ER+ HER2−
TCGA-AN-A0AM-01	ER+ HER2−
TCGA-AN-A0AS-01	ER+ HER2−
TCGA-AN-A0FD-01	ER+ HER2−
TCGA-AN-A0FF-01	ER+ HER2−
TCGA-AN-A0FK-01	ER+ HER2−
TCGA-AN-A0FW-01	ER+ HER2−
TCGA-AN-A0FY-01	ER+ HER2−
TCGA-AN-A0FZ-01	ER+ HER2−
TCGA-AN-A0XL-01	ER+ HER2−
TCGA-AN-A0XN-01	ER+ HER2−
TCGA-AN-A0XO-01	ER+ HER2−
TCGA-AN-A0XP-01	ER+ HER2−
TCGA-AN-A0XR-01	ER+ HER2−
TCGA-AN-A0XS-01	ER+ HER2−
TCGA-AN-A0XT-01	ER+ HER2−
TCGA-AN-A0XU-01	ER+ HER2−
TCGA-AN-A0XV-01	ER+ HER2−
TCGA-AN-A0XW-01	ER+ HER2−
TCGA-AO-A03L-01	ER+ HER2−
TCGA-AO-A03M-01	ER+ HER2−
TCGA-AO-A03O-01	ER+ HER2−
TCGA-AO-A03P-01	ER+ HER2−
TCGA-AO-A03R-01	ER+ HER2−
TCGA-AO-A03T-01	ER+ HER2−
TCGA-AO-A03V-01	ER+ HER2−
TCGA-AO-A0JA-01	ER+ HER2−
TCGA-AO-A0JC-01	ER+ HER2−
TCGA-AO-A0JD-01	ER+ HER2−
TCGA-AO-A0JF-01	ER+ HER2−
TCGA-AO-A0JI-01	ER+ HER2−
TCGA-AO-A126-01	ER+ HER2−
TCGA-AO-A12A-01	ER+ HER2−
TCGA-AO-A12B-01	ER+ HER2−
TCGA-AO-A1KP-01	ER+ HER2−
TCGA-AO-A1KQ-01	ER+ HER2−
TCGA-AO-A1KT-01	ER+ HER2−
TCGA-AQ-A04L-01	ER+ HER2−
TCGA-AQ-A1H2-01	ER+ HER2−
TCGA-AQ-A1H3-01	ER+ HER2−
TCGA-AR-A0TR-01	ER+ HER2−
TCGA-AR-A0TT-01	ER+ HER2−
TCGA-AR-A0TV-01	ER+ HER2−
TCGA-AR-A0TW-01	ER+ HER2−
TCGA-AR-A0TY-01	ER+ HER2−

TCGA-AR-A0TZ-01	ER+ HER2−
TCGA-AR-A0U2-01	ER+ HER2−
TCGA-AR-A0U3-01	ER+ HER2−
TCGA-AR-A1AN-01	ER+ HER2−
TCGA-AR-A1AP-01	ER+ HER2−
TCGA-AR-A1AS-01	ER+ HER2−
TCGA-AR-A1AU-01	ER+ HER2−
TCGA-AR-A1AV-01	ER+ HER2−
TCGA-AR-A1AW-01	ER+ HER2−
TCGA-AR-A1AX-01	ER+ HER2−
TCGA-AR-A24K-01	ER+ HER2−
TCGA-AR-A24L-01	ER+ HER2−
TCGA-AR-A24N-01	ER+ HER2−
TCGA-AR-A24P-01	ER+ HER2−
TCGA-AR-A24R-01	ER+ HER2−
TCGA-AR-A24S-01	ER+ HER2−
TCGA-AR-A24V-01	ER+ HER2−
TCGA-AR-A24W-01	ER+ HER2−
TCGA-AR-A24Z-01	ER+ HER2−
TCGA-AR-A252-01	ER+ HER2−
TCGA-AR-A255-01	ER+ HER2−
TCGA-B6-A0I2-01	ER+ HER2−
TCGA-B6-A0I5-01	ER+ HER2−
TCGA-B6-A0IB-01	ER+ HER2−
TCGA-B6-A0IK-01	ER+ HER2−
TCGA-B6-A0IO-01	ER+ HER2−
TCGA-B6-A0RG-01	ER+ HER2−
TCGA-B6-A0RI-01	ER+ HER2−
TCGA-B6-A0RL-01	ER+ HER2−
TCGA-B6-A0RM-01	ER+ HER2−
TCGA-B6-A0RN-01	ER+ HER2−
TCGA-B6-A0RO-01	ER+ HER2−
TCGA-B6-A0WS-01	ER+ HER2−
TCGA-B6-A0WT-01	ER+ HER2−
TCGA-B6-A0WV-01	ER+ HER2−
TCGA-B6-A0WW-01	ER+ HER2−
TCGA-B6-A0WY-01	ER+ HER2−
TCGA-B6-A0WZ-01	ER+ HER2−
TCGA-B6-A0X1-01	ER+ HER2−
TCGA-B6-A0X4-01	ER+ HER2−
TCGA-B6-A0X5-01	ER+ HER2−
TCGA-B6-A1KC-01	ER+ HER2−
TCGA-B6-A1KI-01	ER+ HER2−
TCGA-B6-A401-01	ER+ HER2−
TCGA-BH-A0AU-01	ER+ HER2−
TCGA-BH-A0AY-01	ER+ HER2−

TCGA-BH-A0AZ-01	ER+ HER2−
TCGA-BH-A0B0-01	ER+ HER2−
TCGA-BH-A0B4-01	ER+ HER2−
TCGA-BH-A0B6-01	ER+ HER2−
TCGA-BH-A0BC-01	ER+ HER2−
TCGA-BH-A0BD-01	ER+ HER2−
TCGA-BH-A0BF-01	ER+ HER2−
TCGA-BH-A0BJ-01	ER+ HER2−
TCGA-BH-A0BM-01	ER+ HER2−
TCGA-BH-A0BO-01	ER+ HER2−
TCGA-BH-A0BP-01	ER+ HER2−
TCGA-BH-A0BQ-01	ER+ HER2−
TCGA-BH-A0BR-01	ER+ HER2−
TCGA-BH-A0BS-01	ER+ HER2−
TCGA-BH-A0BT-01	ER+ HER2−
TCGA-BH-A0BV-01	ER+ HER2−
TCGA-BH-A0BZ-01	ER+ HER2−
TCGA-BH-A0C0-01	ER+ HER2−
TCGA-BH-A0C7-01	ER+ HER2−
TCGA-BH-A0DE-01	ER+ HER2−
TCGA-BH-A0DG-01	ER+ HER2−
TCGA-BH-A0DH-01	ER+ HER2−
TCGA-BH-A0DI-01	ER+ HER2−
TCGA-BH-A0DK-01	ER+ HER2−
TCGA-BH-A0DO-01	ER+ HER2−
TCGA-BH-A0DQ-01	ER+ HER2−
TCGA-BH-A0DS-01	ER+ HER2−
TCGA-BH-A0DT-01	ER+ HER2−
TCGA-BH-A0DV-01	ER+ HER2−
TCGA-BH-A0DX-01	ER+ HER2−
TCGA-BH-A0E1-01	ER+ HER2−
TCGA-BH-A0E2-01	ER+ HER2−
TCGA-BH-A0E7-01	ER+ HER2−
TCGA-BH-A0EA-01	ER+ HER2−
TCGA-BH-A0EB-01	ER+ HER2−
TCGA-BH-A0EI-01	ER+ HER2−
TCGA-BH-A0GY-01	ER+ HER2−
TCGA-BH-A0GZ-01	ER+ HER2−
TCGA-BH-A0H0-01	ER+ HER2−
TCGA-BH-A0H3-01	ER+ HER2−
TCGA-BH-A0H5-01	ER+ HER2−
TCGA-BH-A0H6-01	ER+ HER2−
TCGA-BH-A0H7-01	ER+ HER2−
TCGA-BH-A0H9-01	ER+ HER2−
TCGA-BH-A0HA-01	ER+ HER2−
TCGA-BH-A0HB-01	ER+ HER2−

TCGA-BH-A0HI-01	ER+ HER2−
TCGA-BH-A0HK-01	ER+ HER2−
TCGA-BH-A0HO-01	ER+ HER2−
TCGA-BH-A0HQ-01	ER+ HER2−
TCGA-BH-A0HU-01	ER+ HER2−
TCGA-BH-A0HW-01	ER+ HER2−
TCGA-BH-A0HX-01	ER+ HER2−
TCGA-BH-A0RX-01	ER+ HER2−
TCGA-BH-A0W3-01	ER+ HER2−
TCGA-BH-A0W4-01	ER+ HER2−
TCGA-BH-A0W5-01	ER+ HER2−
TCGA-BH-A0W7-01	ER+ HER2−
TCGA-BH-A18F-01	ER+ HER2−
TCGA-BH-A18H-01	ER+ HER2−
TCGA-BH-A18I-01	ER+ HER2−
TCGA-BH-A18J-01	ER+ HER2−
TCGA-BH-A18K-01	ER+ HER2−
TCGA-BH-A18L-01	ER+ HER2−
TCGA-BH-A18M-01	ER+ HER2−
TCGA-BH-A18N-01	ER+ HER2−
TCGA-BH-A18P-01	ER+ HER2−
TCGA-BH-A18Q-01	ER+ HER2−
TCGA-BH-A1ES-01	ER+ HER2−
TCGA-BH-A1ET-01	ER+ HER2−
TCGA-BH-A1EU-01	ER+ HER2−
TCGA-BH-A1EW-01	ER+ HER2−
TCGA-BH-A1EX-01	ER+ HER2−
TCGA-BH-A1EY-01	ER+ HER2−
TCGA-BH-A1F5-01	ER+ HER2−
TCGA-BH-A1F8-01	ER+ HER2−
TCGA-BH-A1FB-01	ER+ HER2−
TCGA-BH-A1FD-01	ER+ HER2−
TCGA-BH-A1FE-01	ER+ HER2−
TCGA-BH-A1FG-01	ER+ HER2−
TCGA-BH-A1FH-01	ER+ HER2−
TCGA-BH-A1FJ-01	ER+ HER2−
TCGA-BH-A1FL-01	ER+ HER2−
TCGA-BH-A1FM-01	ER+ HER2−
TCGA-BH-A1FN-01	ER+ HER2−
TCGA-BH-A201-01	ER+ HER2−
TCGA-BH-A204-01	ER+ HER2−
TCGA-BH-A42V-01	ER+ HER2−
TCGA-BH-A5IZ-01	ER+ HER2−
TCGA-BH-A5J0-01	ER+ HER2−
TCGA-BH-A6R8-01	ER+ HER2−
TCGA-C8-A12K-01	ER+ HER2−

TCGA-C8-A12M-01	ER+ HER2−
TCGA-C8-A12N-01	ER+ HER2−
TCGA-C8-A12O-01	ER+ HER2−
TCGA-C8-A12U-01	ER+ HER2−
TCGA-C8-A12W-01	ER+ HER2−
TCGA-C8-A12Y-01	ER+ HER2−
TCGA-C8-A130-01	ER+ HER2−
TCGA-C8-A138-01	ER+ HER2−
TCGA-C8-A1HE-01	ER+ HER2−
TCGA-C8-A1HG-01	ER+ HER2−
TCGA-C8-A1HI-01	ER+ HER2−
TCGA-C8-A1HM-01	ER+ HER2−
TCGA-C8-A1HN-01	ER+ HER2−
TCGA-C8-A1HO-01	ER+ HER2−
TCGA-C8-A26V-01	ER+ HER2−
TCGA-C8-A26Z-01	ER+ HER2−
TCGA-C8-A273-01	ER+ HER2−
TCGA-C8-A274-01	ER+ HER2−
TCGA-C8-A27A-01	ER+ HER2−
TCGA-D8-A13Y-01	ER+ HER2−
TCGA-D8-A140-01	ER+ HER2−
TCGA-D8-A141-01	ER+ HER2−
TCGA-D8-A145-01	ER+ HER2−
TCGA-D8-A146-01	ER+ HER2−
TCGA-D8-A1J8-01	ER+ HER2−
TCGA-D8-A1JB-01	ER+ HER2−
TCGA-D8-A1JC-01	ER+ HER2−
TCGA-D8-A1JD-01	ER+ HER2−
TCGA-D8-A1JE-01	ER+ HER2−
TCGA-D8-A1JH-01	ER+ HER2−
TCGA-D8-A1JI-01	ER+ HER2−
TCGA-D8-A1JJ-01	ER+ HER2−
TCGA-D8-A1JM-01	ER+ HER2−
TCGA-D8-A1JP-01	ER+ HER2−
TCGA-D8-A1JU-01	ER+ HER2−
TCGA-D8-A1X6-01	ER+ HER2−
TCGA-D8-A1X9-01	ER+ HER2−
TCGA-D8-A1XA-01	ER+ HER2−
TCGA-D8-A1XB-01	ER+ HER2−
TCGA-D8-A1XD-01	ER+ HER2−
TCGA-D8-A1XF-01	ER+ HER2−
TCGA-D8-A1XG-01	ER+ HER2−
TCGA-D8-A1XL-01	ER+ HER2−
TCGA-D8-A1XM-01	ER+ HER2−
TCGA-D8-A1XR-01	ER+ HER2−
TCGA-D8-A1XU-01	ER+ HER2−

TCGA-D8-A1XY-01	ER+ HER2−
TCGA-D8-A1XZ-01	ER+ HER2−
TCGA-D8-A1Y0-01	ER+ HER2−
TCGA-D8-A1Y1-01	ER+ HER2−
TCGA-D8-A1Y2-01	ER+ HER2−
TCGA-D8-A1Y3-01	ER+ HER2−
TCGA-D8-A27K-01	ER+ HER2−
TCGA-D8-A27L-01	ER+ HER2−
TCGA-D8-A27P-01	ER+ HER2−
TCGA-D8-A27R-01	ER+ HER2−
TCGA-E2-A105-01	ER+ HER2−
TCGA-E2-A108-01	ER+ HER2−
TCGA-E2-A109-01	ER+ HER2−
TCGA-E2-A10A-01	ER+ HER2−
TCGA-E2-A10B-01	ER+ HER2−
TCGA-E2-A10C-01	ER+ HER2−
TCGA-E2-A10E-01	ER+ HER2−
TCGA-E2-A14O-01	ER+ HER2−
TCGA-E2-A14Q-01	ER+ HER2−
TCGA-E2-A14S-01	ER+ HER2−
TCGA-E2-A14T-01	ER+ HER2−
TCGA-E2-A14W-01	ER+ HER2−
TCGA-E2-A14Z-01	ER+ HER2−
TCGA-E2-A153-01	ER+ HER2−
TCGA-E2-A154-01	ER+ HER2−
TCGA-E2-A155-01	ER+ HER2−
TCGA-E2-A156-01	ER+ HER2−
TCGA-E2-A15A-01	ER+ HER2−
TCGA-E2-A15C-01	ER+ HER2−
TCGA-E2-A15D-01	ER+ HER2−
TCGA-E2-A15E-01	ER+ HER2−
TCGA-E2-A15F-01	ER+ HER2−
TCGA-E2-A15I-01	ER+ HER2−
TCGA-E2-A15J-01	ER+ HER2−
TCGA-E2-A15K-01	ER+ HER2−
TCGA-E2-A15O-01	ER+ HER2−
TCGA-E2-A15P-01	ER+ HER2−
TCGA-E2-A15R-01	ER+ HER2−
TCGA-E2-A15S-01	ER+ HER2−
TCGA-E2-A15T-01	ER+ HER2−
TCGA-E2-A1B1-01	ER+ HER2−
TCGA-E2-A1B4-01	ER+ HER2−
TCGA-E2-A1BC-01	ER+ HER2−
TCGA-E2-A1BD-01	ER+ HER2−
TCGA-E2-A1IE-01	ER+ HER2−
TCGA-E2-A1IF-01	ER+ HER2−

TCGA-E2-A1IG-01	ER+ HER2−
TCGA-E2-A1IK-01	ER+ HER2−
TCGA-E2-A1IN-01	ER+ HER2−
TCGA-E2-A1IO-01	ER+ HER2−
TCGA-E2-A1IU-01	ER+ HER2−
TCGA-E2-A1L6-01	ER+ HER2−
TCGA-E2-A1L7-01	ER+ HER2−
TCGA-E2-A1L9-01	ER+ HER2−
TCGA-E2-A1LA-01	ER+ HER2−
TCGA-E2-A56Z-01	ER+ HER2−
TCGA-E2-A570-01	ER+ HER2−
TCGA-E9-A1NE-01	ER+ HER2−
TCGA-E9-A1NG-01	ER+ HER2−
TCGA-E9-A1NH-01	ER+ HER2−
TCGA-E9-A1QZ-01	ER+ HER2−
TCGA-E9-A1R0-01	ER+ HER2−
TCGA-E9-A1R2-01	ER+ HER2−
TCGA-E9-A1R3-01	ER+ HER2−
TCGA-E9-A1R4-01	ER+ HER2−
TCGA-E9-A1R5-01	ER+ HER2−
TCGA-E9-A1R6-01	ER+ HER2−
TCGA-E9-A1R7-01	ER+ HER2−
TCGA-E9-A1RA-01	ER+ HER2−
TCGA-E9-A1RB-01	ER+ HER2−
TCGA-E9-A1RD-01	ER+ HER2−
TCGA-E9-A1RE-01	ER+ HER2−
TCGA-E9-A1RF-01	ER+ HER2−
TCGA-E9-A1RG-01	ER+ HER2−
TCGA-E9-A226-01	ER+ HER2−
TCGA-E9-A227-01	ER+ HER2−
TCGA-E9-A229-01	ER+ HER2−
TCGA-E9-A22A-01	ER+ HER2−
TCGA-E9-A22E-01	ER+ HER2−
TCGA-E9-A22H-01	ER+ HER2−
TCGA-E9-A245-01	ER+ HER2−
TCGA-E9-A247-01	ER+ HER2−
TCGA-E9-A249-01	ER+ HER2−
TCGA-E9-A24A-01	ER+ HER2−
TCGA-EW-A1IY-01	ER+ HER2−
TCGA-EW-A1IZ-01	ER+ HER2−
TCGA-EW-A1J1-01	ER+ HER2−
TCGA-EW-A1J6-01	ER+ HER2−
TCGA-EW-A1OY-01	ER+ HER2−
TCGA-EW-A1P1-01	ER+ HER2−
TCGA-EW-A1P3-01	ER+ HER2−
TCGA-EW-A1P5-01	ER+ HER2−

TCGA-EW-A1P6-01	ER+ HER2−
TCGA-EW-A1PA-01	ER+ HER2−
TCGA-EW-A1PC-01	ER+ HER2−
TCGA-EW-A1PD-01	ER+ HER2−
TCGA-EW-A1PE-01	ER+ HER2−
TCGA-EW-A1PF-01	ER+ HER2−
TCGA-EW-A2FS-01	ER+ HER2−
TCGA-EW-A6SA-01	ER+ HER2−
TCGA-GI-A2C8-01	ER+ HER2−
TCGA-GM-A2D9-01	ER+ HER2−
TCGA-GM-A2DC-01	ER+ HER2−
TCGA-GM-A2DH-01	ER+ HER2−
TCGA-GM-A2DI-01	ER+ HER2−
TCGA-GM-A2DK-01	ER+ HER2−
TCGA-GM-A2DL-01	ER+ HER2−
TCGA-GM-A2DM-01	ER+ HER2−
TCGA-GM-A2DN-01	ER+ HER2−
TCGA-LD-A7W5-01	ER+ HER2−
TCGA-LL-A442-01	ER+ HER2−
TCGA-LL-A5YN-01	ER+ HER2−
TCGA-LL-A73Z-01	ER+ HER2−
TCGA-LL-A7T0-01	ER+ HER2−
TCGA-OL-A5D8-01	ER+ HER2−
TCGA-OL-A5RU-01	ER+ HER2−
TCGA-OL-A5RV-01	ER+ HER2−
TCGA-OL-A5RX-01	ER+ HER2−
TCGA-OL-A66O-01	ER+ HER2−
TCGA-OL-A6VR-01	ER+ HER2−
TCGA-PL-A8LX-01	ER+ HER2−
TCGA-PL-A8LY-01	ER+ HER2−
TCGA-S3-A6ZF-01	ER+ HER2−
TCGA-S3-A6ZH-01	ER+ HER2−
TCGA-S3-AA0Z-01	ER+ HER2−
TCGA-S3-AA11-01	ER+ HER2−
TCGA-S3-AA12-01	ER+ HER2−
TCGA-S3-AA17-01	ER+ HER2−
TCGA-A1-A0SO-01	ER− HER2−
TCGA-A1-A0SP-01	ER− HER2−
TCGA-A2-A04Q-01	ER− HER2−
TCGA-A2-A04T-01	ER− HER2−
TCGA-A2-A04U-01	ER− HER2−
TCGA-A2-A0CM-01	ER− HER2−
TCGA-A2-A0D0-01	ER− HER2−
TCGA-A2-A0D2-01	ER− HER2−
TCGA-A2-A0EQ-01	ER− HER2−
TCGA-A2-A0SX-01	ER− HER2−

TCGA-A2-A0T0-01	ER– HER2–
TCGA-A2-A0T2-01	ER– HER2–
TCGA-A2-A0YE-01	ER– HER2–
TCGA-A2-A0YM-01	ER– HER2–
TCGA-A2-A25F-01	ER– HER2–
TCGA-A2-A3XS-01	ER– HER2–
TCGA-A2-A3XX-01	ER– HER2–
TCGA-A2-A3XY-01	ER– HER2–
TCGA-A7-A0DA-01	ER– HER2–
TCGA-A7-A13D-01	ER– HER2–
TCGA-A7-A13E-01	ER– HER2–
TCGA-A7-A26I-01	ER– HER2–
TCGA-A7-A4SD-01	ER– HER2–
TCGA-A7-A4SE-01	ER– HER2–
TCGA-A7-A6VV-01	ER– HER2–
TCGA-A7-A6VW-01	ER– HER2–
TCGA-A7-A6VY-01	ER– HER2–
TCGA-A8-A07O-01	ER– HER2–
TCGA-A8-A07R-01	ER– HER2–
TCGA-A8-A07U-01	ER– HER2–
TCGA-A8-A08R-01	ER– HER2–
TCGA-AC-A2BK-01	ER– HER2–
TCGA-AC-A5EH-01	ER– HER2–
TCGA-AC-A62X-01	ER– HER2–
TCGA-AC-A6IW-01	ER– HER2–
TCGA-AC-A8OQ-01	ER– HER2–
TCGA-AN-A04D-01	ER– HER2–
TCGA-AN-A0AL-01	ER– HER2–
TCGA-AN-A0AR-01	ER– HER2–
TCGA-AN-A0AT-01	ER– HER2–
TCGA-AN-A0FJ-01	ER– HER2–
TCGA-AN-A0FL-01	ER– HER2–
TCGA-AN-A0FX-01	ER– HER2–
TCGA-AO-A0J2-01	ER– HER2–
TCGA-AO-A0J4-01	ER– HER2–
TCGA-AO-A0J6-01	ER– HER2–
TCGA-AO-A128-01	ER– HER2–
TCGA-AO-A129-01	ER– HER2–
TCGA-AO-A12F-01	ER– HER2–
TCGA-AQ-A04J-01	ER– HER2–
TCGA-AR-A0TP-01	ER– HER2–
TCGA-AR-A0TS-01	ER– HER2–
TCGA-AR-A0U0-01	ER– HER2–
TCGA-AR-A0U4-01	ER– HER2–
TCGA-AR-A1AH-01	ER– HER2–
TCGA-AR-A1AJ-01	ER– HER2–

TCGA-AR-A1AO-01	ER− HER2−
TCGA-AR-A1AQ-01	ER− HER2−
TCGA-AR-A1AR-01	ER− HER2−
TCGA-AR-A1AY-01	ER− HER2−
TCGA-AR-A24Q-01	ER− HER2−
TCGA-AR-A251-01	ER− HER2−
TCGA-AR-A256-01	ER− HER2−
TCGA-B6-A0I1-01	ER− HER2−
TCGA-B6-A0IJ-01	ER− HER2−
TCGA-B6-A0IQ-01	ER− HER2−
TCGA-B6-A0RE-01	ER− HER2−
TCGA-B6-A0RS-01	ER− HER2−
TCGA-B6-A0RT-01	ER− HER2−
TCGA-B6-A400-01	ER− HER2−
TCGA-B6-A402-01	ER− HER2−
TCGA-B6-A409-01	ER− HER2−
TCGA-BH-A0AV-01	ER− HER2−
TCGA-BH-A0B3-01	ER− HER2−
TCGA-BH-A0B9-01	ER− HER2−
TCGA-BH-A0BG-01	ER− HER2−
TCGA-BH-A0BL-01	ER− HER2−
TCGA-BH-A0BW-01	ER− HER2−
TCGA-BH-A0E0-01	ER− HER2−
TCGA-BH-A0E6-01	ER− HER2−
TCGA-BH-A18G-01	ER− HER2−
TCGA-BH-A18T-01	ER− HER2−
TCGA-BH-A18V-01	ER− HER2−
TCGA-BH-A1F0-01	ER− HER2−
TCGA-BH-A1F6-01	ER− HER2−
TCGA-BH-A203-01	ER− HER2−
TCGA-BH-A208-01	ER− HER2−
TCGA-C8-A12V-01	ER− HER2−
TCGA-C8-A131-01	ER− HER2−
TCGA-C8-A134-01	ER− HER2−
TCGA-C8-A1HJ-01	ER− HER2−
TCGA-C8-A26X-01	ER− HER2−
TCGA-C8-A26Y-01	ER− HER2−
TCGA-C8-A27B-01	ER− HER2−
TCGA-D8-A13Z-01	ER− HER2−
TCGA-D8-A142-01	ER− HER2−
TCGA-D8-A143-01	ER− HER2−
TCGA-D8-A147-01	ER− HER2−
TCGA-D8-A1JF-01	ER− HER2−
TCGA-D8-A1JG-01	ER− HER2−
TCGA-D8-A1JK-01	ER− HER2−
TCGA-D8-A1JL-01	ER− HER2−

TCGA-D8-A1XK-01	ER– HER2–
TCGA-D8-A1XQ-01	ER– HER2–
TCGA-D8-A1XW-01	ER– HER2–
TCGA-D8-A27F-01	ER– HER2–
TCGA-D8-A27H-01	ER– HER2–
TCGA-D8-A27M-01	ER– HER2–
TCGA-E2-A14N-01	ER– HER2–
TCGA-E2-A14R-01	ER– HER2–
TCGA-E2-A14X-01	ER– HER2–
TCGA-E2-A150-01	ER– HER2–
TCGA-E2-A158-01	ER– HER2–
TCGA-E2-A159-01	ER– HER2–
TCGA-E2-A1AZ-01	ER– HER2–
TCGA-E2-A1B6-01	ER– HER2–
TCGA-E2-A1II-01	ER– HER2–
TCGA-E2-A1LH-01	ER– HER2–
TCGA-E2-A1LI-01	ER– HER2–
TCGA-E2-A1LK-01	ER– HER2–
TCGA-E2-A1LL-01	ER– HER2–
TCGA-E2-A573-01	ER– HER2–
TCGA-E9-A1N8-01	ER– HER2–
TCGA-E9-A1ND-01	ER– HER2–
TCGA-E9-A1RH-01	ER– HER2–
TCGA-E9-A22G-01	ER– HER2–
TCGA-E9-A243-01	ER– HER2–
TCGA-E9-A244-01	ER– HER2–
TCGA-E9-A248-01	ER– HER2–
TCGA-EW-A1OV-01	ER– HER2–
TCGA-EW-A1OW-01	ER– HER2–
TCGA-EW-A1P7-01	ER– HER2–
TCGA-EW-A1P8-01	ER– HER2–
TCGA-EW-A1PB-01	ER– HER2–
TCGA-EW-A1PH-01	ER– HER2–
TCGA-EW-A3U0-01	ER– HER2–
TCGA-GI-A2C9-01	ER– HER2–
TCGA-GM-A2DB-01	ER– HER2–
TCGA-GM-A2DD-01	ER– HER2–
TCGA-GM-A2DF-01	ER– HER2–
TCGA-GM-A3XL-01	ER– HER2–
TCGA-LL-A441-01	ER– HER2–
TCGA-LL-A5YO-01	ER– HER2–
TCGA-LL-A5YP-01	ER– HER2–
TCGA-LL-A73Y-01	ER– HER2–
TCGA-LL-A740-01	ER– HER2–
TCGA-LL-A8F5-01	ER– HER2–
TCGA-OL-A5D6-01	ER– HER2–

TCGA-OL-A5D7-01	ER− HER2−
TCGA-OL-A5RW-01	ER− HER2−
TCGA-OL-A5RZ-01	ER− HER2−
TCGA-OL-A5S0-01	ER− HER2−
TCGA-OL-A66I-01	ER− HER2−
TCGA-OL-A6VO-01	ER− HER2−
TCGA-PL-A8LZ-01	ER− HER2−
TCGA-S3-AA10-01	ER− HER2−
TCGA-S3-AA15-01	ER− HER2−
TCGA-3C-AALI-01	HER2+
TCGA-3C-AALK-01	HER2+
TCGA-A1-A0SM-01	HER2+
TCGA-A1-A0SN-01	HER2+
TCGA-A2-A04X-01	HER2+
TCGA-A2-A0CX-01	HER2+
TCGA-A2-A0D1-01	HER2+
TCGA-A2-A0EY-01	HER2+
TCGA-A2-A0T1-01	HER2+
TCGA-A2-A0YG-01	HER2+
TCGA-A2-A0YJ-01	HER2+
TCGA-A2-A1G1-01	HER2+
TCGA-A2-A3XZ-01	HER2+
TCGA-A7-A2KD-01	HER2+
TCGA-A7-A4SF-01	HER2+
TCGA-A8-A076-01	HER2+
TCGA-A8-A07B-01	HER2+
TCGA-A8-A07I-01	HER2+
TCGA-A8-A08B-01	HER2+
TCGA-A8-A08J-01	HER2+
TCGA-A8-A08S-01	HER2+
TCGA-A8-A08X-01	HER2+
TCGA-A8-A09G-01	HER2+
TCGA-A8-A09I-01	HER2+
TCGA-AC-A23C-01	HER2+
TCGA-AC-A23H-01	HER2+
TCGA-AN-A04C-01	HER2+
TCGA-AN-A0FT-01	HER2+
TCGA-AN-A0FV-01	HER2+
TCGA-AO-A0JE-01	HER2+
TCGA-AO-A0JL-01	HER2+
TCGA-AO-A0JM-01	HER2+
TCGA-AO-A12D-01	HER2+
TCGA-AQ-A04H-01	HER2+
TCGA-AQ-A0Y5-01	HER2+
TCGA-AR-A0TQ-01	HER2+
TCGA-AR-A0TX-01	HER2+

TCGA-AR-A24U-01	HER2+
TCGA-AR-A250-01	HER2+
TCGA-AR-A254-01	HER2+
TCGA-B6-A0I9-01	HER2+
TCGA-B6-A0IG-01	HER2+
TCGA-B6-A0RH-01	HER2+
TCGA-B6-A1KF-01	HER2+
TCGA-B6-A1KN-01	HER2+
TCGA-BH-A0AW-01	HER2+
TCGA-BH-A0B7-01	HER2+
TCGA-BH-A0DD-01	HER2+
TCGA-BH-A0DZ-01	HER2+
TCGA-BH-A0EE-01	HER2+
TCGA-BH-A0HY-01	HER2+
TCGA-BH-A18R-01	HER2+
TCGA-BH-A18U-01	HER2+
TCGA-BH-A1EN-01	HER2+
TCGA-BH-A1EV-01	HER2+
TCGA-BH-A1F2-01	HER2+
TCGA-BH-A1FU-01	HER2+
TCGA-BH-A202-01	HER2+
TCGA-C8-A12L-01	HER2+
TCGA-C8-A12P-01	HER2+
TCGA-C8-A12Q-01	HER2+
TCGA-C8-A12T-01	HER2+
TCGA-C8-A12Z-01	HER2+
TCGA-C8-A132-01	HER2+
TCGA-C8-A135-01	HER2+
TCGA-C8-A137-01	HER2+
TCGA-C8-A1HF-01	HER2+
TCGA-C8-A1HK-01	HER2+
TCGA-C8-A26W-01	HER2+
TCGA-C8-A275-01	HER2+
TCGA-C8-A278-01	HER2+
TCGA-D8-A1J9-01	HER2+
TCGA-D8-A1JA-01	HER2+
TCGA-D8-A1X5-01	HER2+
TCGA-D8-A1XT-01	HER2+
TCGA-D8-A27N-01	HER2+
TCGA-E2-A14P-01	HER2+
TCGA-E2-A14V-01	HER2+
TCGA-E2-A14Y-01	HER2+
TCGA-E2-A152-01	HER2+
TCGA-E2-A15H-01	HER2+
TCGA-E2-A1B0-01	HER2+
TCGA-E2-A1LB-01	HER2+

TCGA-E2-A1LE-01	HER2+
TCGA-E9-A1N6-01	HER2+
TCGA-E9-A228-01	HER2+
TCGA-E9-A22D-01	HER2+
TCGA-EW-A1OZ-01	HER2+
TCGA-EW-A2FR-01	HER2+
TCGA-EW-A6S9-01	HER2+
TCGA-EW-A6SD-01	HER2+
TCGA-GM-A2DA-01	HER2+
TCGA-LD-A9QF-01	HER2+
TCGA-OL-A5RY-01	HER2+
TCGA-S3-AA14-01	HER2+
TCGA-UL-AAZ6-01	HER2+
TCGA-UU-A93S-01	HER2+
TCGA-A7-A0CE-11	Normal
TCGA-A7-A0CH-11	Normal
TCGA-A7-A0D9-11	Normal
TCGA-A7-A0DB-11	Normal
TCGA-A7-A13E-11	Normal
TCGA-A7-A13F-11	Normal
TCGA-A7-A13G-11	Normal
TCGA-AC-A23H-11	Normal
TCGA-AC-A2FB-11	Normal
TCGA-AC-A2FF-11	Normal
TCGA-AC-A2FM-11	Normal
TCGA-BH-A0AU-11	Normal
TCGA-BH-A0AY-11	Normal
TCGA-BH-A0AZ-11	Normal
TCGA-BH-A0B3-11	Normal
TCGA-BH-A0B5-11	Normal
TCGA-BH-A0B7-11	Normal
TCGA-BH-A0B8-11	Normal
TCGA-BH-A0BA-11	Normal
TCGA-BH-A0BC-11	Normal
TCGA-BH-A0BJ-11	Normal
TCGA-BH-A0BM-11	Normal
TCGA-BH-A0BQ-11	Normal
TCGA-BH-A0BS-11	Normal
TCGA-BH-A0BT-11	Normal
TCGA-BH-A0BV-11	Normal
TCGA-BH-A0BW-11	Normal
TCGA-BH-A0BZ-11	Normal
TCGA-BH-A0C0-11	Normal
TCGA-BH-A0C3-11	Normal
TCGA-BH-A0DD-11	Normal
TCGA-BH-A0DG-11	Normal

TCGA-BH-A0DH-11	Normal
TCGA-BH-A0DK-11	Normal
TCGA-BH-A0DL-11	Normal
TCGA-BH-A0DO-11	Normal
TCGA-BH-A0DP-11	Normal
TCGA-BH-A0DQ-11	Normal
TCGA-BH-A0DT-11	Normal
TCGA-BH-A0DV-11	Normal
TCGA-BH-A0DZ-11	Normal
TCGA-BH-A0E0-11	Normal
TCGA-BH-A0E1-11	Normal
TCGA-BH-A0H5-11	Normal
TCGA-BH-A0H7-11	Normal
TCGA-BH-A0H9-11	Normal
TCGA-BH-A0HA-11	Normal
TCGA-BH-A0HK-11	Normal
TCGA-BH-A18J-11	Normal
TCGA-BH-A18K-11	Normal
TCGA-BH-A18L-11	Normal
TCGA-BH-A18M-11	Normal
TCGA-BH-A18N-11	Normal
TCGA-BH-A18P-11	Normal
TCGA-BH-A18Q-11	Normal
TCGA-BH-A18R-11	Normal
TCGA-BH-A18S-11	Normal
TCGA-BH-A18U-11	Normal
TCGA-BH-A18V-11	Normal
TCGA-BH-A1EN-11	Normal
TCGA-BH-A1EO-11	Normal
TCGA-BH-A1ET-11	Normal
TCGA-BH-A1EU-11	Normal
TCGA-BH-A1EV-11	Normal
TCGA-BH-A1EW-11	Normal
TCGA-BH-A1F0-11	Normal
TCGA-BH-A1F2-11	Normal
TCGA-BH-A1F6-11	Normal
TCGA-BH-A1F8-11	Normal
TCGA-BH-A1FB-11	Normal
TCGA-BH-A1FC-11	Normal
TCGA-BH-A1FD-11	Normal
TCGA-BH-A1FE-11	Normal
TCGA-BH-A1FG-11	Normal
TCGA-BH-A1FH-11	Normal
TCGA-BH-A1FJ-11	Normal
TCGA-BH-A1FM-11	Normal
TCGA-BH-A1FN-11	Normal

TCGA-BH-A1FR-11	Normal
TCGA-BH-A1FU-11	Normal
TCGA-BH-A203-11	Normal
TCGA-BH-A204-11	Normal
TCGA-BH-A208-11	Normal
TCGA-BH-A209-11	Normal
TCGA-E2-A153-11	Normal
TCGA-E2-A158-11	Normal
TCGA-E2-A15I-11	Normal
TCGA-E2-A15K-11	Normal
TCGA-E2-A15M-11	Normal
TCGA-E2-A1BC-11	Normal
TCGA-E2-A1IG-11	Normal
TCGA-E2-A1L7-11	Normal
TCGA-E2-A1LB-11	Normal
TCGA-E2-A1LH-11	Normal
TCGA-E2-A1LS-11	Normal
TCGA-E9-A1N4-11	Normal
TCGA-E9-A1N5-11	Normal
TCGA-E9-A1N6-11	Normal
TCGA-E9-A1N9-11	Normal
TCGA-E9-A1NA-11	Normal
TCGA-E9-A1ND-11	Normal
TCGA-E9-A1NF-11	Normal
TCGA-E9-A1NG-11	Normal
TCGA-E9-A1R7-11	Normal
TCGA-E9-A1RB-11	Normal
TCGA-E9-A1RC-11	Normal
TCGA-E9-A1RD-11	Normal
TCGA-E9-A1RF-11	Normal
TCGA-E9-A1RH-11	Normal
TCGA-E9-A1RI-11	Normal
TCGA-GI-A2C8-11	Normal
TCGA-GI-A2C9-11	Normal

Supplementary Figure S4

Exon expression and median adjustment

A S2 : Expression of each Exon

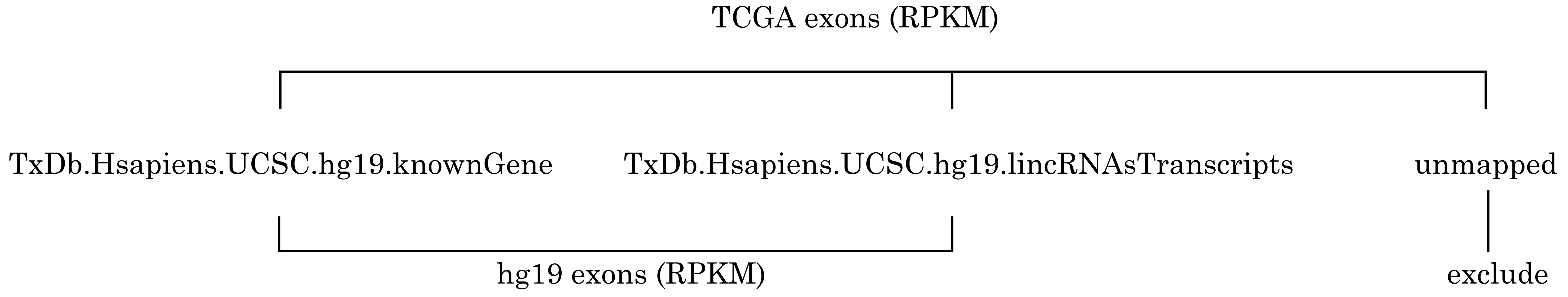

B in each sample, set median of all exons to 1 ➟ expression = $\log_2(1 + 1023 \cdot \text{median-adjusted-RPKM})$

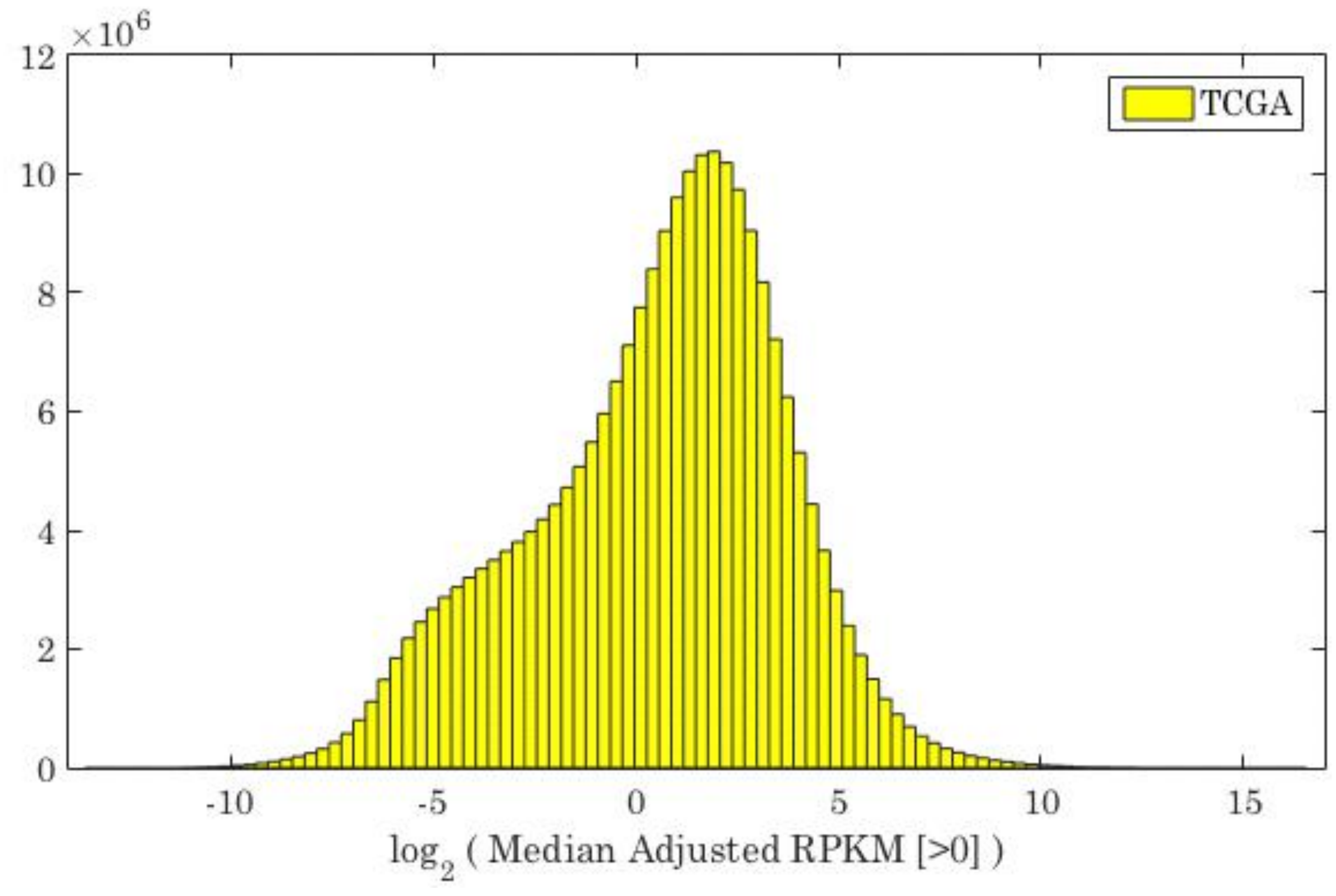

Supplementary Figure S4. *Determining exon expression and median adjustment.* RNAseqV2 exon expression data (RPKM) were obtained from Broad GDAC. The coordinates of the exons were mapped to exons of known genes and lincRNAs, as defined in TxDb.Hsapiens.UCSC.hg19.knownGene, and TxDb.Hsapiens.UCSC.hg19.lincRNAsTranscripts, unmapped exons, and exons with 0 RPKM in all samples were excluded from the analysis (A). The exon expression levels were defined as $\log_2(1 + a * \text{median-adjusted RPKM})$, where 1/a is added to avoid log(0). $\log_2$ RPKM values for all exons in all samples were plotted (B), and as most expressed exons showed $\log_2$ RPKM > -10, a was set to 2^10 -1 = 1023 in TCGA. Thereby exons that are not at all expressed have expression level 0, and within each sample the median expression level of all exons is 10.

Supplementary Figure S5
Determining mode exon expression within each biological class.

S3 : Mode of each Exon — function : bkde — R package : KernSmooth

A for each exon, in each class ➟ distribution of expression

B number of modes: 1 | >1

C ratio of height : highest peak / 2nd-highest peak: < 2 | ≥ 2

< 2 → exclude

≥ 2 → consider only the highest peak

Supplementary Figure S5. *Determining mode expression of exons in each biological class.* The distribution of the expression of each exon was plotted for each biological class (60% of the samples) (A), the number of modes were determined using the bkde function in KernSmooth R package, and the mode was calculated (B). If the distribution of expression values in the class showed > 1 mode the mode was defined as the highest peak only if the ratio of height highest peak / 2nd-highest peak was ≥ 2. Exons with no clear highest peak (ratio <2) were excluded from the analysis (C).

Supplementary Figure S6
Determining log_2FC between two classes

S4 : log_2FC between classes — function : bkde — R package : KernSmooth

A

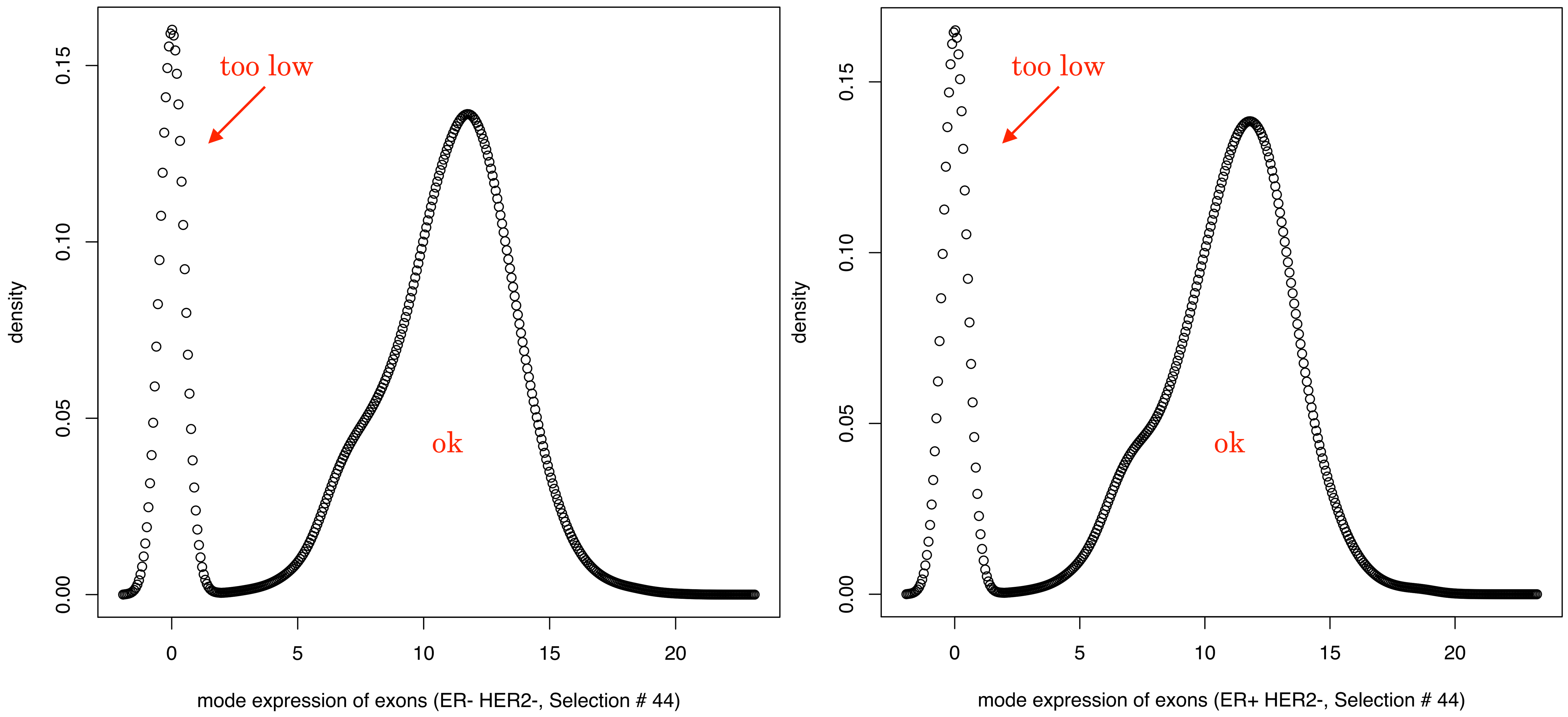

B

ER– HER2–	ER+ HER2–	log_2FC
too low	too low	exclude exon
too low	ok	mode(ER– HER2–) – mode(ER+ HER2–)
ok	too low	mode(ER– HER2–) – mode(ER+ HER2–)
ok	ok	mode(ER– HER2–) – mode(ER+ HER2–)

Supplementary Figure S6. *Determining log_2FC between two classes.* The distribution of mode expression of all exons were plotted for each biological class (random 60% of the samples)(A). Log_2FC was calculated between two classes for exons that showed expression in at least one class (B), The mode of 60% selection of samples was plotted 100 times for each biological class, resulting in 10.000 log_2FC estimates per class comparison.

Supplementary Figure S7

Calling of outlier exons

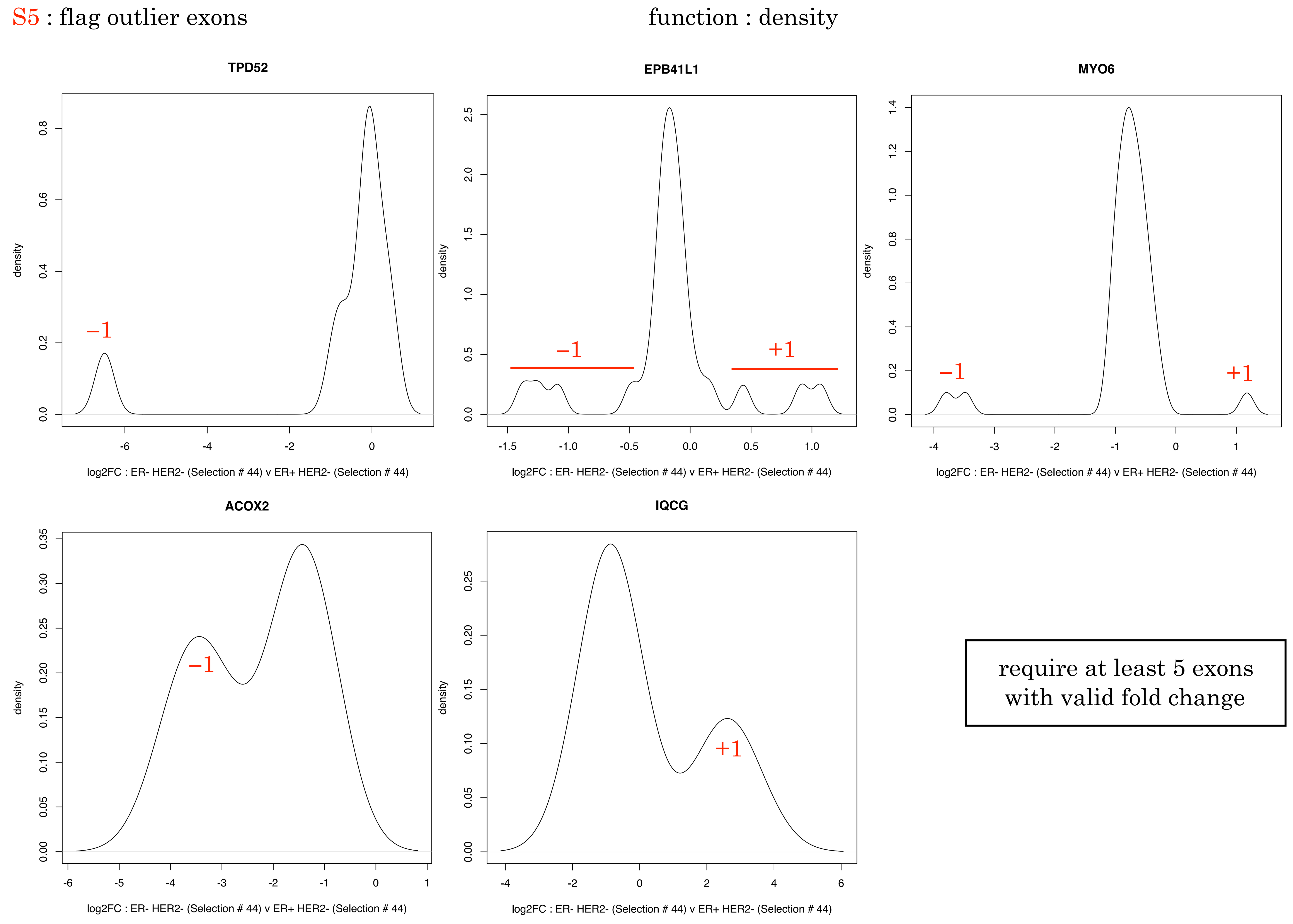

Supplementary Figure S7. *Calling of outlier exons* . The distribution (density plot) of $\log_2$ fold change for all exons in each gene was computed. The density plots shown compares samples in the ER-/HER2- to ER+/HER2- samples (one selection of 60% of all samples in each group). The highest peak represents an overall scaling effect, affecting a majority of exons. Exons in peaks with smaller amplitude were identified as exons that are differentially spliced or transcribed. Peaks to the right indicate exons with greater Log_2 fold change compared to the exons in the central peak. Peaks to the left of the central peak are exons with smaller Log_2 fold change than the exons in the central peak. The density plots were created using the “density” function in R. Only genes with valid fold change in 5 exons were considered in the downstream analysis.

Supplementary Figure S8
Determining differential exon usage among two biological classes

A

S6 : say for an exon, $n(+1) = a$, $n(-1) = b$, $n(0) = c$, $n(NA) = 10^4 - (a + b + c)$ ➟ $\text{score} = a - b$

example : ER– HER2– vs ER+ HER2–

B

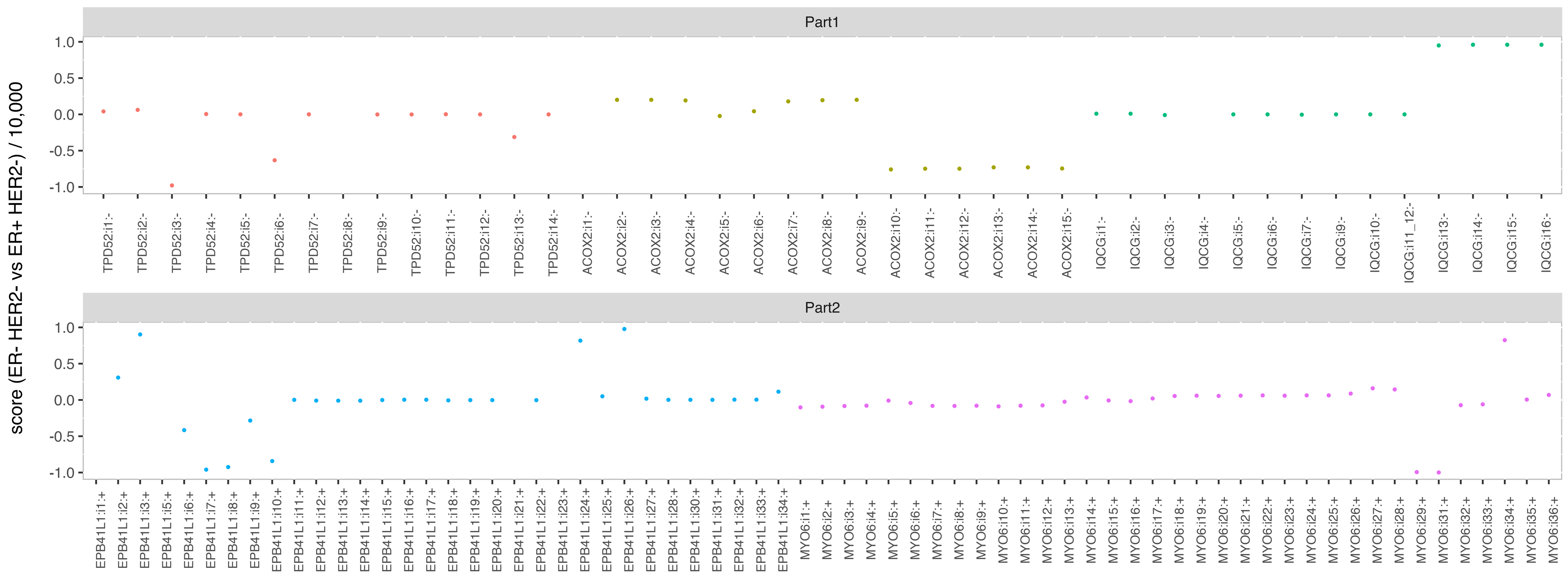

C

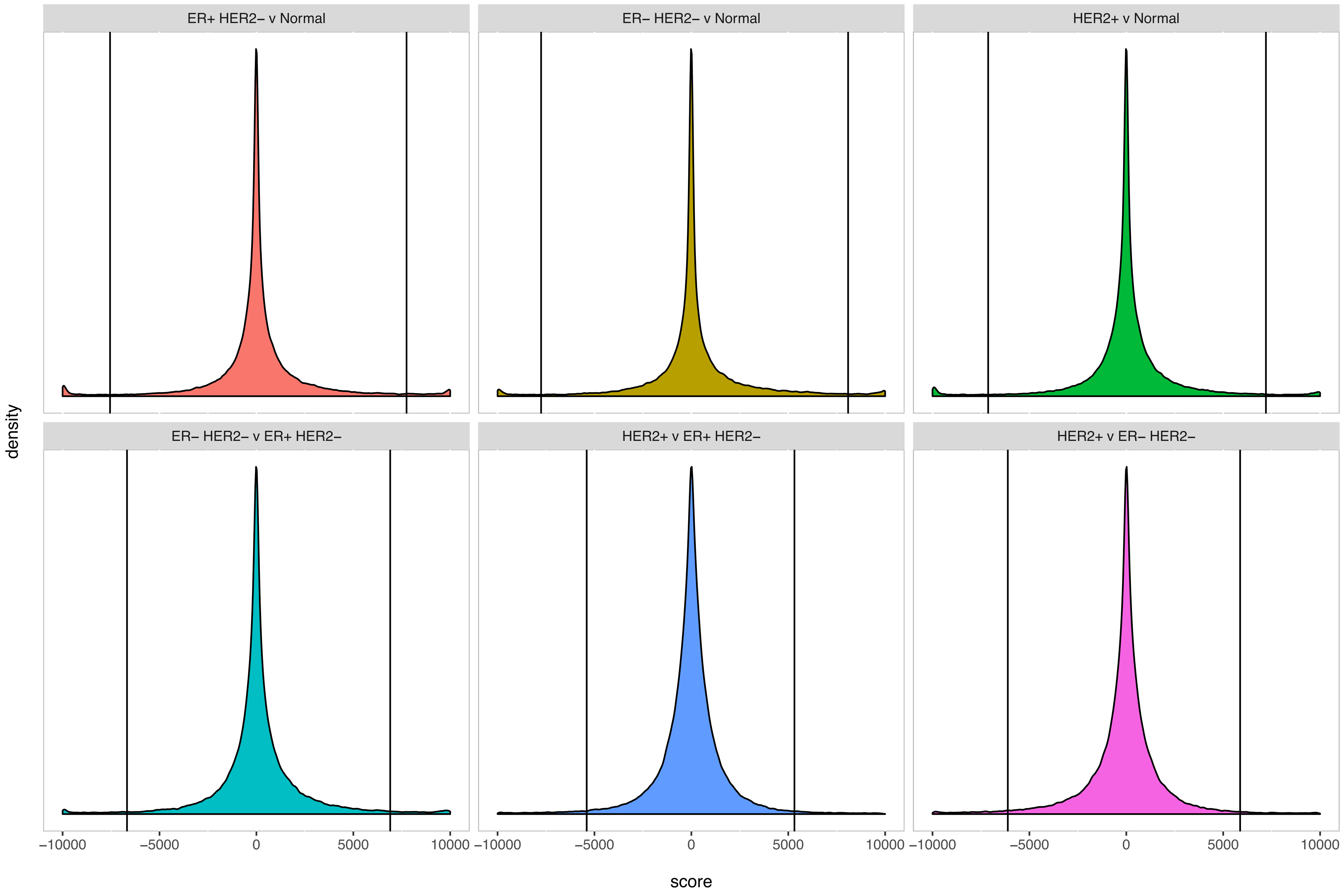

Supplementary Figure S8. *Determining differential exon usage among two biological classes.* S2-S3 were repeated for random selections of 60% of all samples within a biological class 100 times. For each test (S4 and S5) an exon was called either +1 (a), -1 (b), or 0 (c), so that each exon is tested 10.000 times, and the total score for each exon was determined as a-b (A). The total scores for each exon in 5 genes, TPD52, ACOX2, IQCG, EPB41L1, and MYO6 when comparing ER- Her2- and ER+ HER2- samples are shown in B. (C) The distribution of the sum of the 10,000 numbers was plotted for each pairwise comparisons and only exons with scores exceeding a conservative threshold of 3 standard deviations were called as differentially spliced/transcribed.

Supplementary Figure S9

Identification of exon with higher (+1) and lower (-1) FC relative to the overall scaling of gene expression, that is, FC of most exons

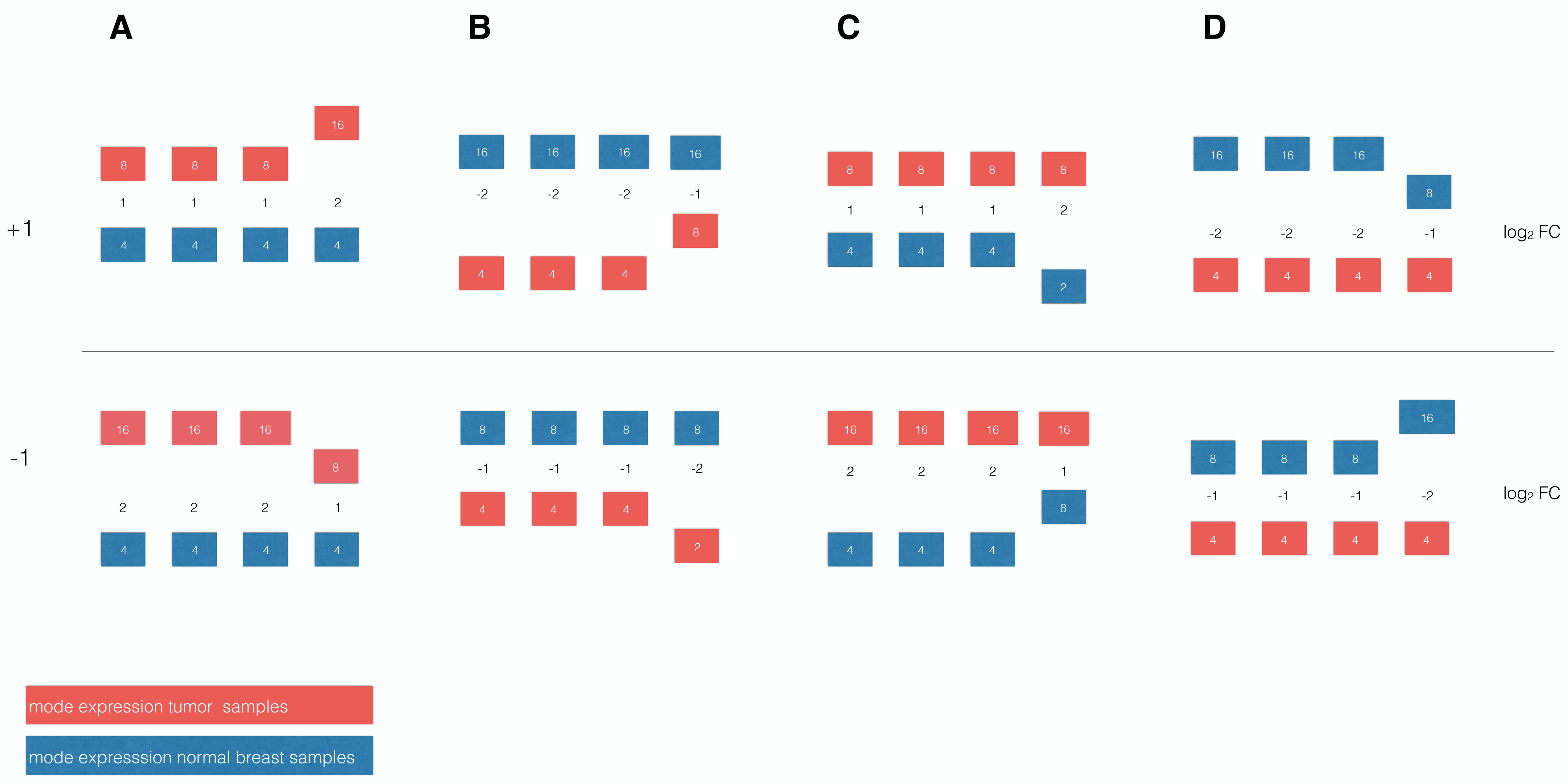

Supplementary Figure S9. *Illustration of exons with differential usage among two conditions.* The mode expression of 4 exons in two conditions, tumor and normal breast, are shown to illustrate different scenarios where an exon is called with higher Fold Change relative to the majority of the exons in the gene (upper panel, +1) in the tumor versus normal comparison. This includes exons with higher expression/inclusion in the tumor samples, irrespective of whether the overall expression of the gene is higher (A) or lower (B) in the tumor samples. The +1 exons also include exons with lower expression/inclusion in the normal breast samples (C and D). The lower panel shows different scenarios where an exon is called with lower Fold Change relative to the majority of the exons in the gene (-1) in the tumor versus normal comparison. These include exons with lower expression/inclusion in the tumor compared to normal samples (A and B), and exons with higher expression/inclusion in the normal samples (C and D).

Supplementary Table S11. *Exons flagged in TCGA and Radium/Rutgers dataset*

Exon	Entrez	Symbol	Coordinate	sign_BL
22848:i1:-	22848	AAK1	chr2:69870707-69870977:-	1
10152:i18:+	10152	ABI2	chr2:204291900-204296892:+	-1
79777:i2:+	79777	ACBD4	chr17:43212708-43212963:+	-1
93:i12:+	93	ACVR2B	chr3:38524629-38534633:+	-1
11033:i1:-	11033	ADAP1	chr7:1015064-1015235:-	1
118:i24:+	118	ADD1	chr4:2928369-2928402:+	1
9289:i1:+	9289	ADGRG1	chr16:57653650-57653793:+	1
84059:i96:+	84059	ADGRV1	chr5:90459599-90460033:+	1
27125:i1:-	27125	AFF4	chr5:132298951-132299354:-	1
729092:i20:-	729092	AGAP5	chr10:75434033-75435832:-	1
10598:i9:+	10598	AHSA1	chr14:77934933-77935069:+	-1
55057:i2:-	55057	AIM1L	chr1:26670236-26673203:-	-1
211:i2:+	211	ALAS1	chr3:52232688-52232864:+	1
226:i11:+	226	ALDOA	chr16:30078206-30078359:+	-1
226:i4:+	226	ALDOA	chr16:30066105-30066248:+	-1
51281:i17:-	51281	ANKMY1	chr2:241448765-241448872:-	-1
81573:i1:-	81573	ANKRD13C	chr1:70819662-70820417:-	1
338692:i2:+	338692	ANKRD13D	chr11:67057300-67057436:+	1
54522:i1:-	54522	ANKRD16	chr10:5931004-5931860:-	1
378708:i1:+	378708	APITD1	chr1:10490159-10490625:+	1
100526739:i1:+	100526739	APITD1-CORT	chr1:10490159-10490625:+	1
139322:i9:+	139322	APOOL	chrX:84342596-84348323:+	-1
116985:i1:-	116985	ARAP1	chr11:72503920-72504750:-	-1
58504:i5:-	58504	ARHGAP22	chr10:49790998-49791197:-	-1
57514:i12:+	57514	ARHGAP31	chr3:119132703-119138323:+	1
51742:i30:-	51742	ARID4B	chr1:235324489-235324772:-	1
51742:i31:-	51742	ARID4B	chr1:235324208-235324382:-	1
51742:i36:-	51742	ARID4B	chr1:235301406-235301489:-	1
51742:i37:-	51742	ARID4B	chr1:235301192-235301255:-	1
51742:i38:-	51742	ARID4B	chr1:235299349-235299388:-	1
408:i13:-	408	ARRB1	chr11:74982745-74982768:-	1
421:i20:-	421	ARVCF	chr22:19958739-19958858:-	-1
51665:i1:+	51665	ASB1	chr2:239335626-239335760:+	1
55870:i4:-	55870	ASH1L	chr1:155490891-155491409:-	-1
55054:i10:+	55054	ATG16L1	chr2:234182367-234182423:+	1
10533:i19:+	10533	ATG7	chr3:11596285-11599139:+	1
472:i1:+	472	ATM	chr11:108093559-108093913:+	1
100526740:i1:-	100526740	ATP5J2-PTCD1	chr7:99063752-99063824:-	1
525:i1:+	525	ATP6V1B1	chr2:71162998-71163202:+	-1
26053:i1:+	26053	AUTS2	chr7:69063905-69064948:+	1
26053:i2:+	26053	AUTS2	chr7:69364272-69364484:+	1
26053:i3:+	26053	AUTS2	chr7:69583118-69583219:+	1
8938:i40:+	8938	BAIAP3	chr16:1398380-1399442:+	1
582:i1:+	582	BBS1	chr11:66276550-66276785:+	1
4059:i1:+	4059	BCAM	chr19:45312316-45312463:+	1
9274:i7:-	9274	BCL7C	chr16:30846270-30846469:-	-1
654:i1:+	654	BMP6	chr6:7727011-7727852:+	1
670:i2:+	670	BPHL	chr6:3119519-3119746:+	1
148362:i3:+	148362	BROX	chr1:222886930-222887245:+	1
682:i1:+	682	BSG	chr19:571277-571579:+	-1
22903:i1:+	22903	BTBD3	chr20:11871477-11871602:+	1
100506581:i7:-	100506581	C16orf95	chr16:87339325-87339511:-	1
100506581:i8:-	100506581	C16orf95	chr16:87336404-87336681:-	1
54991:i6:-	54991	C1orf159	chr1:1026658-1026754:-	-1
54991:i7:-	54991	C1orf159	chr1:1026256-1026363:-	-1
221477:i5:+	221477	C6orf89	chr6:36862280-36862379:+	1
100996928:i1:+	100996928	C7orf55-LUC7L2	chr7:139025878-139026268:+	1

100996928:i2:+	100996928	C7orf55-LUC7L2	chr7:139030247-139030367:+	1
377677:i1:+	377677	CA13	chr8:86132845-86133354:+	-1
11238:i3:+	11238	CA5B	chrX:15782701-15782898:+	1
767:i11:-	767	CA8	chr8:61101423-61102544:-	-1
10203:i3:-	10203	CALCRL	chr2:188293064-188293616:-	1
800:i4:+	800	CALD1	chr7:134576151-134576424:+	1
57662:i1:+	57662	CAMSAP3	chr19:7660788-7661036:+	-1
23261:i1:+	23261	CAMTA1	chr1:6845384-6845635:+	1
54897:i2:-	54897	CASZ1	chr1:10820758-10820914:-	-1
54897:i4:-	54897	CASZ1	chr1:10754354-10754507:-	1
875:i7:-	875	CBS	chr21:44487888-44488514:-	-1
57545:i4:+	57545	CC2D2A	chr4:15480347-15480430:+	-1
90693:i4:+	90693	CCDC126	chr7:23643761-23643898:+	-1
84865:i14:-	84865	CCDC142	chr2:74699085-74699379:-	-1
283152:i1:-	283152	CCDC153	chr11:119066509-119066710:-	1
901:i1:+	901	CCNG2	chr4:78078357-78078659:+	1
54619:i2:+	54619	CCNJ	chr10:97804110-97804219:+	1
961:i10:-	961	CD47	chr3:107768466-107768498:-	-1
961:i9:-	961	CD47	chr3:107769425-107769449:-	-1
56882:i2:-	56882	CDC42SE1	chr1:151029127-151029262:-	-1
79577:i17:+	79577	CDC73	chr1:193219806-193223942:+	-1
10658:i3:-	10658	CELF1	chr11:47522413-47522484:-	1
8837:i2:+	8837	CFLAR	chr2:201983269-201983624:+	1
1111:i1:+	1111	CHEK1	chr11:125495031-125495907:+	-1
1195:i2:-	1195	CLK1	chr2:201728824-201729284:-	1
1211:i1:+	1211	CLTA	chr9:36190853-36191270:+	1
1212:i3:-	1212	CLTB	chr5:175833158-175833709:-	-1
134147:i1:-	134147	CMBL	chr5:10307737-10308168:-	1
51727:i1:+	51727	CMPK1	chr1:47799469-47799788:+	1
1303:i58:-	1303	COL12A1	chr6:75813473-75813526:-	1
1303:i59:-	1303	COL12A1	chr6:75812313-75812408:-	1
1303:i60:-	1303	COL12A1	chr6:75811715-75811768:-	1
1303:i61:-	1303	COL12A1	chr6:75811316-75811369:-	1
1303:i62:-	1303	COL12A1	chr6:75806969-75807022:-	1
1303:i63:-	1303	COL12A1	chr6:75804824-75804895:-	1
1303:i64:-	1303	COL12A1	chr6:75801206-75801241:-	1
80781:i3:+	80781	COL18A1	chr21:46875424-46876090:+	1
1293:i3:-	1293	COL6A3	chr2:238303230-238303847:-	-1
80315:i4:+	80315	CPEB4	chr5:173359453-173359503:+	-1
9244:i8:-	9244	CRLF1	chr19:18704875-18704917:-	1
9946:i8:-	9946	CRYZL1	chr21:34988594-34988909:-	1
53944:i2:-	53944	CSNK1G1	chr15:64665694-64665968:-	1
1456:i12:+	1456	CSNK1G3	chr5:122930733-122930828:+	1
79848:i7:+	79848	CSPP1	chr8:68004038-68004118:+	-1
1479:i1:-	1479	CSTF3	chr11:33182845-33183037:-	1
9150:i1:+	9150	CTDP1	chr18:77439801-77440261:+	1
1500:i24:+	1500	CTNND1	chr11:57583387-57583473:+	-1
1515:i3:-	1515	CTSV	chr9:99801353-99801592:-	1
285440:i1:+	285440	CYP4V2	chr4:187112674-187113191:+	1
747:i20:+	747	DAGLA	chr11:61511004-61514474:+	1
26528:i13:+	26528	DAZAP1	chr19:1433738-1433826:+	-1
1634:i1:-	1634	DCN	chr12:91576432-91576806:-	-1
51202:i1:+	51202	DDX47	chr12:12878851-12879030:+	1
54849:i6:+	54849	DEF8	chr16:90020651-90020784:+	1
8525:i2:+	8525	DGKZ	chr11:46366817-46367135:+	-1
8525:i4:+	8525	DGKZ	chr11:46383145-46383256:+	-1
8525:i5:+	8525	DGKZ	chr11:46387794-46388534:+	-1
79758:i9:-	79758	DHRS12	chr13:52344913-52345631:-	1
10395:i2:-	10395	DLC1	chr8:13356558-13357705:-	-1

1741:i9:+	1741	DLG3	chrX:69673487-69673643:+	1
127602:i1:+	127602	DNAH14	chr1:225117356-225117537:+	1
10049:i12:+	10049	DNAJB6	chr7:157202489-157202695:+	1
131118:i8:-	131118	DNAJC19	chr3:180701498-180702498:-	-1
134218:i15:+	134218	DNAJC21	chr5:34954658-34959069:+	-1
548645:i2:+	548645	DNAJC25	chr9:114405137-114405374:+	1
1786:i36:-	1786	DNMT1	chr19:10251457-10251585:-	1
1786:i37:-	1786	DNMT1	chr19:10250722-10251004:-	1
1786:i38:-	1786	DNMT1	chr19:10250352-10250493:-	1
1786:i39:-	1786	DNMT1	chr19:10249115-10249281:-	1
1786:i40:-	1786	DNMT1	chr19:10248508-10248685:-	1
1786:i42:-	1786	DNMT1	chr19:10247752-10247956:-	1
1786:i43:-	1786	DNMT1	chr19:10246797-10246963:-	1
1786:i44:-	1786	DNMT1	chr19:10246412-10246528:-	1
1786:i45:-	1786	DNMT1	chr19:10244893-10244983:-	1
29980:i1:-	29980	DONSON	chr21:35284593-35284703:-	-1
29980:i2:-	29980	DONSON	chr21:35281386-35281515:-	-1
29980:i3:-	29980	DONSON	chr21:35279645-35279757:-	-1
29980:i4:-	29980	DONSON	chr21:34967662-34967920:-	-1
29980:i5:-	29980	DONSON	chr21:34963468-34963677:-	-1
29980:i6:-	29980	DONSON	chr21:34962147-34962227:-	-1
89978:i4:-	89978	DPH6	chr15:35812474-35814479:-	-1
92715:i4:-	92715	DPH7	chr9:140470761-140470854:-	-1
92715:i6:-	92715	DPH7	chr9:140470161-140470619:-	-1
667:i19:-	667	DST	chr6:56507420-56507694:-	-1
1778:i78:+	1778	DYNC1H1	chr14:102516051-102516219:+	1
1859:i1:+	1859	DYRK1A	chr21:38739859-38739930:+	1
1871:i1:+	1871	E2F3	chr6:20402137-20402856:+	1
1891:i1:-	1891	ECH1	chr19:39322361-39322497:-	1
55268:i5:-	55268	ECHDC2	chr1:53380803-53380964:-	-1
79746:i1:+	79746	ECHDC3	chr10:11784356-11784745:+	1
55741:i2:-	55741	EDEM2	chr20:33859808-33859948:-	-1
100526837:i1:-	100526837	EEF1E1-BLOC1S5	chr6:8102668-8102828:-	1
90141:i8:-	90141	EFCAB11	chr14:90389670-90391021:-	1
8893:i1:+	8893	EIF2B5	chr3:183852810-183853368:+	1
9470:i9:+	9470	EIF4E2	chr2:233445614-233448349:+	-1
60481:i5:-	60481	ELOVL5	chr6:53158888-53159289:-	1
284131:i6:+	284131	ENDOV	chr17:78393850-78394333:+	-1
2035:i17:+	2035	EPB41	chr1:29385101-29385157:+	-1
2036:i24:+	2036	EPB41L1	chr20:34793771-34795702:+	1
2036:i26:+	2036	EPB41L1	chr20:34797410-34797820:+	1
54566:i27:-	54566	EPB41L4B	chr9:111934254-111936903:-	-1
1161:i3:-	1161	ERCC8	chr5:60220118-60220335:-	-1
79956:i1:-	79956	ERMP1	chr9:5832690-5833081:-	1
54845:i15:+	54845	ESRP1	chr8:95704905-95705055:+	1
2110:i2:+	2110	ETFDH	chr4:159600442-159601238:+	1
55224:i1:-	55224	ETNK2	chr1:204120723-204121307:-	1
2113:i3:-	2113	ETS1	chr11:128426186-128426330:-	-1
23016:i12:+	23016	EXOSC7	chr3:45053675-45054158:+	-1
11340:i1:+	11340	EXOSC8	chr13:37574678-37574959:+	1
2166:i1:+	2166	FAAH	chr1:46859939-46860215:+	-1
54491:i1:+	54491	FAM105A	chr5:14581891-14582067:+	1
10144:i1:-	10144	FAM13A	chr4:90032193-90032549:-	1
729085:i1:+	729085	FAM198A	chr3:43020759-43021137:+	1
100631383:i8:+	100631383	FAM47E-STBD1	chr4:77230297-77232283:+	1
2195:i29:-	2195	FAT1	chr4:187511522-187511557:-	-1
2192:i18:+	2192	FBLN1	chr22:45960764-45961581:+	1
2200:i64:-	2200	FBN1	chr15:48712884-48713003:-	1
64839:i1:-	64839	FBXL17	chr5:107716400-107717799:-	1

89846:i1:+	89846	FGD3	chr9:95709601-95710011:+	-1
81608:i1:+	81608	FIP1L1	chr4:54243820-54244090:+	1
2321:i19:-	2321	FLT1	chr13:28942234-28942800:-	1
64838:i1:-	64838	FNDC4	chr2:27717745-27718126:-	1
3607:i9:+	3607	FOXK2	chr17:80551298-80551830:+	-1
1112:i3:-	1112	FOXN3	chr14:89883215-89883454:-	1
27086:i7:-	27086	FOXP1	chr3:71381345-71384432:-	1
23150:i1:-	23150	FRMD4B	chr3:69591357-69591733:-	1
285527:i5:-	285527	FRYL	chr4:48638867-48640580:-	1
26301:i1:-	26301	GBGT1	chr9:136039141-136039301:-	1
2643:i1:-	2643	GCH1	chr14:55369039-55369542:-	1
85476:i6:+	85476	GFM1	chr3:158367794-158367850:+	1
2677:i1:-	2677	GGCX	chr2:85788509-85788657:-	1
26058:i2:+	26058	GIGYF2	chr2:233565295-233565364:+	1
2710:i22:+	2710	GK	chrX:30745583-30745669:+	1
51292:i9:+	51292	GMPR2	chr14:24703313-24703447:+	1
2803:i20:+	2803	GOLGA4	chr3:37378634-37378654:+	1
51280:i1:-	51280	GOLM1	chr9:88897293-88897490:-	1
2813:i3:-	2813	GP2	chr16:20335138-20335578:-	1
56261:i1:-	56261	GPCPD1	chr20:5591488-5591672:-	1
29841:i1:+	29841	GRHL1	chr2:10091792-10091982:+	1
2932:i12:-	2932	GSK3B	chr3:119540802-119545702:-	-1
112495:i1:+	112495	GTF3C6	chr6:111279763-111280029:+	1
51454:i1:+	51454	GULP1	chr2:189156396-189156893:+	1
120071:i4:+	120071	GYLTL1B	chr11:45944372-45944718:+	1
3052:i9:+	3052	HCCS	chrX:11139732-11141204:+	-1
9734:i42:+	9734	HDAC9	chr7:19035646-19036992:+	-1
113802:i1:-	113802	HENMT1	chr1:109203806-109204148:-	1
3077:i5:+	3077	HFE	chr6:26092837-26092869:+	1
9146:i20:+	9146	HGS	chr17:79667497-79667630:+	1
59269:i2:-	59269	HIVEP3	chr1:42384084-42384496:-	-1
3107:i4:-	3107	HLA-C	chr6:31238850-31239125:-	1
3150:i5:-	3150	HMGN1	chr21:40717756-40718454:-	-1
23526:i28:+	23526	HMHA1	chr19:1085659-1086627:+	-1
3176:i2:+	3176	HNMT	chr2:138724667-138725014:+	1
3185:i3:-	3185	HNRNPF	chr10:43904175-43904332:-	-1
3241:i1:+	3241	HPCAL1	chr2:10443030-10443303:+	1
51171:i1:-	51171	HSD17B14	chr19:49339581-49339934:-	1
3320:i1:-	3320	HSP90AA1	chr14:102605587-102606086:-	1
3320:i2:-	3320	HSP90AA1	chr14:102568212-102568422:-	1
10808:i1:-	10808	HSPH1	chr13:31736119-31736502:-	-1
285148:i10:+	285148	IAH1	chr2:9636444-9636672:+	-1
57560:i1:-	57560	IFT80	chr3:160167505-160167626:-	1
259307:i3:-	259307	IL4I1	chr19:50430951-50431072:-	-1
8821:i8:-	8821	INPP4B	chr4:143350326-143350370:-	-1
51763:i2:-	51763	INPP5K	chr17:1419182-1419412:-	1
79711:i1:-	79711	IPO4	chr14:24659586-24659859:-	-1
79711:i2:-	79711	IPO4	chr14:24657925-24659014:-	-1
84223:i13:-	84223	IQCG	chr3:197639546-197639620:-	1
84223:i14:-	84223	IQCG	chr3:197619499-197619630:-	1
84223:i15:-	84223	IQCG	chr3:197618317-197618418:-	1
84223:i16:-	84223	IQCG	chr3:197615946-197616585:-	1
51015:i1:+	51015	ISOC1	chr5:128430442-128430768:+	1
3683:i3:+	3683	ITGAL	chr16:30485517-30485619:+	1
3683:i4:+	3683	ITGAL	chr16:30486627-30486721:+	1
3683:i5:+	3683	ITGAL	chr16:30486834-30486901:+	1
23421:i11:-	23421	ITGB3BP	chr1:63913236-63913285:-	1
3691:i42:+	3691	ITGB4	chr17:73752785-73752940:+	1
6453:i45:+	6453	ITSN1	chr21:35260456-35261609:+	-1

8645:i5:-	8645	KCNK5	chr6:39156747-39159531:-	1
3778:i1:-	3778	KCNMA1	chr10:79397023-79397577:-	1
84678:i18:-	84678	KDM2B	chr12:121890923-121891147:-	1
84678:i20:-	84678	KDM2B	chr12:121882253-121882339:-	1
84678:i21:-	84678	KDM2B	chr12:121881815-121882084:-	1
84678:i23:-	84678	KDM2B	chr12:121881483-121881596:-	1
84678:i25:-	84678	KDM2B	chr12:121879960-121880639:-	1
84678:i26:-	84678	KDM2B	chr12:121878873-121879036:-	1
84678:i27:-	84678	KDM2B	chr12:121878619-121878780:-	1
84678:i28:-	84678	KDM2B	chr12:121877660-121877878:-	1
653319:i2:-	653319	KIAA0895L	chr16:67215461-67215600:-	-1
56243:i13:+	56243	KIAA1217	chr10:24783429-24783533:+	-1
56243:i6:+	56243	KIAA1217	chr10:24544257-24544525:+	1
85379:i1:+	85379	KIAA1671	chr22:25423941-25425507:+	-1
85379:i3:+	85379	KIAA1671	chr22:25434727-25437492:+	-1
10749:i23:+	10749	KIF1C	chr17:4926763-4931694:+	1
64147:i1:-	64147	KIF9	chr3:47323985-47324337:-	1
64147:i2:-	64147	KIF9	chr3:47323630-47323875:-	1
54800:i2:+	54800	KLHL24	chr3:183354009-183354099:+	1
3866:i1:-	3866	KRT15	chr17:39677937-39678026:-	-1
79036:i3:+	79036	KXD1	chr19:18671230-18671360:+	-1
91133:i3:-	91133	L3MBTL4	chr18:6311997-6312055:-	1
3909:i10:+	3909	LAMA3	chr18:21355756-21355887:+	-1
3913:i31:-	3913	LAMB2	chr3:49159377-49159518:-	1
143903:i1:+	143903	LAYN	chr11:111411233-111411653:+	1
8861:i2:-	8861	LDB1	chr10:103874163-103874723:-	1
51474:i2:-	51474	LIMA1	chr12:50642416-50642557:-	-1
54923:i1:+	54923	LIME1	chr20:62367053-62367538:+	-1
644873:i4:-	644873	LINC01184	chr5:127357244-127359990:-	-1
29995:i5:+	29995	LMCD1	chr3:8591560-8592961:+	-1
64788:i3:-	64788	LMF1	chr16:1025761-1027093:-	1
84823:i12:-	84823	LMNB2	chr19:2428163-2430950:-	1
84859:i16:+	84859	LRCH3	chr3:197585705-197585776:+	1
9209:i20:-	9209	LRRFIP2	chr3:37132958-37133029:-	1
4052:i1:+	4052	LTBP1	chr2:33172369-33172885:+	1
4052:i2:+	4052	LTBP1	chr2:33173942-33174012:+	1
10296:i8:+	10296	MAEA	chr4:1322422-1323064:+	-1
10916:i2:+	10916	MAGED2	chrX:54834797-54834866:+	1
9794:i6:+	9794	MAML1	chr5:179218765-179223512:+	-1
5606:i4:+	5606	MAP2K3	chr17:21199398-21199522:+	-1
79649:i22:-	79649	MAP7D3	chrX:135295379-135297172:-	-1
10982:i3:+	10982	MAPRE2	chr18:32585456-32585517:+	1
2011:i2:+	2011	MARK2	chr11:63655987-63656436:+	1
375449:i2:+	375449	MAST4	chr5:66055537-66055690:+	-1
10150:i13:+	10150	MBNL2	chr13:98017390-98017425:+	-1
23263:i3:+	23263	MCF2L	chr13:113633655-113634072:+	-1
4176:i2:-	4176	MCM7	chr7:99697836-99698380:-	-1
51586:i3:+	51586	MED15	chr22:20862339-20862731:+	-1
9441:i6:-	9441	MED26	chr19:16677343-16677468:-	-1
9441:i7:-	9441	MED26	chr19:16664581-16664720:-	-1
80306:i6:+	80306	MED28	chr4:17627279-17627468:+	-1
112950:i11:-	112950	MED8	chr1:43849579-43850223:-	-1
151194:i7:-	151194	METTL21A	chr2:208446077-208446884:-	-1
79828:i2:-	79828	METTL8	chr2:172290393-172290619:-	1
4241:i17:-	4241	MFI2	chr3:196730772-196730970:-	-1
4245:i3:-	4245	MGAT1	chr5:180236634-180236817:-	-1
100529262:i2:+	100529262	MIA-RAB4B	chr19:41281442-41281574:+	1
100529262:i3:+	100529262	MIA-RAB4B	chr19:41281657-41281790:+	1
100529262:i4:+	100529262	MIA-RAB4B	chr19:41282874-41282984:+	1

440574:i3:+	440574	MINOS1	chr1:19943752-19943830:+	1
57496:i2:+	57496	MKL2	chr16:14173147-14173211:+	-1
8079:i1:-	8079	MLF2	chr12:6876307-6876641:-	-1
64598:i1:+	64598	MOSPD3	chr7:100209725-100209864:+	-1
22808:i3:+	22808	MRAS	chr3:138067692-138067874:+	1
727957:i18:+	727957	MROH1	chr8:145267897-145268368:+	1
57504:i21:+	57504	MTA3	chr2:42950029-42950175:+	-1
4524:i1:-	4524	MTHFR	chr1:11865945-11866160:-	1
54893:i19:-	54893	MTMR10	chr15:31238413-31238982:-	1
8897:i20:+	8897	MTMR3	chr22:30419446-30419472:+	1
94025:i3:-	94025	MUC16	chr19:9056173-9077865:-	-1
94025:i5:-	94025	MUC16	chr19:9045564-9050243:-	-1
4601:i1:+	4601	MXI1	chr10:111967363-111967840:+	1
4641:i1:-	4641	MYO1C	chr17:1395707-1396001:-	1
4645:i42:-	4645	MYO5B	chr18:47349156-47352993:-	-1
4646:i29:+	4646	MYO6	chr6:76603648-76604977:+	-1
4646:i31:+	4646	MYO6	chr6:76608090-76608128:+	-1
4649:i1:-	4649	MYO9A	chr15:72410021-72410440:-	1
55191:i11:+	55191	NADSYN1	chr11:71187079-71188484:+	1
135112:i20:+	135112	NCOA7	chr6:126240370-126240577:+	1
135112:i21:+	135112	NCOA7	chr6:126242089-126242214:+	1
135112:i22:+	135112	NCOA7	chr6:126243828-126243980:+	1
9612:i3:-	9612	NCOR2	chr12:125002686-125002840:-	-1
54820:i14:+	54820	NDE1	chr16:15818048-15820208:+	-1
81565:i9:+	81565	NDEL1	chr17:8366638-8366672:+	1
80762:i1:+	80762	NDFIP1	chr5:141488324-141488602:+	1
4706:i1:-	4706	NDUFAB1	chr16:23607444-23607639:-	1
4711:i1:+	4711	NDUFB5	chr3:179322575-179322727:+	1
10276:i3:+	10276	NET1	chr10:5468618-5468684:+	1
4763:i35:+	4763	NF1	chr17:29576973-29579822:+	1
10725:i1:+	10725	NFAT5	chr16:69599869-69600277:+	1
4792:i1:-	4792	NFKBIA	chr14:35873624-35873960:-	1
57224:i1:-	57224	NHSL1	chr6:138892847-138893668:-	-1
51199:i33:-	51199	NIN	chr14:51193951-51194469:-	1
654364:i2:+	654364	NME1-NME2	chr17:49231586-49231805:+	1
4833:i1:+	4833	NME4	chr16:446725-447079:+	-1
140688:i1:-	140688	NOL4L	chr20:31172372-31172875:-	-1
140688:i8:-	140688	NOL4L	chr20:31099150-31099259:-	-1
4898:i6:-	4898	NRD1	chr1:52302041-52302110:-	1
387338:i5:+	387338	NSUN4	chr1:46807508-46809966:+	1
11051:i1:-	11051	NUDT21	chr16:56484999-56485261:-	1
23511:i1:+	23511	NUP188	chr9:131703724-131703850:+	-1
93145:i1:-	93145	OLFM2	chr19:10046980-10047070:-	1
26031:i9:-	26031	OSBPL3	chr7:24902819-24902911:-	-1
5051:i1:-	5051	PAFAH2	chr1:26324516-26324648:-	-1
64773:i1:-	64773	PCED1A	chr20:2821512-2821889:-	1
51449:i1:+	51449	PCYOX1	chr2:70484842-70485067:+	1
10016:i6:+	10016	PDCD6	chr5:276127-277055:+	1
5142:i6:+	5142	PDE4B	chr1:66713143-66713337:+	1
8654:i1:-	8654	PDE5A	chr4:120549675-120549981:-	1
5155:i1:-	5155	PDGFB	chr22:39639906-39640957:-	-1
5209:i1:+	5209	PFKFB3	chr10:6186843-6186976:+	1
5211:i5:+	5211	PFKL	chr21:45725683-45725818:+	-1
65979:i4:+	65979	PHACTR4	chr1:28764661-28764941:+	1
1912:i14:-	1912	PHC2	chr1:33799691-33799893:-	1
26227:i5:+	26227	PHGDH	chr1:120266942-120267092:+	1
254295:i1:+	254295	PHYHD1	chr9:131683174-131683947:+	1
5298:i5:-	5298	PI4KB	chr1:151282687-151282731:-	-1
5295:i1:+	5295	PIK3R1	chr5:67511584-67511777:+	-1

5295:i2:+	5295	PIK3R1	chr5:67522118-67522837:+	-1
5295:i3:+	5295	PIK3R1	chr5:67569218-67569310:+	-1
5295:i4:+	5295	PIK3R1	chr5:67569767-67569841:+	-1
5295:i5:+	5295	PIK3R1	chr5:67575430-67575561:+	-1
5295:i6:+	5295	PIK3R1	chr5:67576356-67576557:+	-1
5295:i7:+	5295	PIK3R1	chr5:67576755-67576834:+	-1
23228:i3:+	23228	PLCL2	chr3:16926563-16926865:+	1
440456:i17:-	440456	PLEKHM1P	chr17:62776461-62777157:-	1
440456:i18:-	440456	PLEKHM1P	chr17:62776099-62776323:-	1
5376:i1:-	5376	PMP22	chr17:15168471-15168644:-	-1
5376:i2:-	5376	PMP22	chr17:15165746-15165889:-	1
5382:i1:-	5382	PMS2P4	chr7:66767363-66767429:-	-1
692312:i14:+	692312	PPAN-P2RY11	chr19:10224309-10226064:+	-1
53938:i6:-	53938	PPIL3	chr2:201747065-201747158:-	-1
5524:i14:+	5524	PPP2R4	chr9:131902209-131902430:+	-1
5524:i15:+	5524	PPP2R4	chr9:131903935-131904197:+	1
5537:i9:-	5537	PPP6C	chr9:127908852-127912200:-	-1
51422:i1:-	51422	PRKAG2	chr7:151573592-151574316:-	-1
51422:i4:-	51422	PRKAG2	chr7:151478238-151478517:-	-1
51422:i7:-	51422	PRKAG2	chr7:151372025-151372723:-	-1
5578:i19:+	5578	PRKCA	chr17:64799991-64806862:+	-1
8575:i1:-	8575	PRKRA	chr2:179315693-179315958:-	1
339105:i2:-	339105	PRSS53	chr16:31104633-31104742:-	-1
5682:i17:-	5682	PSMA1	chr11:14515756-14515985:-	-1
5682:i18:-	5682	PSMA1	chr11:14515188-14515357:-	-1
5792:i17:+	5792	PTPRF	chr1:44067742-44067768:+	1
11122:i33:-	11122	PTPRT	chr20:40701392-40709572:-	1
5829:i12:-	5829	PXN	chr12:120653363-120653464:-	1
57403:i1:+	57403	RAB22A	chr20:56884771-56885068:+	1
83871:i2:-	83871	RAB34	chr17:27044231-27044908:-	1
10567:i1:-	10567	RABAC1	chr19:42463389-42463528:-	-1
55684:i6:+	55684	RABL6	chr9:139722234-139722316:+	-1
5879:i5:+	5879	RAC1	chr7:6438293-6438349:+	-1
55698:i4:-	55698	RADIL	chr7:4875989-4876236:-	1
55698:i5:-	55698	RADIL	chr7:4874238-4874870:-	1
5898:i5:+	5898	RALA	chr7:39745722-39747723:+	-1
253959:i45:-	253959	RALGAPA1	chr14:36017714-36017744:-	1
5900:i1:-	5900	RALGDS	chr9:136039141-136039301:-	1
5902:i1:+	5902	RANBP1	chr22:20105024-20105187:+	1
2889:i17:-	2889	RAPGEF1	chr9:134479348-134479440:-	1
100271927:i6:-	100271927	RASA4B	chr7:102183972-102184517:-	1
158158:i2:-	158158	RASEF	chr9:85669575-85670598:-	1
11186:i1:-	11186	RASSF1	chr3:50377987-50378367:-	1
5931:i2:-	5931	RBBP7	chrX:16887617-16887978:-	1
64080:i1:-	64080	RBKS	chr2:28113124-28113223:-	1
58517:i13:+	58517	RBM25	chr14:73567422-73567884:+	-1
54502:i1:-	54502	RBM47	chr4:40632480-40632640:-	1
10180:i24:+	10180	RBM6	chr3:50127815-50127884:+	-1
10180:i25:+	10180	RBM6	chr3:50129476-50129641:+	-1
10180:i26:+	10180	RBM6	chr3:50131153-50131308:+	-1
10180:i27:+	10180	RBM6	chr3:50137415-50137484:+	-1
5947:i4:-	5947	RBP1	chr3:139245247-139246184:-	-1
55758:i1:+	55758	RCOR3	chr1:211432708-211433068:+	1
8490:i1:-	8490	RGS5	chr1:163291412-163291581:-	1
8490:i2:-	8490	RGS5	chr1:163288972-163289081:-	1
55183:i45:+	55183	RIF1	chr2:152362724-152362889:+	1
57494:i3:+	57494	RIMKLB	chr12:8852381-8853210:+	1
51115:i11:-	51115	RMDN1	chr8:87484578-87486587:-	-1
8635:i2:-	8635	RNASET2	chr6:167369585-167370077:-	1

100526767:i3:-	100526767	RNF103-CHMP3	chr2:86847453-86847592:-	-1
11342:i2:+	11342	RNF13	chr3:149531712-149531813:+	1
80196:i2:+	80196	RNF34	chr12:121840545-121840610:+	1
84900:i2:+	84900	RNFT2	chr12:117178150-117178326:+	-1
100526842:i8:-	100526842	RPL17-C18orf32	chr18:47007548-47008780:-	-1
6143:i1:+	6143	RPL19	chr17:37356536-37356602:+	1
6132:i2:-	6132	RPL8	chr8:146017630-146017729:-	1
6210:i2:-	6210	RPS15A	chr16:18801566-18801656:-	1
58528:i1:-	58528	RRAGD	chr6:90121565-90121995:-	1
51750:i39:+	51750	RTEL1	chr20:62327131-62328544:+	1
100533107:i40:+	100533107	RTEL1-TNFRSF6B	chr20:62328113-62328544:+	1
100533107:i42:+	100533107	RTEL1-TNFRSF6B	chr20:62329633-62330051:+	1
10313:i3:+	10313	RTN3	chr11:63486174-63488504:+	1
146923:i1:+	146923	RUNDC1	chr17:41132582-41133091:+	1
6256:i6:+	6256	RXRA	chr9:137298428-137299338:+	-1
140700:i1:-	140700	SAMD10	chr20:62611207-62611362:-	-1
22955:i16:-	22955	SCMH1	chr1:41503034-41503213:-	1
10806:i2:+	10806	SDCCAG8	chr1:243433407-243433559:+	-1
10806:i3:+	10806	SDCCAG8	chr1:243434280-243434365:+	-1
10806:i4:+	10806	SDCCAG8	chr1:243437845-243437958:+	-1
10806:i5:+	10806	SDCCAG8	chr1:243449574-243449699:+	-1
10806:i6:+	10806	SDCCAG8	chr1:243456393-243456521:+	-1
10806:i8:+	10806	SDCCAG8	chr1:243468015-243468079:+	-1
9919:i38:-	9919	SEC16A	chr9:139339504-139339563:-	1
9117:i4:-	9117	SEC22C	chr3:42605257-42605372:-	1
22872:i32:-	22872	SEC31A	chr4:83752090-83752128:-	-1
51091:i2:-	51091	SEPSECS	chr4:25161250-25161386:-	1
641977:i1:-	641977	SEPT7P2	chr7:45808259-45808617:-	1
10801:i2:+	10801	SEPT9	chr17:75283973-75284292:+	-1
26135:i10:-	26135	SERBP1	chr1:67873493-67878946:-	-1
12:i1:+	12	SERPINA3	chr14:95058395-95058473:+	-1
27244:i1:-	27244	SESN1	chr6:109414998-109415708:-	1
23064:i3:-	23064	SETX	chr9:135224639-135224822:-	-1
6446:i6:-	6446	SGK1	chr6:134496682-134497070:-	1
63898:i1:+	63898	SH2D4A	chr8:19171081-19171334:+	1
23677:i2:+	23677	SH3BP4	chr2:235887329-235887629:+	-1
79729:i13:+	79729	SH3D21	chr1:36784861-36784913:+	-1
92799:i21:+	92799	SHKBP1	chr19:41096636-41096759:+	1
92799:i22:+	92799	SHKBP1	chr19:41096882-41097305:+	1
6472:i2:+	6472	SHMT2	chr12:57623828-57624027:+	1
51547:i1:-	51547	SIRT7	chr17:79875915-79876058:-	-1
84561:i1:-	84561	SLC12A8	chr3:124997968-124998091:-	-1
84561:i2:-	84561	SLC12A8	chr3:124996570-124996653:-	-1
64849:i1:-	64849	SLC13A3	chr20:45313035-45313124:-	-1
9016:i3:+	9016	SLC25A14	chrX:129474047-129474327:+	1
115286:i16:+	115286	SLC25A26	chr3:66436608-66438532:+	-1
6533:i13:+	6533	SLC6A6	chr3:14509596-14509720:+	-1
23428:i1:-	23428	SLC7A8	chr14:23651973-23652869:-	-1
23428:i2:-	23428	SLC7A8	chr14:23635545-23635749:-	-1
23428:i5:-	23428	SLC7A8	chr14:23623468-23623659:-	1
7871:i27:+	7871	SLMAP	chr3:57911572-57911661:+	1
27127:i26:-	27127	SMC1B	chr22:45739945-45740538:-	-1
23161:i8:-	23161	SNX13	chr7:17919053-17922566:-	1
6651:i14:+	6651	SON	chr21:34944675-34944936:+	1
10580:i11:-	10580	SORBS1	chr10:97175226-97175942:-	1
10174:i20:+	10174	SORBS3	chr8:22429231-22429334:+	1
202051:i6:-	202051	SPATA24	chr5:138732730-138732832:-	-1
202051:i8:-	202051	SPATA24	chr5:138732255-138732644:-	-1
25803:i1:-	25803	SPDEF	chr6:34523706-34524110:-	-1

6709:i2:+	6709	SPTAN1	chr9:131315360-131315615:+	1
9517:i12:-	9517	SPTLC2	chr14:77972340-77978746:-	-1
10847:i30:+	10847	SRCAP	chr16:30744923-30745119:+	1
10847:i32:+	10847	SRCAP	chr16:30745817-30745936:+	1
10847:i33:+	10847	SRCAP	chr16:30747521-30747715:+	1
10847:i34:+	10847	SRCAP	chr16:30747862-30747945:+	1
10847:i35:+	10847	SRCAP	chr16:30748370-30752028:+	1
6720:i1:-	6720	SREBF1	chr17:17740041-17740325:-	1
6717:i13:-	6717	SRI	chr7:87834432-87835819:-	-1
23524:i13:+	23524	SRRM2	chr16:2818998-2819285:+	1
23524:i14:+	23524	SRRM2	chr16:2820353-2820466:+	1
23524:i15:+	23524	SRRM2	chr16:2820605-2820718:+	1
23524:i16:+	23524	SRRM2	chr16:2820859-2821413:+	1
6432:i4:-	6432	SRSF7	chr2:38976040-38976488:-	-1
6741:i2:+	6741	SSB	chr2:170655789-170656026:+	1
23648:i6:-	23648	SSBP3	chr1:54723742-54723822:-	-1
10610:i1:-	10610	ST6GALNAC2	chr17:74581766-74582145:-	1
30815:i2:-	30815	ST6GALNAC6	chr9:130661782-130661871:-	-1
7982:i10:+	7982	ST7	chr7:116774178-116774246:+	1
10809:i2:-	10809	STARD10	chr11:72503920-72504750:-	1
6780:i5:-	6780	STAU1	chr20:47782534-47782822:-	1
11171:i10:+	11171	STRAP	chr12:16053881-16053946:+	1
8675:i7:+	8675	STX16	chr20:57234679-57234690:+	-1
2054:i2:-	2054	STX2	chr12:131311738-131311812:-	1
100130958:i1:+	100130958	SYCE1L	chr16:77233349-77233409:+	-1
94121:i4:-	94121	SYTL4	chrX:99958089-99958181:-	-1
94122:i19:+	94122	SYTL5	chrX:37985841-37988073:+	1
10454:i1:+	10454	TAB1	chr22:39745954-39746114:+	1
79101:i2:-	79101	TAF1D	chr11:93472403-93472497:-	-1
10010:i1:+	10010	TANK	chr2:161993466-161993574:+	1
23216:i3:+	23216	TBC1D1	chr4:37903624-37904133:+	-1
23232:i1:+	23232	TBC1D12	chr10:96162186-96163341:+	1
57533:i13:+	57533	TBC1D14	chr4:7009251-7009352:+	-1
57533:i18:+	57533	TBC1D14	chr4:7032054-7034845:+	1
23158:i1:-	23158	TBC1D9	chr4:141677070-141677471:-	1
93627:i1:-	93627	TBCK	chr4:107237705-107237861:-	1
6907:i6:+	6907	TBL1X	chrX:9621585-9621729:+	-1
6919:i3:+	6919	TCEA2	chr20:62688439-62689004:+	1
6938:i20:+	6938	TCF12	chr15:57544397-57544690:+	-1
55775:i25:+	55775	TDP1	chr14:90509414-90511108:+	-1
8463:i4:-	8463	TEAD2	chr19:49864653-49864752:-	-1
7027:i3:+	7027	TFDP1	chr13:114239588-114239742:+	1
7942:i6:-	7942	TFEB	chr6:41700387-41701591:-	-1
51078:i3:-	51078	THAP4	chr2:242556827-242556916:-	-1
9414:i5:+	9414	TJP2	chr9:71788971-71789348:+	1
7086:i21:-	7086	TKT	chr3:53258723-53259625:-	1
89894:i1:-	89894	TMEM116	chr12:112450806-112451023:-	1
129787:i2:-	129787	TMEM18	chr2:675758-676158:-	1
387522:i6:-	387522	TMEM189-UBE2V1	chr20:48713209-48713357:-	-1
387522:i7:-	387522	TMEM189-UBE2V1	chr20:48700666-48700791:-	-1
387522:i8:-	387522	TMEM189-UBE2V1	chr20:48697661-48699451:-	-1
85019:i15:-	85019	TMEM241	chr18:20910994-20911223:-	-1
85019:i16:-	85019	TMEM241	chr18:20889644-20889708:-	-1
85019:i17:-	85019	TMEM241	chr18:20875979-20878031:-	-1
80195:i2:+	80195	TMEM254	chr10:81838779-81838940:+	1
100529211:i1:-	100529211	TMEM256-PLSCR3	chr17:7307319-7307450:-	-1
100529211:i2:-	100529211	TMEM256-PLSCR3	chr17:7306977-7307008:-	-1
100529211:i3:-	100529211	TMEM256-PLSCR3	chr17:7306629-7306709:-	-1
120224:i7:+	120224	TMEM45B	chr11:129728469-129729898:+	1

164656:i21:-	164656	TMPRSS6	chr22:37461479-37462278:-	1
7127:i1:+	7127	TNFAIP2	chr14:103592664-103593029:+	-1
55504:i1:+	55504	TNFRSF19	chr13:24144509-24144987:+	-1
30000:i2:-	30000	TNPO2	chr19:12834063-12834179:-	1
23112:i4:+	23112	TNRC6B	chr22:40521776-40521866:+	-1
9537:i6:-	9537	TP53I11	chr11:44962065-44962298:-	-1
7163:i3:-	7163	TPD52	chr8:80992550-80993010:-	-1
7170:i1:-	7170	TPM3	chr1:154164378-154164611:-	-1
7170:i3:-	7170	TPM3	chr1:154163662-154163787:-	-1
285386:i7:+	285386	TPRG1	chr3:188889763-188889920:+	-1
80342:i11:+	80342	TRAF3IP3	chr1:209946293-209946364:+	1
80342:i15:+	80342	TRAF3IP3	chr1:209951456-209951518:+	1
147166:i10:+	147166	TRIM16L	chr17:18630838-18631071:+	1
147166:i13:+	147166	TRIM16L	chr17:18638190-18639431:+	1
23321:i2:+	23321	TRIM2	chr4:154125598-154125712:+	1
51592:i13:-	51592	TRIM33	chr1:114963002-114963073:-	-1
11078:i17:+	11078	TRIOBP	chr22:38130406-38131449:+	1
7227:i2:-	7227	TRPS1	chr8:116680101-116680234:-	1
1831:i6:-	1831	TSC22D3	chrX:106959545-106959711:-	1
100303453:i3:+	100303453	TSNAX-DISC1	chr1:231672959-231673073:+	-1
100303453:i4:+	100303453	TSNAX-DISC1	chr1:231678227-231678357:+	-1
100303453:i5:+	100303453	TSNAX-DISC1	chr1:231696874-231697001:+	-1
125488:i2:+	125488	TTC39C	chr18:21594384-21595002:+	1
57217:i1:+	57217	TTC7A	chr2:47143296-47143716:+	-1
57217:i7:+	57217	TTC7A	chr2:47185634-47185691:+	-1
10426:i1:-	10426	TUBGCP3	chr13:113242219-113242481:-	1
10426:i2:-	10426	TUBGCP3	chr13:113223466-113223573:-	1
10426:i5:-	10426	TUBGCP3	chr13:113212510-113212727:-	1
10426:i7:-	10426	TUBGCP3	chr13:113210366-113210538:-	1
10426:i10:-	10426	TUBGCP3	chr13:113202422-113202490:-	1
10426:i12:-	10426	TUBGCP3	chr13:113201875-113202066:-	1
7991:i11:+	7991	TUSC3	chr8:15621712-15624158:+	-1
55075:i2:-	55075	UACA	chr15:70994216-70994620:-	-1
7323:i1:-	7323	UBE2D3	chr4:103789872-103790032:-	1
7327:i2:-	7327	UBE2G2	chr21:46221199-46221313:-	1
23352:i100:-	23352	UBR4	chr1:19422039-19422146:-	1
23352:i101:-	23352	UBR4	chr1:19421400-19421519:-	1
23352:i105:-	23352	UBR4	chr1:19415253-19415471:-	1
23352:i107:-	23352	UBR4	chr1:19413173-19413315:-	1
23352:i108:-	23352	UBR4	chr1:19412639-19412764:-	1
23352:i110:-	23352	UBR4	chr1:19407843-19408140:-	1
23352:i112:-	23352	UBR4	chr1:19404470-19404560:-	1
7343:i1:-	7343	UBTF	chr17:42298569-42298994:-	-1
7360:i2:+	7360	UGP2	chr2:64069014-64069338:+	1
127933:i2:+	127933	UHMK1	chr1:162467595-162468058:+	1
7374:i1:+	7374	UNG	chr12:109535399-109535616:+	1
64718:i13:-	64718	UNKL	chr16:1429534-1429684:-	1
7381:i6:-	7381	UQCRB	chr8:97243454-97243743:-	1
8615:i15:+	8615	USO1	chr4:76716489-76716509:+	1
27005:i2:+	27005	USP21	chr1:161130156-161130296:+	-1
57478:i1:-	57478	USP31	chr16:23159959-23160591:-	1
84132:i18:+	84132	USP42	chr7:6193387-6194826:+	1
84132:i20:+	84132	USP42	chr7:6196385-6196962:+	1
27340:i61:+	27340	UTP20	chr12:101777321-101777447:+	1
1462:i8:+	1462	VCAN	chr5:82815168-82818128:+	-1
137492:i14:+	137492	VPS37A	chr8:17152496-17155533:+	-1
143187:i2:+	143187	VTI1A	chr10:114208640-114210483:+	1
4013:i2:+	4013	VWA5A	chr11:123987273-123987387:+	1
7450:i56:-	7450	VWF	chr12:6058040-6058369:-	1

84292:i1:+	84292	WDR83	chr19:12777618-12777806:+	-1
7466:i1:+	7466	WFS1	chr4:6271577-6271741:+	1
58525:i2:-	58525	WIZ	chr19:15558914-15559178:-	-1
58525:i3:-	58525	WIZ	chr19:15547617-15547940:-	-1
7485:i2:+	7485	WRB	chr21:40759692-40759781:+	1
54432:i2:-	54432	YIPF1	chr1:54354917-54355136:-	1
7534:i5:-	7534	YWHAZ	chr8:101963414-101963560:-	1
23318:i2:-	23318	ZCCHC11	chr1:53018603-53018772:-	1
54877:i1:+	54877	ZCCHC2	chr18:60190658-60191596:+	1
161882:i10:+	161882	ZFPM1	chr16:88599556-88601574:+	1
57178:i4:+	57178	ZMIZ1	chr10:80921810-80921890:+	-1
57178:i5:+	57178	ZMIZ1	chr10:80961341-80961449:+	-1
57178:i6:+	57178	ZMIZ1	chr10:80968093-80968206:+	-1
7753:i1:-	7753	ZNF202	chr11:123612257-123612363:-	1
7753:i3:-	7753	ZNF202	chr11:123611101-123611244:-	1
51222:i1:-	51222	ZNF219	chr14:21572670-21572863:-	1
90987:i1:-	90987	ZNF251	chr8:145980790-145980970:-	1
171017:i1:-	171017	ZNF384	chr12:6798534-6798738:-	1
22869:i2:-	22869	ZNF510	chr9:99539864-99540338:-	1
84503:i5:+	84503	ZNF527	chr19:37875770-37876545:+	1
27300:i10:+	27300	ZNF544	chr19:58762674-58763073:+	-1
148103:i6:-	148103	ZNF599	chr19:35248979-35251464:-	-1
114991:i4:+	114991	ZNF618	chr9:116760539-116760634:+	-1
100131827:i7:-	100131827	ZNF717	chr3:75786029-75788496:-	-1
125893:i3:-	125893	ZNF816	chr19:53463280-53463430:-	-1
TCONSl200001046:i2:-	TCONSl200001046		chr1:16969523-16969640:-	-1
TCONSl200002441:i2:-	TCONSl200002441		chr1:16969523-16969640:-	-1

Supplementary Figure S12
IGV primary data presentation

A MYO6

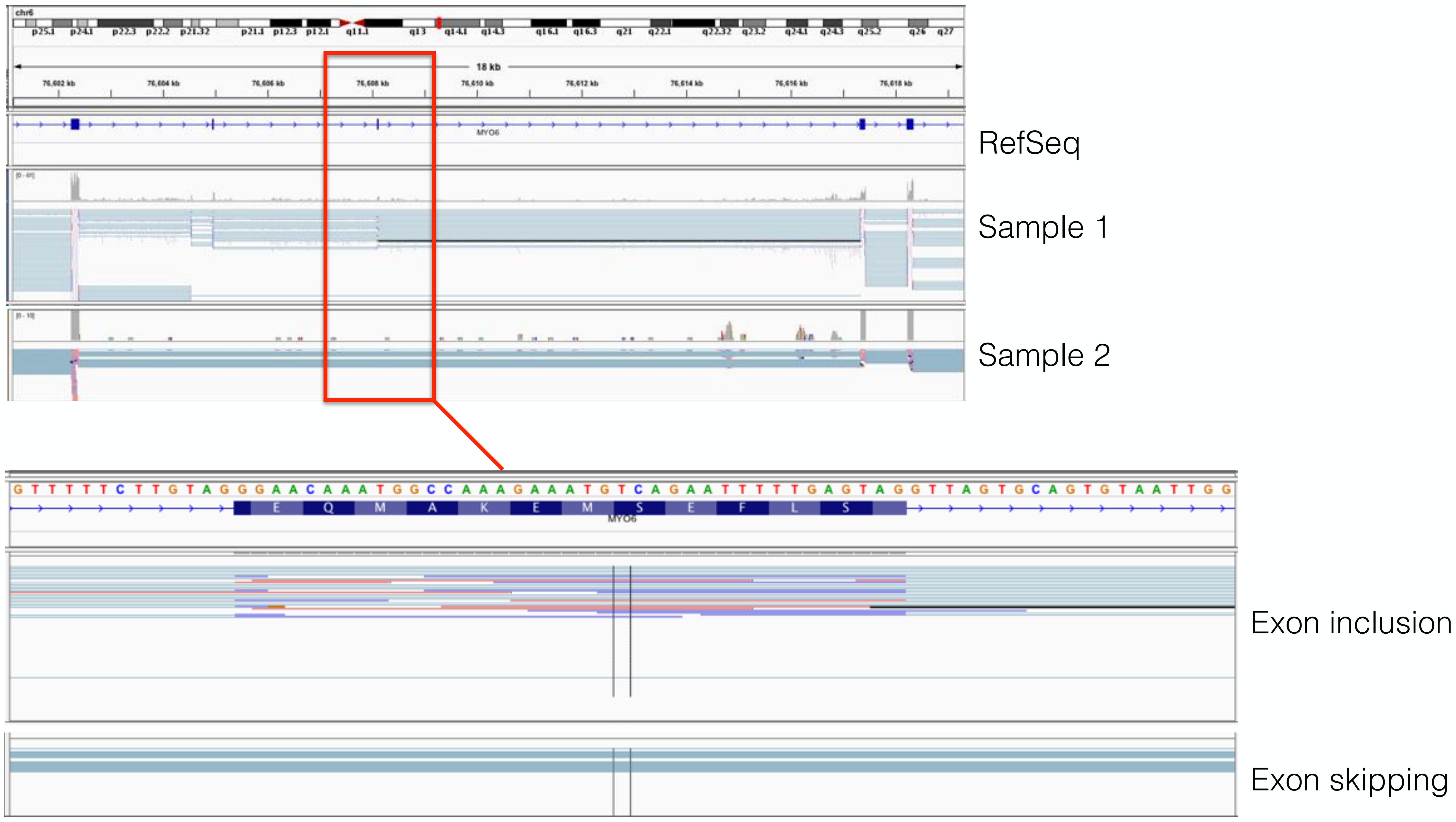

B EPB41L1

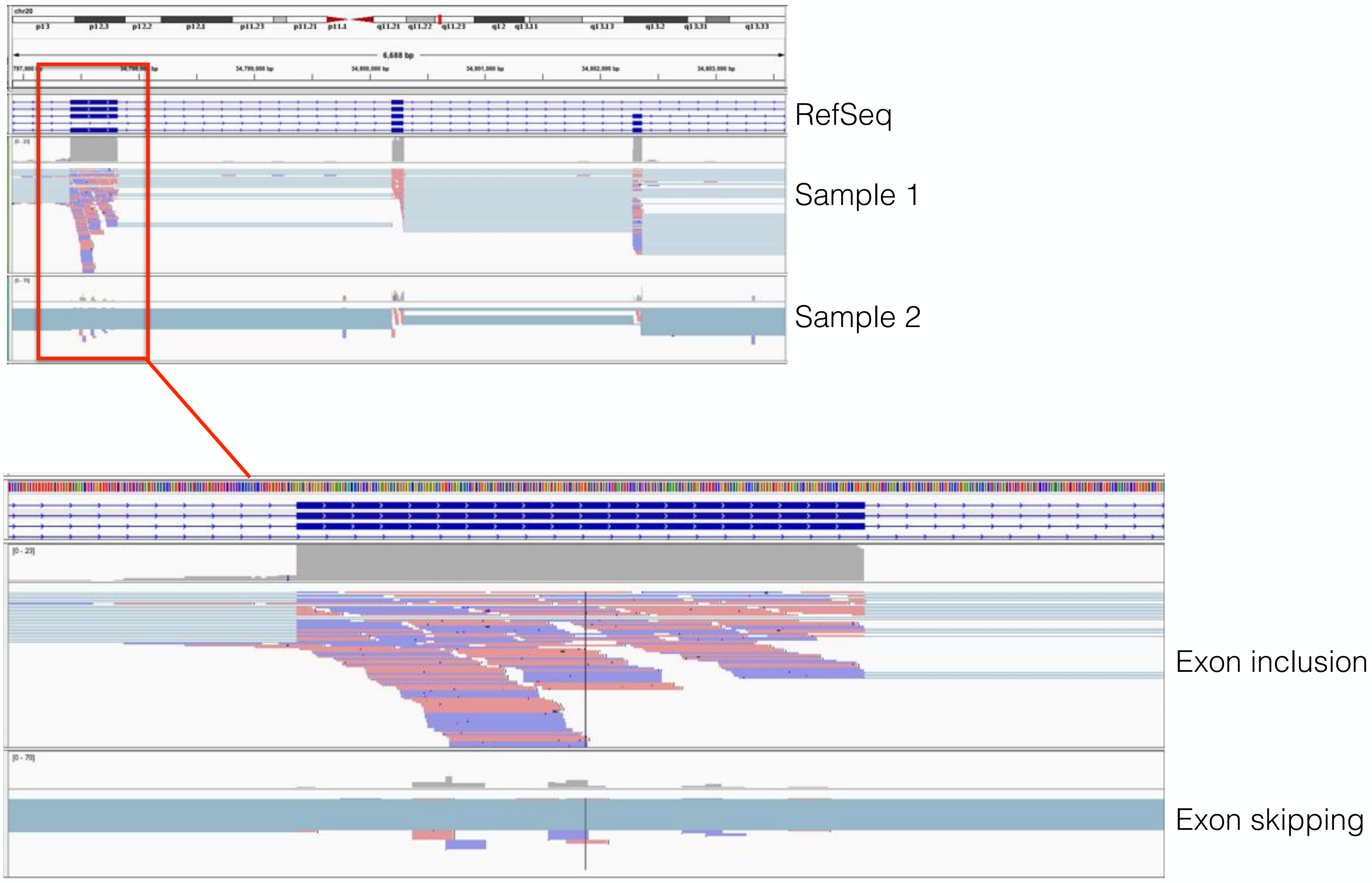

C TPD52

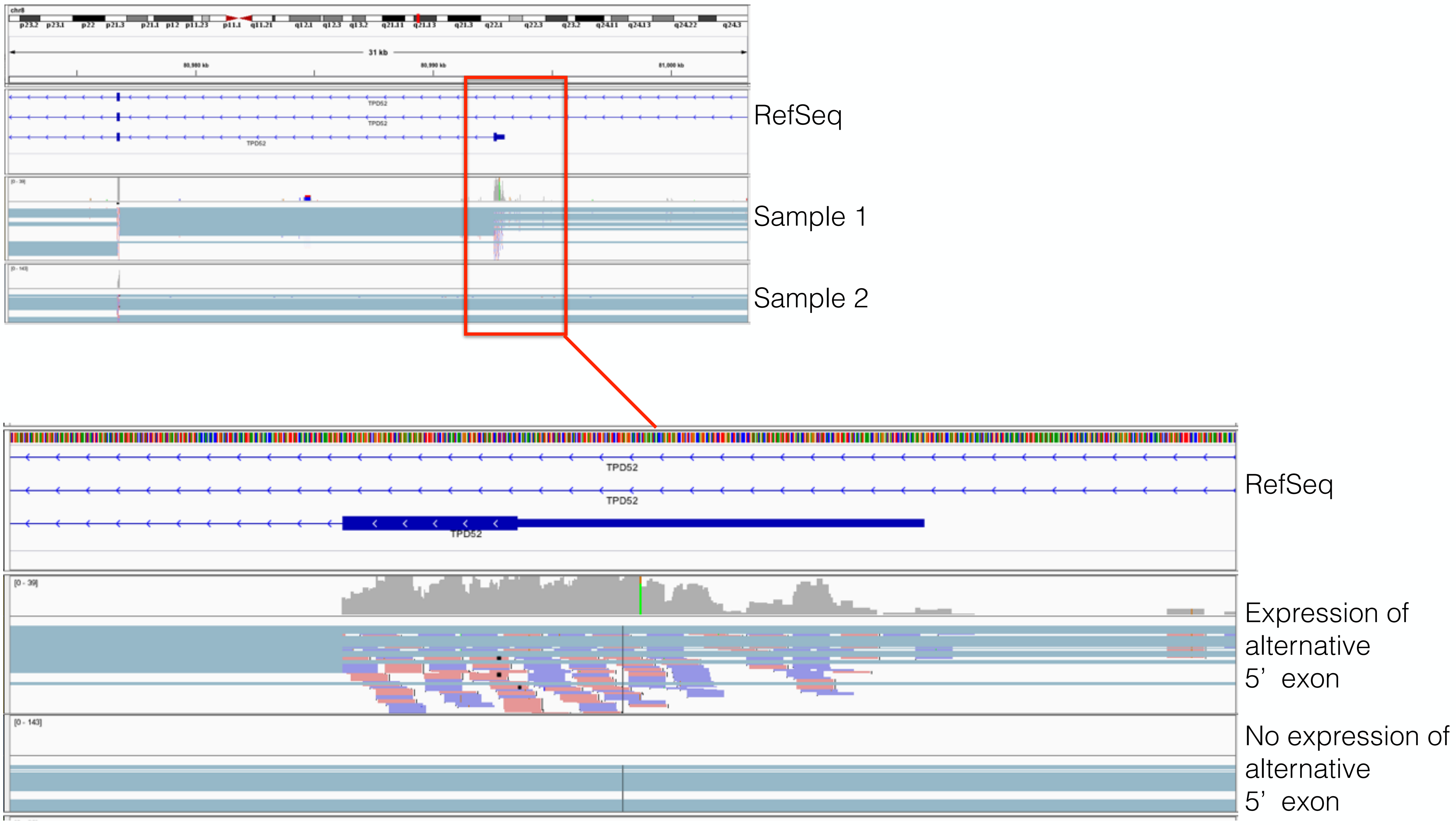

D IQCG

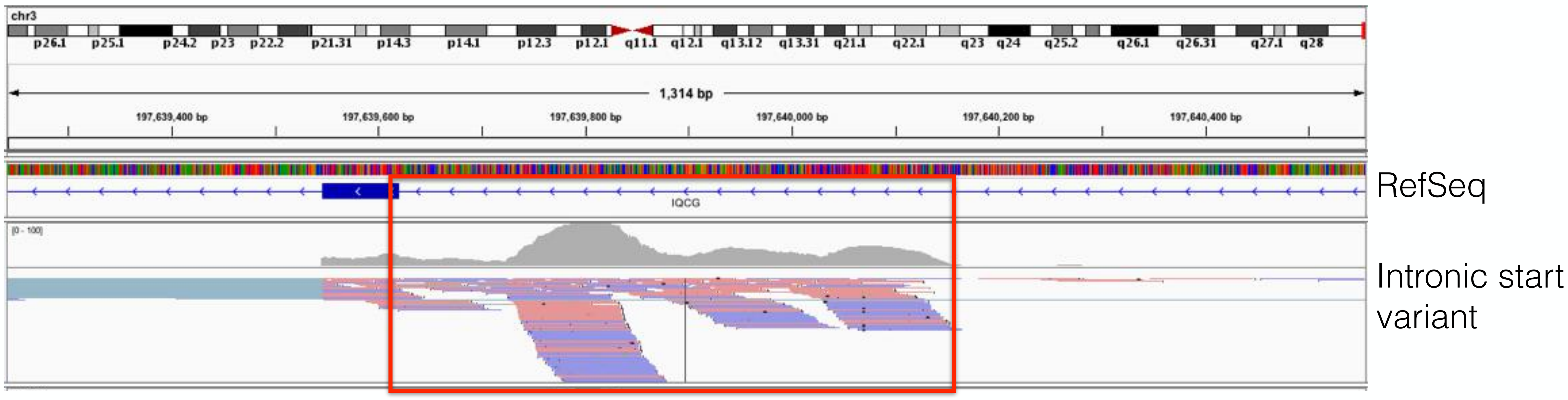

E ACOX2

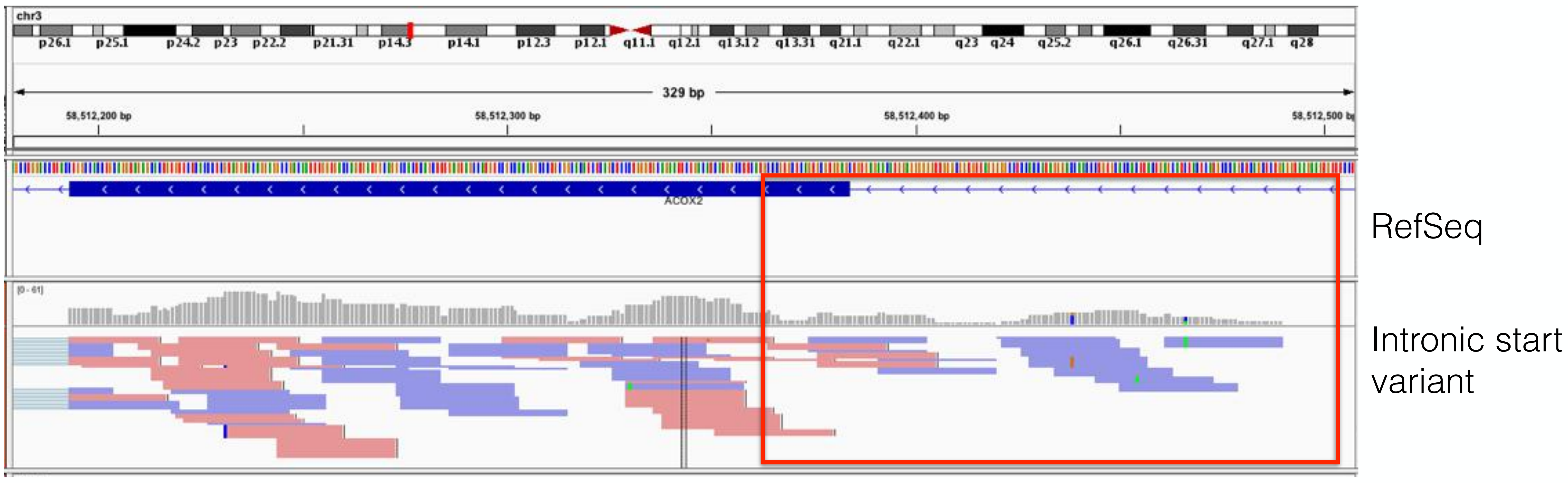

Supplementary Figure S12. *Primary data viewed in IGV.* Coverage and bam files from two (A, B, and C) or one sample (D and E) that illustrate the selected splicing and transcriptional events in MYO6, EPB41L1, TPD52, IQCG, and ACOX2 as viewed in IGV. RefSeq transcripts and Chromosome location are included for reference. A, B, and C all show exon skipping/inclusion events, and samples are included to show both events. Exon inclusion is visible as reads (red and blue) overlapping the exon of interest, whereas exon skipping is shown as thin blue lines (reads mapping to two separate exons) spanning the exon. C and D both include one sample showing reads (red and blue) in the intronic region preceding an exon.

Supplementary Table S13 Taqman probes

gene	short names	pre-designed probe	custom probe	forward_pimer	reverse_primer	probe	NM_ID_detected
TPD52	TPD52_1	Hs00180361_m1					NM_005079, NM_001025253, NM_1287144
TPD52	TPD52_2		PrimerExpress #1	TCCACCCGGATCACCTACTC	TCCTCAGGGACTGGGTCTGT	TCAGAAATTTGGTCTGCTGAG	NM_001025252, NR_105033, NM_001287143, NM_001287142, NM_001287140
ACOX2	ACOX2_2		PrimerExpress #1	CCAACCCCCCTCCATGTT	AATTCTGACATCATGGCCTTCA	CACGCACTGAGCACG	NM_003500_intron9
IQCG	IQCG_2		PrimerExpress Forw #20, Rev #1, probe #1	CGTACTGGTATCTGATTGTTCTTACAGA	CTCAAGTTTCTGCTGCTCCTTTC	CAGAGATTGAAATGTTCC	NM_032263_intron8
MYO6	MYO6_1	Hs01568229_m1					NM_004999.3
MYO6	MYO6_2	Hs01568241_m1					NM_001300899
EPB41L1	EPB41L1_1	Hs00385004_m1					NM_012156, NM_001258329, NM_001258331, NM_177996
EPB41L1	EPB41L1_2		PrimerExpress #8	CCCCTGAGAAAGCCAATGAG	TCTAATGGCCAGACTGCTGACA	CCGTGAAAACAGAAAC	NM_001258330
Endogenous control	PMM1	Hs00963626_m1					
Endogenous control	RPL32	Hs00851655_g1					

Supplementary Figure S14

Taqman validation probe placement and transcripts detected

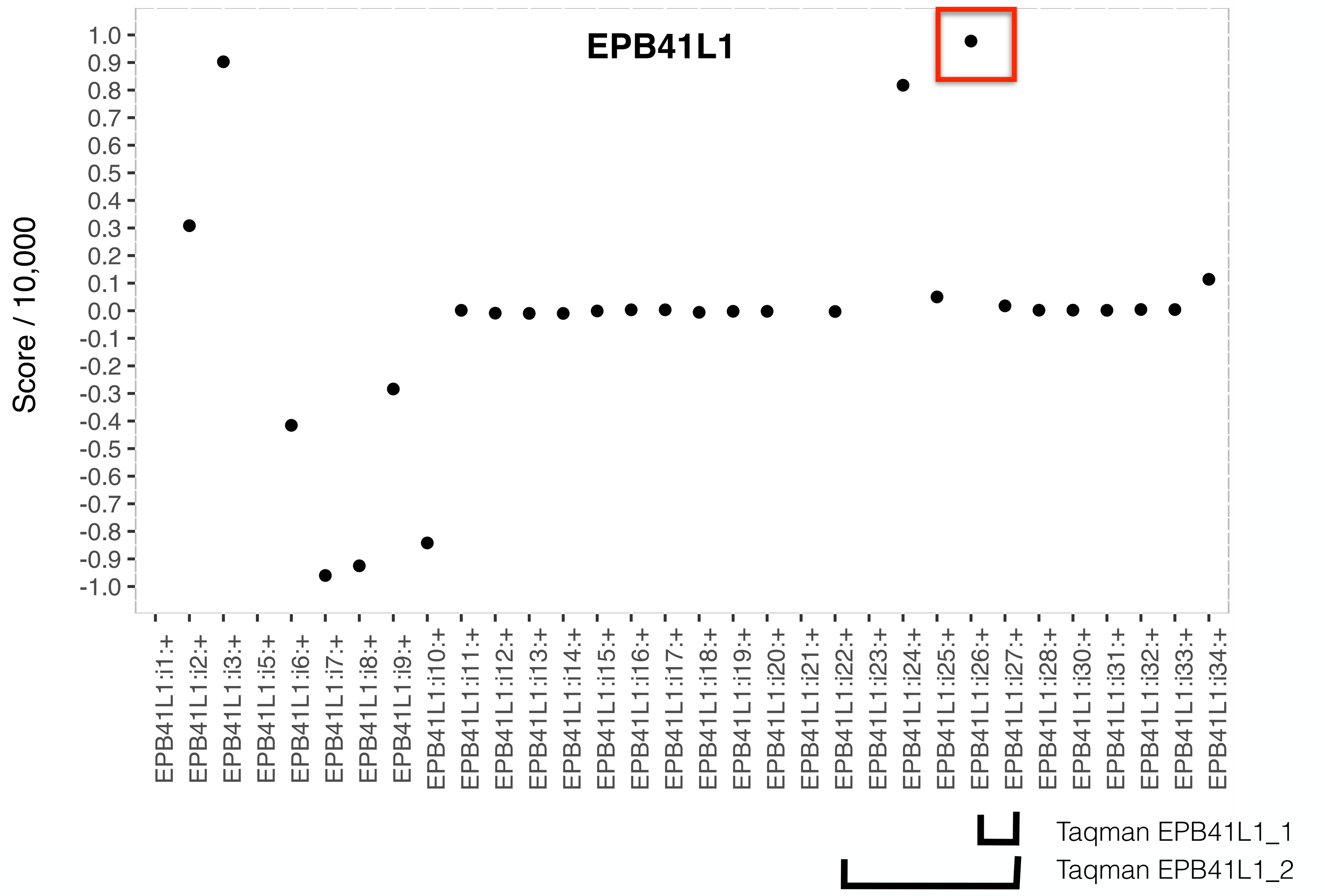

All transcripts detected by EPB41L1_1: ENST00000202028, ENST00000338074, ENST00000441639, ENST00000373950, ENST00000373941

All transcripts detected by EPB41L1_2: ENST00000373946, ENST00000451082, ENST00000454226

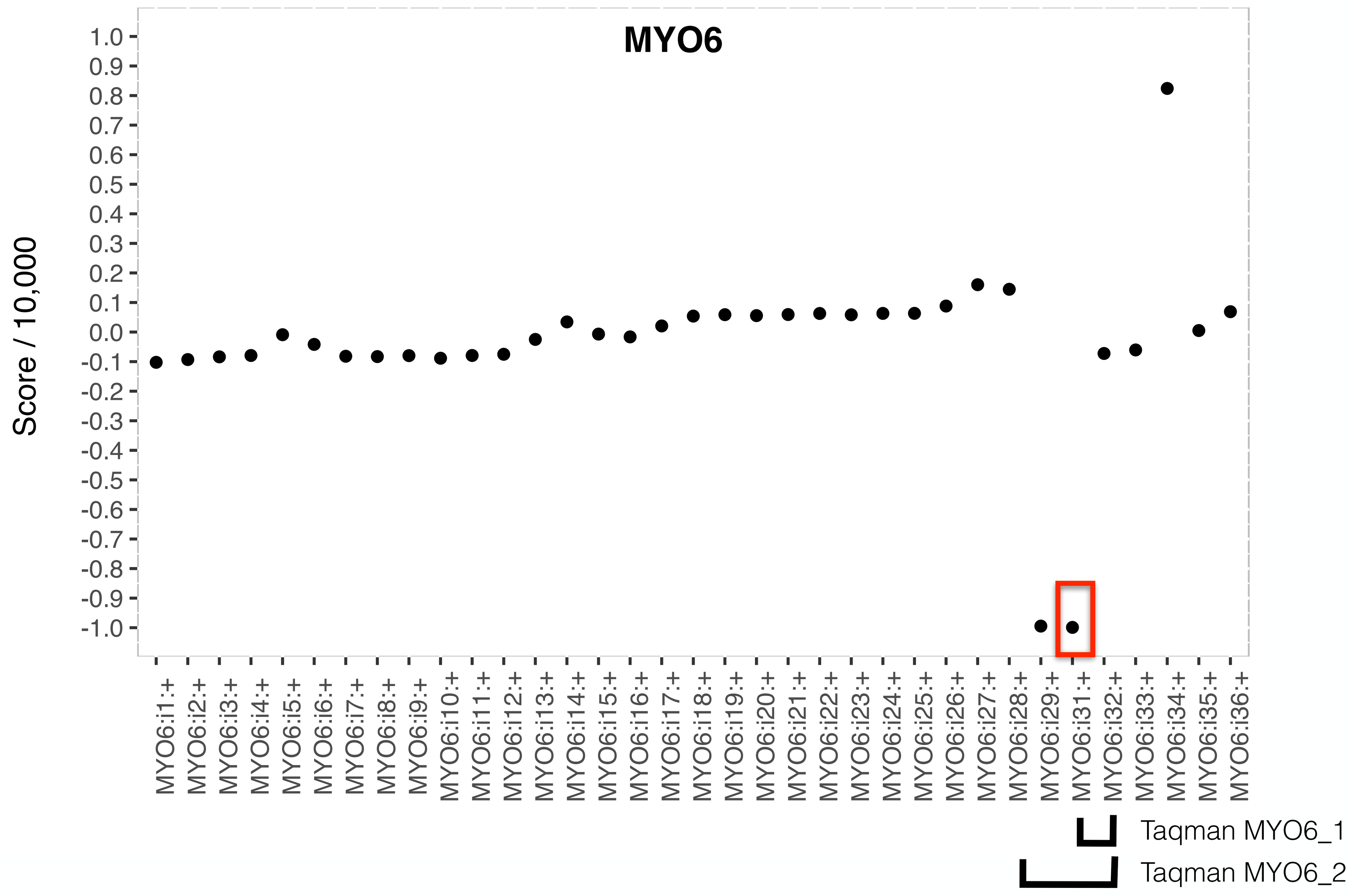

All transcripts detected by MYO6_1: ENST00000369977, ENST00000369981

All transcripts detected by MYO6_2: ENST00000369985, ENST00000369975

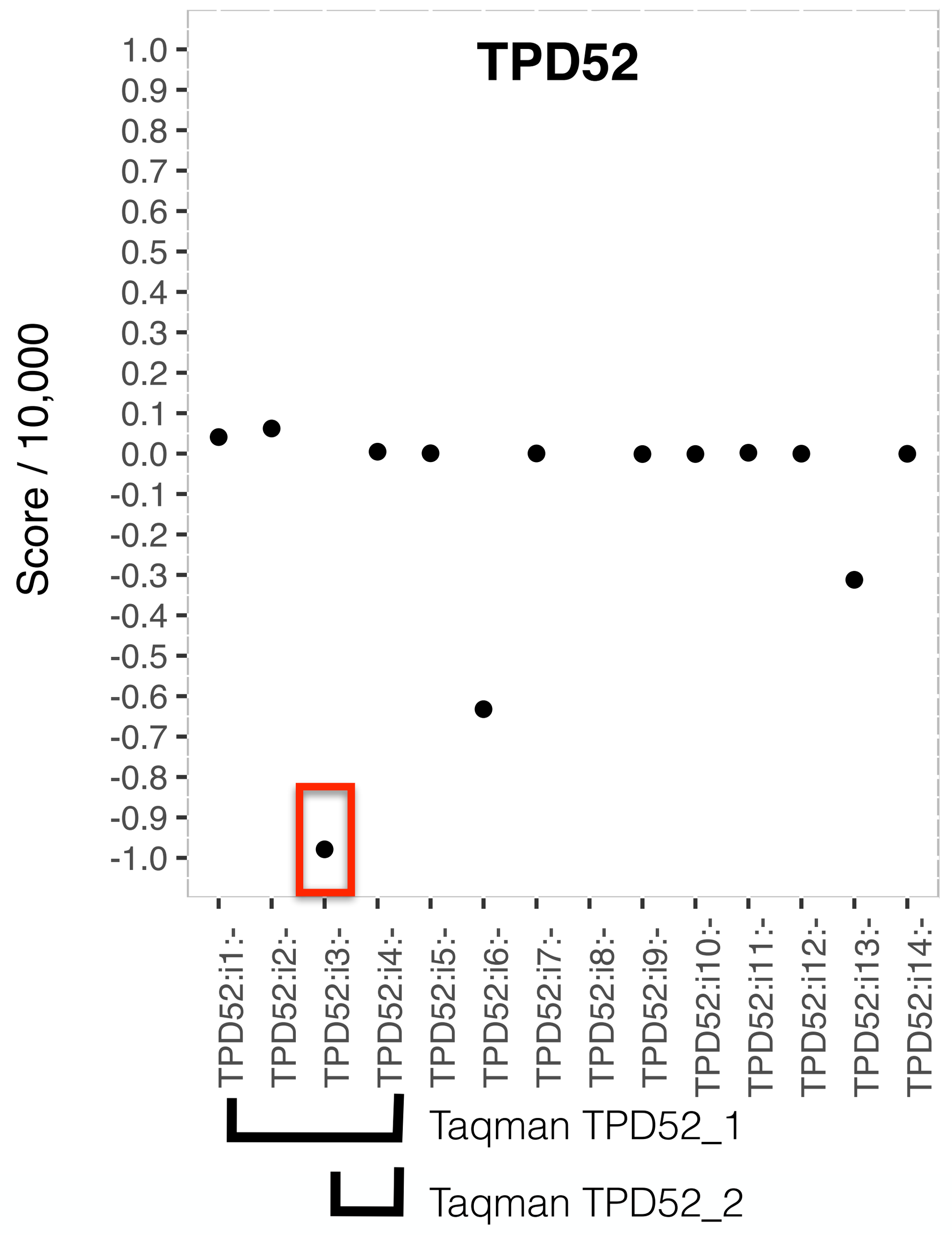

All transcripts detected by TPD52_1: ENST00000379096, ENST00000518937, ENST00000519303

All transcripts detected by TPD52_2: ENST00000537855, ENST00000379097, ENST00000520527, ENST00000517427, ENST00000448733

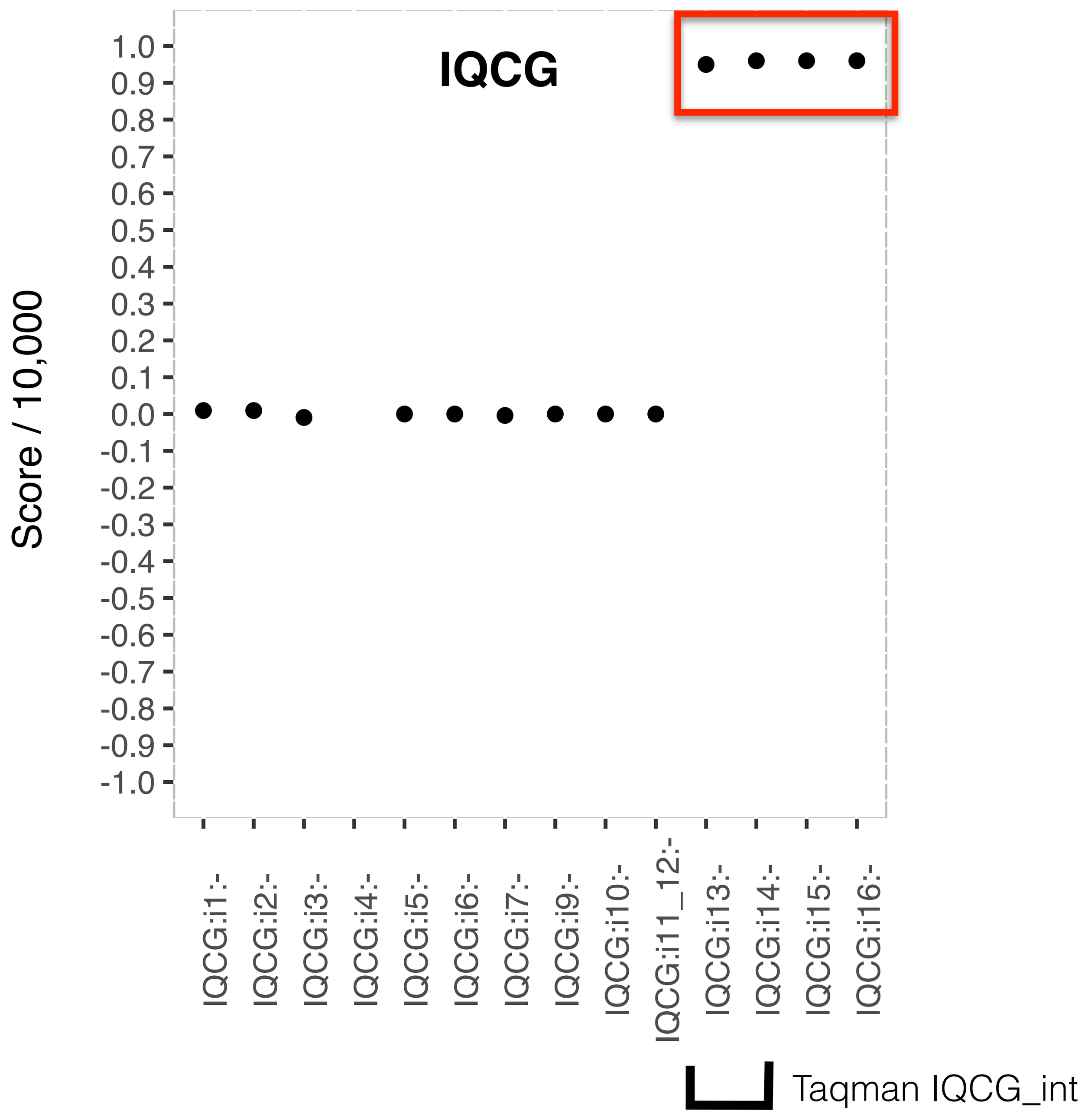

All transcripts detected by IQCG_2: ENST00000478903

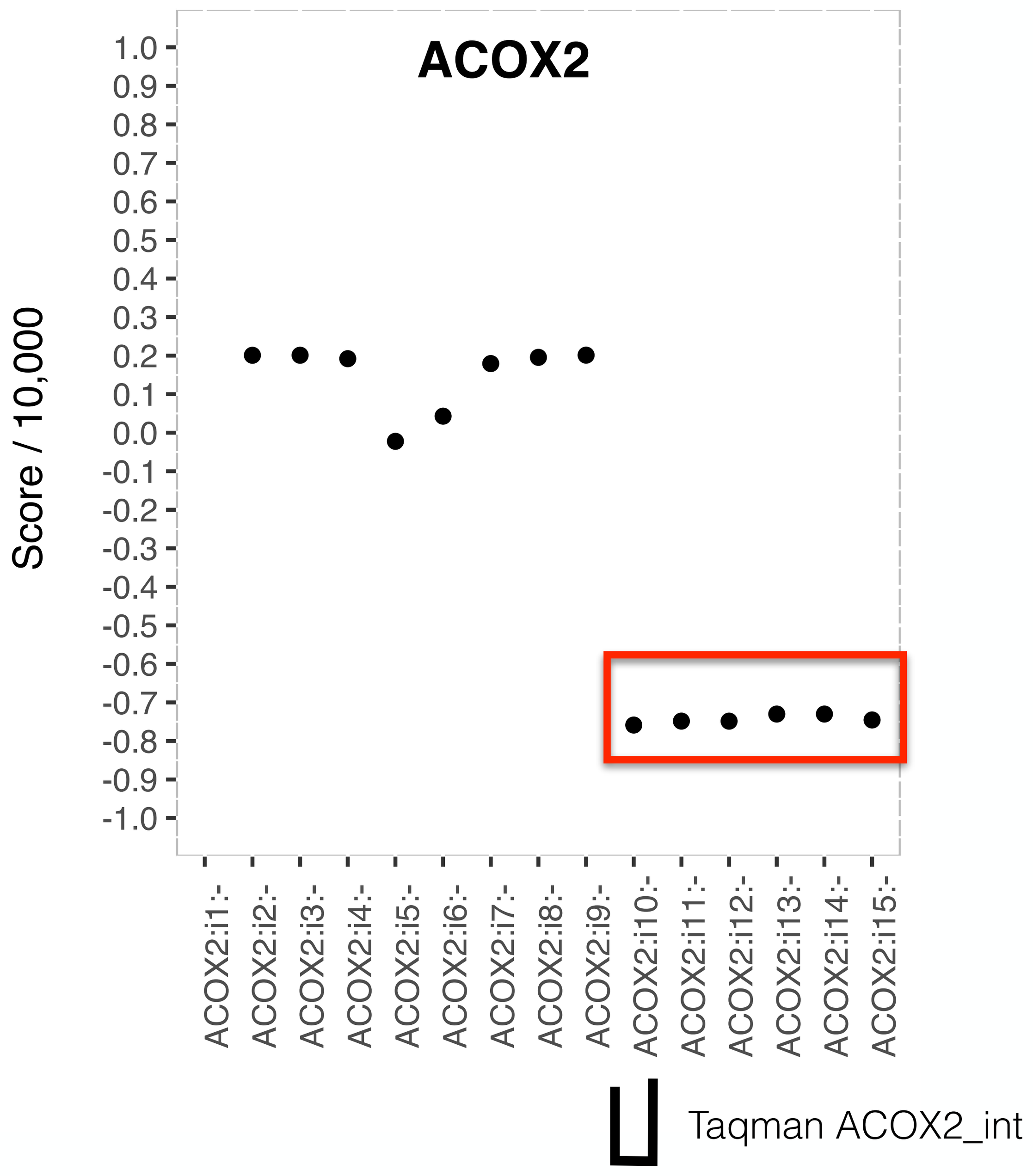

All transcripts detected by ACOX2_2: ENST00000467738

Supplementary Table S18 Radium/Rutgers patient information

Patient	Class	Batch	Mapped reads
B01	Basal	CINJ_29	22284468
B02	Basal	CINJ_29	22079693
B03	Basal	CINJ_29	11133779
B04	Basal	MICMA_29	36813276
B05	Basal	MICMA_29	23469650
B06	Basal	MICMA_29	18506669
B07	Basal	CINJ_100	15854777
B08	Basal	CINJ_100	14767023
B09	Basal	CINJ_100	10006684
B10	Basal	CINJ_100	7966227
B11	Basal	CINJ_100	10011043
B12	Basal	CINJ_100	8299672
B13	Basal	CINJ_100	10034853
B14	Basal	CINJ_100	12077848
B15	Basal	CINJ_100	9036413
B16	Basal	CINJ_100	8479510
B17	Basal	ULL_29	21065092
L01	Luminal	CINJ_29	15584882
L02	Luminal	CINJ_29	16454602
L03	Luminal	CINJ_29	14919771
L04	Luminal	CINJ_29	20559539
L05	Luminal	CINJ_29	18794693
L06	Luminal	CINJ_29	15851829
L07	Luminal	MICMA_29	21662557
L08	Luminal	MICMA_29	20196828
L09	Luminal	MICMA_29	34284885
L10	Luminal	MICMA_29	18826913
L11	Luminal	MICMA_29	21603546
L12	Luminal	MICMA_29	17355696
L13	Luminal	MICMA_29	18974165
L14	Luminal	MICMA_29	21914653
L15	Luminal	MICMA_29	38330315
L16	Luminal	MICMA_29	15664030
L17	Luminal	CINJ_100	12813947
L18	Luminal	CINJ_100	8581186
L19	Luminal	CINJ_100	14374856
L20	Luminal	CINJ_100	10653252
L21	Luminal	CINJ_100	9757295
L22	Luminal	CINJ_100	6635232
L23	Luminal	CINJ_100	8775584

L24	Luminal	CINJ_100	9668356
L25	Luminal	ULL_29	31772255
L26	Luminal	ULL_29	16339027
